# Elusive Origins of the Extra Genes in *Aspergillus oryzae*

**DOI:** 10.1371/journal.pone.0003036

**Published:** 2008-08-22

**Authors:** Nora Khaldi, Kenneth H. Wolfe

**Affiliations:** Smurfit Institute of Genetics, Trinity College, Dublin, Ireland; University of Dayton, United States of America

## Abstract

The genome sequence of *Aspergillus oryzae* revealed unexpectedly that this species has approximately 20% more genes than its congeneric species *A. nidulans* and *A. fumigatus*. Where did these extra genes come from? Here, we evaluate several possible causes of the elevated gene number. Many gene families are expanded in *A. oryzae* relative to *A. nidulans* and *A. fumigatus*, but we find no evidence of ancient whole-genome duplication or other segmental duplications, either in *A. oryzae* or in the common ancestor of the genus *Aspergillus*. We show that the presence of divergent pairs of paralogs is a feature peculiar to *A. oryzae* and is not shared with *A. nidulans* or *A. fumigatus.* In phylogenetic trees that include paralog pairs from *A. oryzae*, we frequently find that one of the genes in a pair from *A. oryzae* has the expected orthologous relationship with *A. nidulans*, *A. fumigatus* and other species in the subphylum Eurotiomycetes, whereas the other *A. oryzae* gene falls outside this clade but still within the Ascomycota. We identified 456 such gene pairs in *A. oryzae*. Further phylogenetic analysis did not however indicate a single consistent evolutionary origin for the divergent members of these pairs. Approximately one-third of them showed phylogenies that are suggestive of horizontal gene transfer (HGT) from Sordariomycete species, and these genes are closer together in the *A. oryzae* genome than expected by chance, but no unique Sordariomycete donor species was identifiable. The postulated HGTs from Sordariomycetes still leave the majority of extra *A. oryzae* genes unaccounted for. One possible explanation for our observations is that *A. oryzae* might have been the recipient of many separate HGT events from diverse donors.

## Introduction

*Aspergillus oryzae* is a filamentous ascomycete used in traditional Japanese soy sauce production, and has important industrial applications as a producer of hydrolytic enzymes in solid-state fermentations [1], [2]. When the *A. oryzae* genome was sequenced it was found to be significantly larger and to contain more genes than the genomes of other species in the genus *Aspergillus* [3]–[5]. At 37 Mb, the *A. oryzae* genome assembly is 23% larger than the *A. nidulans* assembly and 32% larger than the *A. fumigatus* assembly. It is predicted to code for 12,074 proteins of >100 amino acids, which is 1,412 more proteins than *A. nidulans* and 2,444 more than *A. fumigatus* [4]. Although some of the differences in the predicted gene count between species may be due to differences in the bioinformatics methods used by the three sequencing laboratories to annotate the genomes, the larger DNA content of *A. oryzae* is incontrovertible. Moreover, even if one excludes singleton genes (those with no homologs in completed fungal genome databases), which are the ones most likely to be annotation artifacts, *A. oryzae* still contains 16–26% more genes than the other species: the numbers of non-singletons are 12,044, 10,425 and 9,574 for *A. oryzae*, *A. nidulans* and *A. fumigatus* respectively. There is also indirect evidence that most of the predicted genes in *A. oryzae* are functional: sequencing the genome of *A. flavus*, which is a very close relative of *A. oryzae* and has a similarly large genome, shows that 8,953 genes (85% of all orthologs between *A. oryzae and A. flavus*) have a conservative pattern of nucleotide substitution (*K_A_*<*K_S_*) consistent with purifying selection to retain protein-coding capacity (N. Khaldi and G. Payne, unpublished results).

Comparisons of gene order revealed the presence of syntenic blocks common to *A. oryzae*, *A. nidulans* and *A. fumigatus*, as well as genomic blocks specific to *A. oryzae* that lack synteny with the other two species [3]. These non-syntenic blocks share the characteristics of, first, containing genes that seem to exist only in *A. oryzae* (that is, they lack orthologs in *A. nidulans* and *A. fumigatus*) and, second, appearing in a mosaic manner throughout the genome of *A. oryzae*. Machida *et al.* [3] reported that, surprisingly, phylogenetic analysis of some genes from these non-syntenic regions indicated that they are distantly related paralogs of other genes that have a syntenic and apparently orthologous relationships among *A. oryzae*, *A. nidulans* and *A. fumigatus.* Thus, the extra genes in *A. oryzae* did not seem to be the result of recent gene duplications that happened specifically in *A. oryzae* after it diverged from the other two species; rather, they seemed to be the products of much older divergence event(s) [3]. This is particularly surprising because comprehensive genome-based phylogenetic analysis shows that *A. nidulans* is an outgroup to *A. fumigatus* and *A. oryzae* [4], so on that basis the increased gene number in *A. oryzae* would most parsimoniously be explained by a more recent species-specific expansion.

In this study we aim to find out the origin of the *A. oryzae* genes giving rise to this unusual phylogenetic tree topology. This topology was first noticed, but not named, by Machida *et al.* [3]. We refer to it as the “S topology” . We show that the discovery of these trees is not just a subjective observation: there is a genuine statistical excess of trees with the S topology in *A. oryzae* as compared to other *Aspergillus* species. We then explore several possible explanations for these trees. First, we test whether the pattern could have been caused by an ancient whole-genome duplication (WGD) in the common ancestor of the three *Aspergillus* species, similar to the WGD that occurred in an ancestor of *Saccharomyces cerevisiae* [6], [7], followed by a lower rate of gene loss in *A. oryzae* than in *A. nidulans* and *A. fumigatus*. We find no evidence for WGD or similar events such as whole-chromosome duplication (aneuploidy). Second, we consider the possibility that the Topology S trees might be artifacts. If the paralogous pairs in *A. oryzae* were in fact the products of recent (species-specific) gene duplications, but one gene copy subsequently underwent rapid sequence divergence, the ensuing asymmetry of evolutionary rates could cause phylogenetic methods to infer an incorrect tree due to the phenomenon of long-branch attraction between the accelerated branch and the outgroup [8], [9]. Third, we consider the possibility that the extra genes were added to the *A. oryzae* genome by horizontal gene transfer (HGT).

## Results

### An excess of topology S trees

**Figure 1:**
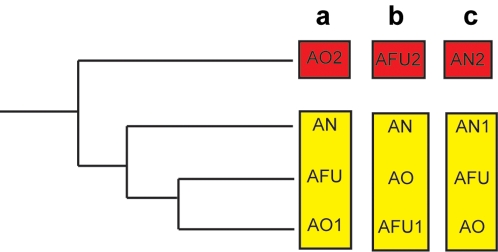
Definition of Topology S loci in (a) *A. oryzae*, (b) *A. fumigatus*, and (c) *A. nidulans*. Yellow boxes indicate the genes that are orthologous in the three species. The red box contains the extra paralog (labeled *AO2*, *AFU2* or *AN2*). Loci having Topology S were identified in each species by an automated process without the use of an outgroup, but the root of the tree is likely to lie on the upper (longest) branch.

Machida *et al.* [3] noted that many gene families are expanded in *A. oryzae* and that phylogenetic trees of these families often show the topology that we have called Topology S. This topology is remarkable because it implies that a pair of paralogous genes (*AO1* and *AO2*; Figure 1a) that are present in *A. oryzae* originated by a gene duplication that occurred before the speciation events that separated *A. oryzae*, *A. nidulans* and *A. fumigatus*. If that interpretation is correct, then orthologs of the *AO2* gene must subsequently have been deleted from the *A. nidulans* and *A. fumigatus* genomes, and the shape of the species phylogeny necessitates that two independent deletions of *AO2* must have occurred in *A. nidulans* and *A. fumigatus*.

We quantified this phenomenon by searching systematically for loci that form a paralogous pair in *A. oryzae* but are single-copy in *A. nidulans* and *A. fumigatus*, and where one of the *A. oryzae* copies is divergent from the other three sequences as expected under Topology S (see Methods). We found 456 such pairs, which accounts for 19% of all pairs of paralogous genes in *A. oryzae*. We refer to these loci as Set S_AO_. For comparison, we likewise defined two analogous sets of loci that contain extra copies in other species. Set S_AFU_ consists of genes that are double-copy only in *A. fumigatus* and have the equivalent of Topology S for that species (Figure 1b). This set contains 202 pairs, which is 11% of all paralog pairs in *A. fumigatus*. Similarly, Set S_AN_ consists of 219 pairs (12% of all paralog pairs) that are duplicated only in *A. nidulans* and have the topology shown in Figure 1c.

The number of loci in Set S_AO_ is significantly greater than the numbers in Set S_AN_ (*P* = 2×10^−10^ by Fisher test) and Set S_AFU_ (*P = *7×10^−12^), whereas there is no significant difference between Sets S_AN_ and S_AFU_ (*P* = 0.43). This result confirms Machida *et al.*'s report that there is an excess of divergent paralogous gene copies in the *A. oryzae* genome.

### Topology S trees are unlikely to be artifacts of rate acceleration after gene duplication

Gene duplication is often followed by a period of accelerated sequence evolution in one or both gene copies [10]–[12]. If the rates are sufficiently unequal, the resulting phylogenetic trees can suffer from long branch attraction [8], [13], an artifact of tree reconstruction methods that causes the longer branches to clump together in the tree regardless of their true phylogenetic relationship. Where one member of a duplicated gene pair has accelerated, attraction between this gene's branch and the outgroup used to root the tree (which is usually also a long branch) can result in an incorrect topology that makes the gene duplication appear older than it actually was. In the case of *Saccharomyces cerevisiae*, this artifact affected more than half of all the duplicate gene pairs that were formed by its WGD [9], [14].

**Figure 2:**
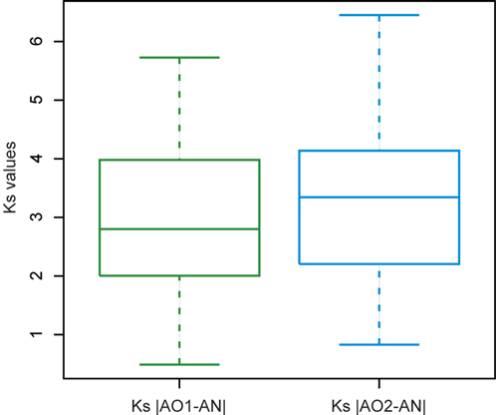
Boxplots of the distributions of synonymous nucleotide divergence (*K*_S_) values between *A. nidulans* genes (*AN*) and *A. oryzae* gene copies *AO1* and *AO2*, for all genes in Set S_AO_. The boxes show the median and interquartile values, with 95% of the data falling within the whiskers. *K*_S_ values were calculated using yn00 from the PAML package [40]. *K*_S_ values for the *AO1* genes are significantly lower than for the corresponding *AO2* genes (*P*<10^−4^; Wilcoxon test).

To test whether the *A. oryzae* Topology S trees are the result of a similar artifact, we compared the levels of synonymous nucleotide divergence (*K_S_*) between each of the *A. oryzae* genes (*AO1* and *AO2*) and their single homolog (*AN*) in *A. nidulans*. Rate acceleration after gene duplication is expected to affect synonymous divergence to a much lesser extent than nonsynonymous divergence, if at all. If the duplication that produced *AO1* and *AO2* was a species-specific event in *A. oryzae* (*i.e.*, the Topology S trees are artifacts) then the synonymous distances |*AO1*-*AN*| and |*AO2*-*AN*| should be equal, and the excess amino acid sequence divergence of *AO2* could be attributed to accelerated protein evolution. Alternatively, if *AO2* truly branched off before the *A. oryzae*-*A. nidulans* divergence (*i.e.*, the Topology S trees are correct) then the synonymous distance |*AO1*-*AN*| should be less than the synonymous distance |*AO2*-*AN*| (Note that *AO2* is always defined as the *A. oryzae* gene that lacks an apparent *A. nidulans* ortholog; Figure 1a). We find that the synonymous divergence is indeed higher for the *AO2* genes: the median *K_S_* values are 3.35 for |*AO2*-*AN*| and 2.87 for |*AO1*-*AN*| (*P*<10^−4^ by Wilcoxon test; Figure 2). This result indicates that the excess of genes in Set S_AO_ is not an artifact of long branch attraction.

One possible caveat with the above analysis is that, if the *AO2* genes are expressed at lower levels than their *AO1* homologs, then codon bias in the *AO1* genes might decrease the apparent level of synonymous divergence from *A. nidulans* [15]. It is known that *A. oryzae* genes located in genomic regions that are not syntenic to other species have lower average expression levels than those in syntenic blocks [3]. However, this expression difference is due primarily to the low expression of genes in non-syntenic blocks that lack homologs elsewhere in the genome, and not to *AO2*-type genes [16]. Using codon bias as a proxy for gene expression levels, we found that the frequency of optimal codons (Fop) is slightly higher in *AO1* genes than in their *AO2* homologs (median Fop values 0.4290 and 0.3945 respectively), which is suggestive of higher expression but the difference is not statistically significant (Wilcoxon test of the hypothesis that the ratio Fop(AO1)/Fop(AO2) in each locus is greater than 1; P = 0.25). We doubt that the difference in Fop values is sufficient to cause the difference in *K_S_* values observed in Figure 2, but are unable to test this directly.

### No evidence for ancient WGD in *Aspergillus*

**Figure 3:**
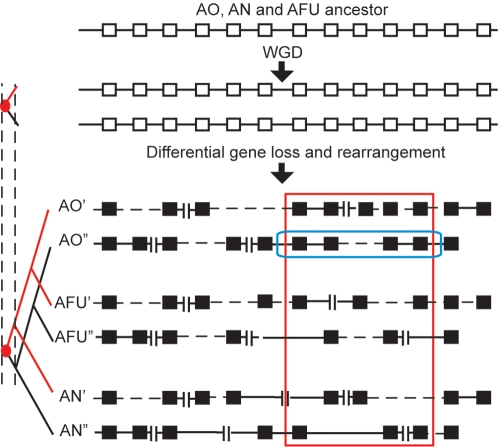
Model of a WGD hypothesis for the origin of extra genes in *A. oryzae*. A WGD in the common ancestor of the three *Aspergillus* species, followed by extensive gene losses in both *A. nidulans* and *A. fumigatus* could potentially account for the large number of genes in Set S_AO_. In this schematic diagram black squares represent genes, double vertical lines represent chromosome breaks, and dashed horizontal lines represent gene deletions or intrachromosomal rearrangements. Our method of testing for WGD involves sliding a window (blue box) through the *A. oryzae* genome to find clusters of nearby genes that are enriched in genes from Set S_AO_ (red box). If a WGD occurred, the paralogs of the genes in the blue window are expected to be closer together on the sister *A. oryzae* genomic region than expected by chance.

Another possible scenario that might explain the extra genes in *A. oryzae* is an ancient whole genome duplication (WGD) prior to the speciation of *A. oryzae*, *A. nidulans* and *A. fumigatus*, followed by parallel independent losses of most of the duplicated genes in the latter two species (Figure 3). Although this scenario might seem unlikely – and indeed Machida *et al*. [3] did not observe any long duplicated segments in the *A. oryzae* genome – it is a formal possibility so we searched rigorously for evidence of it.

The WGD events described so far in other genomes were detected using a number of different methods [reviewed in 17]. Patterns in the chromosomal locations of paralogs give by far the clearest signals in situations where a WGD is present in a sequenced genome but no genome sequence from a closely-related unduplicated outgroup species is available [6], [18]–[21]. Immediately after a WGD, the genome consists of pairs of identical chromosomes containing identical genes in the same order. This pattern will gradually become eroded by gene losses and chromosomal rearrangements, but we would expect that even an old WGD should leave a signal: the order of genes along a given chromosomal region should be correlated with the order of their paralogs along some other region in the genome. In other words, paralogs should not just be randomly distributed with regard to one another.

**Figure 4:**
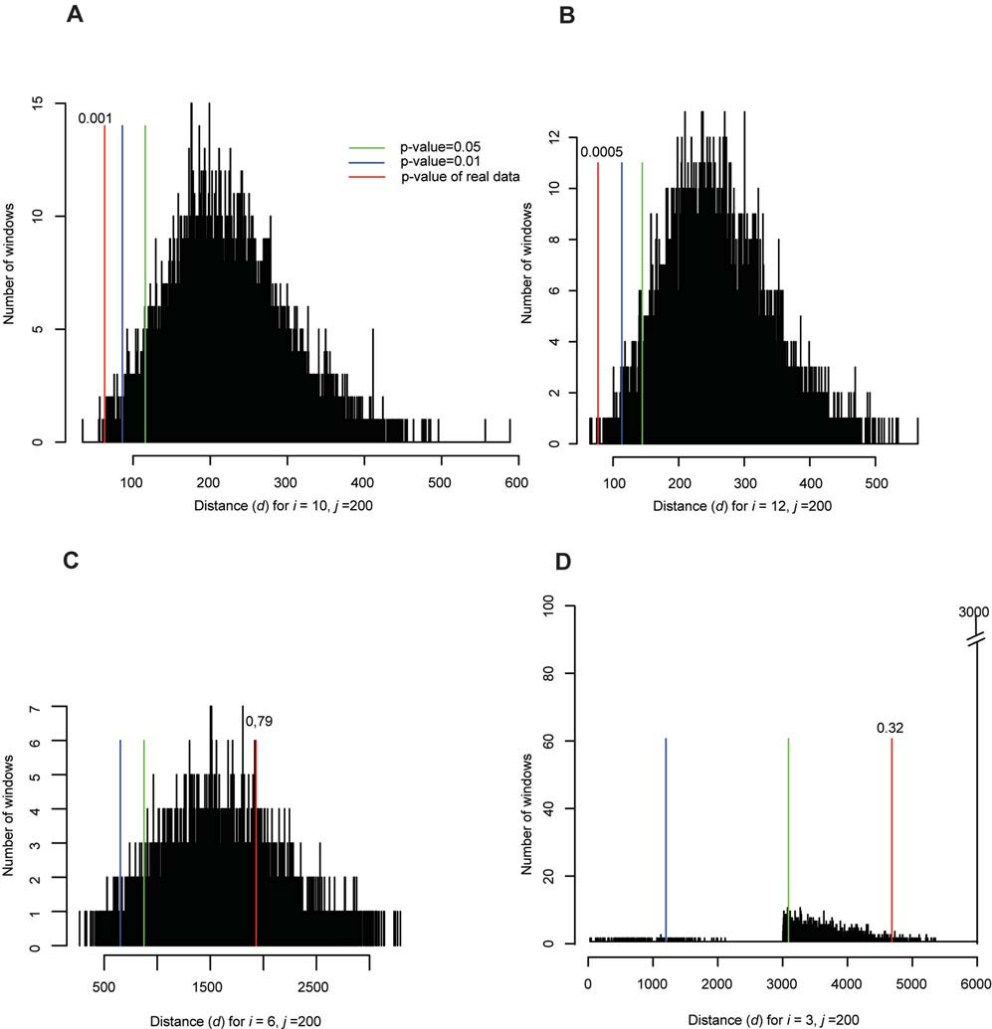
Testing for whole genome duplication in *A. oryzae* and *S. cerevisiae*. Black histograms show the distribution of distance scores (*d*) obtained in 10,000 simulations where paralog locations were randomized. Blue and green lines show the cutoff points for the most-clustered 1% and 5% of these data, respectively. Red lines show the observed *d* values for *S. cerevisiae* or *A. oryzae*. All the graphs are for windows of size *j* = 200. Panels A-C consider only windows where the paralogous genes are all on the same chromosome. (A) Results for *S. cerevisiae* for windows with *i* = 10 paralogs. (B) Results for *S. cerevisiae* for windows with *i* = 12 paralogs. (C) Results for *A. oryzae* for windows with *i* = 6 paralogs. (D) Results for *A. oryzae* for windows with *i* = 3 paralogs, including a penalty of 3000 for each interchromosomal rearrangement event.

To compare the distribution of locations of paralogous genes in the *A. oryzae* genome to random expectations we defined a distance measure, *d*, of the extent to which genes that are close together in a genome also have paralogs that are close together somewhere else in the genome (see Methods). We estimated the statistical significance of the observed distances in *A. oryzae* by comparing it to the distribution of distances obtained in computer simulations where gene locations were shuffled randomly (Figure 4).

We applied this method to both *S. cerevisiae* and *A. oryzae*. The method successfully detects a strong signal of non-random locations of paralogous gene pairs in *S. cerevisiae*, a species known to have undergone WGD (empirical *P*<10^−3^; Figure 4A,B), but there is no significant signal of WGD in the *A. oryzae* genome (*P* = 0.79; Figure 4C). This result not only argues against WGD but also against segmental duplication which would be expected to leave a similar, though weaker, signal. We also used a modified measure to permit consideration of situations where two genes on one chromosome have paralogs on two different chromosomes (see Methods) but again found no evidence of ancient WGD in *A. oryzae* (Figure 4D).

In an independent analysis we carried out all-against-all BLASTP searches within the protein sets of each of *A. oryzae*, *A. nidulans* and *A. fumigatus*. We then made a dot matrix plot for each species showing the chromosomal locations of reciprocal-best-hit pairs within each genome. In previous studies on *S. cerevisiae* and plant species, approaches similar to this revealed strong diagonals corresponding to paralogous chromosomal segments that were formed by WGD [6], [21], [22]. In contrast, inspection of our plots for *Aspergillus* species did not reveal any indication of WGD or segmental duplications, in agreement with the previous report for *A. oryzae* [3].

### Can the excess of Topology S genes be explained by Horizontal Gene Transfer?

Taken together, the above results show that the excess of *A. oryzae* genes in Set S_AO_ cannot be explained satisfactorily by recent gene duplications in *A. oryzae*, nor by an ancient WGD or segmental duplications in an *Aspergillus* ancestor. We therefore examined the third possibility of HGT into *A. oryzae*.

To detect a possible donor taxon, we first analyzed the phylogenetic trees of a random sample of genes from Set S_AO_. This showed that the extra (*AO2*) copies of most genes are nested in the Ascomycota kingdom, so if HGT occurred the donor should also be in the Ascomycota. To provide a common reference point for phylogenetic reconstructions, we imposed the basidiomycete *Ustilago maydis* [whose genome is completely sequenced; 23], as the outgroup for all trees. This reduced our Set S_AO_ to a subset Q_AO_ of 122 genes for which an ortholog in *U. maydis* could be identified unambiguously by a reciprocal-best-BLAST-hits (RBH) approach.

**Figure 5:**
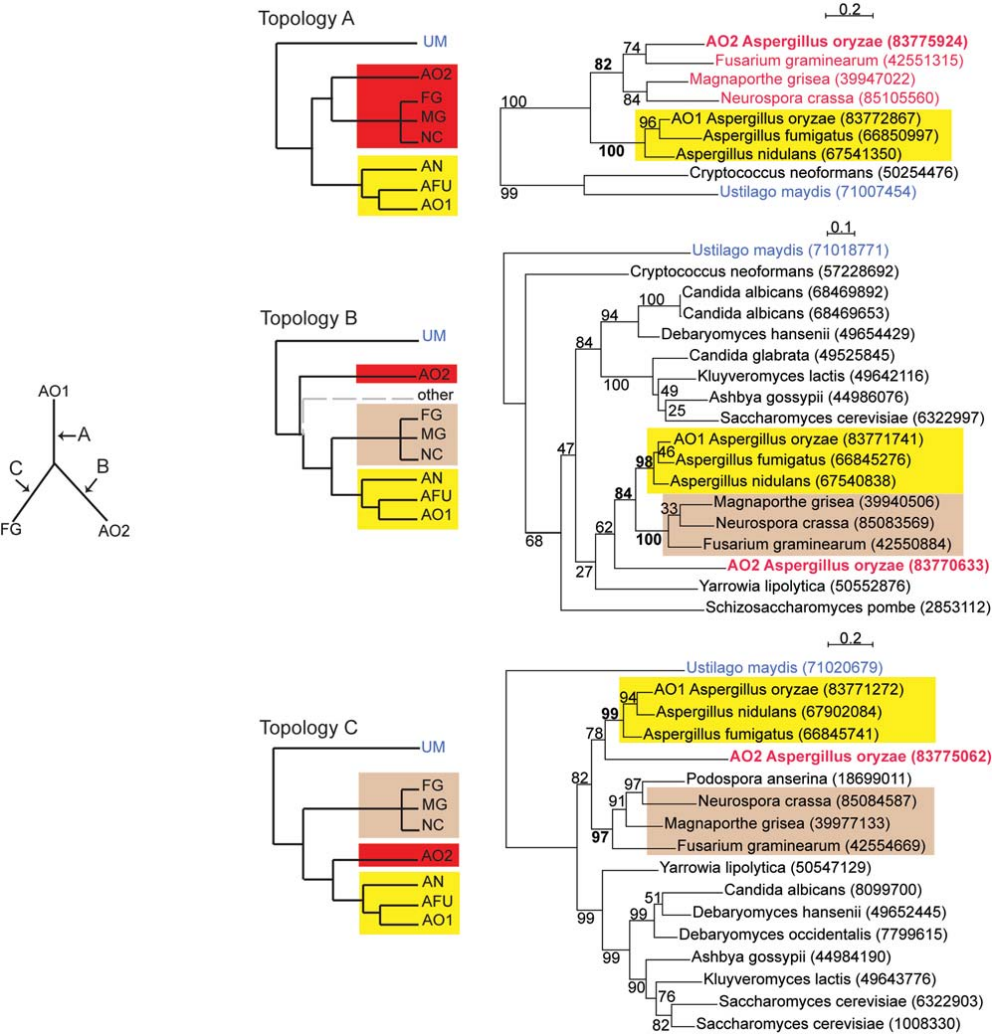
Topologies A, B and C for rooted trees. *Left*: The three topologies correspond to the three possible points for placement of a root (arrows labeled A, B, C) onto an unrooted tree consisting of a gene pair from *A. oryzae* and their homolog from a Sordariomycete such as *Fusarium graminearum*. *Center*: Rooted representations of the topologies. *Right*: real examples of *A. oryzae* genes classified in each of the three topologies. Numbers in parentheses are NCBI protein identifiers. Trees were constructed using PHYML with bootstrap percentages (form 500 replicates) shown. The main groups differentiating the topologies from each other are colored.

We constructed rooted phylogenetic trees for the 122 genes in Set Q_AO_ with their homologs in other fungi (primarily Sordariomycetes), and sorted them manually into three mutually exclusive topology types depending on the position of the *AO2* gene (Types A, B and C; Figure 5; Supplementary Figure S1). In all three topologies the *AO1* gene of *A. oryzae* is clustered in a monophyletic group with its orthologs from *A. nidulans* and *A. fumigatus*, and in all three the species in the subphylum Sordariomycetes (*e.g.*, *Neurospora crassa*, *Fusarium graminearum*, *Magnaporthe grisea*) form a monophyletic group. Type A trees are those where the second *A. oryzae* copy *AO2* clusters with Sordariomycete species to the exclusion of the genus *Aspergillus* (Figure 5, upper). Type B trees have a topology in which *AO2* lies outside both the *Aspergillus* genus and the Sordariomycetes (Figure 5, center). Type C trees are those where *AO2* forms a sister group to the clade of *Aspergillus* orthologs that includes *AO1*, with Sordariomycete sequences outside this pair (Figure 5, lower). Of these three topologies, only the Type A trees are directly suggestive of HGT because they implicate an identifiable donor lineage.

There are only three possible topologies for a phylogenetic tree that consists of an outgroup and three ingroup taxa. The A, B, and C trees correspond to these three topologies; the ingroup clades are *AO2*, the *Aspergillus* clade that includes *AO1*, and the Sordariomycete clade (Figure 5, *left*). No other topologies can exist for our data unless the Sordariomycete or *Aspergillus* clades are broken up.

The sorting process for trees in Set Q_AO_ was done manually and with reference to the bootstrap support values for critical branches on the tree (we required a bootstrap value of ≥70% for topology-defining branches). To keep the process simple we rejected trees from complex gene families or with poor bootstrap support, and we permitted minor deviations from the expected species phylogeny for the *Aspergillus* (*AO1*) and Sordariomycete clades. We also classified trees where the *AO2* gene was placed within the Sordariomycetes, rather than as sister to Sordariomycetes, as Type A. These additional restrictions left only 35 classifiable trees from Set Q_AO_, of which 12 were Type A, 9 were Type B, and 14 were Type C (Table 1 and Supplementary Table S1). Likelihood Ratio Tests on these trees confirmed that monophyly of the *AO1* and *AO2* genes could be rejected at α<0.0001. The fact that we find approximately equal numbers of trees of the three possible types is not consistent with the hypothesis that all *AO2* genes originated by HGT from the same donor. Instead, the result suggests that the *AO2* genes are heterogeneous in terms of their origins: if they all arose by HGT then the HGT events involved multiple donors, or if they all arose by gene duplication then those duplications occurred at multiple times.

**Table 1:** Numbers of phylogenetic trees with Topologies A, B and C obtained from genes in Sets Q_AO_, Q_AN_ and Q_AFU_.

Topology	Number of trees		
	*A. oryzae* (Set Q_AO_)	*A. nidulans* (Set Q_AN_)	*A. fumigatus* (Set Q_AFU_)
A	12	0	0
B	9	9	2
C	14	2	4

For comparison, we made similar analyses in *A. nidulans* and *A. fumigatus*. We compiled two other sets of genes that are analogous to Set Q_AO_ but have duplications only in *A. nidulans* (Set Q_AN_, 40 genes), or only in *A. fumigatus* (Set Q_AFU_, 28 genes), and that give phylogenetic trees that place the second gene (*AN2* or *AFU2*) outside a monophyletic group containing one gene from each of the three *Aspergillus* species. As expected, these gene sets are smaller than the corresponding set with duplication in *A. oryzae*. After phylogenetic tree construction we find that sets Q_AN_ and Q_AFU_ both present an absence of Topology A trees (Table 1). Although the numbers of classified trees are small (11 in Set Q_AN_ and 6 in Set Q_AFU_) this deficit contrasts with the number of Topology A trees seen in the *A. oryzae* set. Topology A trees are the only ones where a putative HGT donor lineage can be identified (Figure 5), so it is striking that trees with this topology are found only in *A. oryzae*. If HGT from Sordariomycetes to *A. oryzae* accounts for the type A topologies, we can estimate that it maximally accounts for one-third of the extra genes in *A. oryzae* (that is, by extrapolation from the 12/35 proportion found in the loci that were classifiable).

It is also notable that among the 12 *A. oryzae* gene pairs that give Topology A trees, the trees for 10 of these pairs place the *AO2* gene on a branch *within* the Sordariomycetes as opposed to lying sister to the Sordariomycetes. An example is the Topology A gene in Figure 5, where the *AO2* sequence (NCBI identifier 83775924) clusters specifically with *F. graminearum*. Of the 10 genes that showed this property, five clustered with *F. graminearum*, three with *M. grisea* and two with *N. crassa*. A single consistent candidate donor lineage is again lacking, even within the Sordariomycetes.

### Functions and physical clustering of *AO2* genes with Topology A within the *A. oryzae* genome

**Figure 6:**
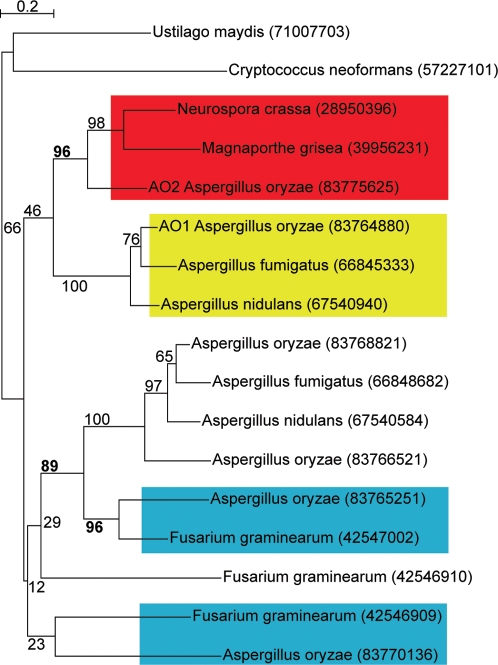
A case of family expansion due to apparent HGT. The gene family is a member of the metal dependent hydrolytic enzyme superfamily (amidases, aminoacyclases, and carboxypeptidases). The *A. oryzae* genes *AO1* and *AO2* originally identified in our automated search are labeled. Four of the six *A. oryzae* genes (83775625, 83765251, 83766521, 83770136) in this family lack orthologs in *A. nidulans* and *A. fumigatus*, and for the first two of these there is strong bootstrap support for HGT from Sordariomycete. Of these four genes, three show Topology A and one (83766521) shows Topology C relative to their nearest Sordariomycete homologs.

The set of 12 *A. oryzae* genes with Topology A is enriched in particular functions, most notably the hydrolytic traits that are characteristic of *A. oryzae* (Supplementary Table S1). This species breaks down starch into sugars, and proteins into amino acids, by producing vast amounts of hydrolytic enzymes. The enrichment of some families in these classes among duplicated *A. oryzae* genes is striking, as previously also noted by Machida *et al.* [3]. For example, gene AO090103000299 (gi 83775625) is a carboxypeptidase that our method identified as an *AO2* gene giving Topology A. Further exploration of this gene family (Figure 6) shows that it is a member of a six-gene family in *A. oryzae*, only two of which have orthologs in *A. nidulans* and *A. fumigatus*. Indeed this family consists of only two genes in the latter two species. Of the four other *A. oryzae* genes in the family, three cluster with Sordariomycete genes in the phylogenetic tree (two with different *F. graminearum* genes, and one (the gene AO090103000299 originally identified as an *AO2* candidate) with a *M. grisea-N. crassa* pair. In this *A. oryzae* gene family, only two genes were inherited vertically and are shared with other *Aspergillus* species, two (possibly three) originated by HGT from Sordariomycetes, and the sixth gene has an undetermined origin. Thus, HGT appears to have played a major role in the expansion of this carboxypeptidase family in *A. oryzae*.

Comparison to the genome sequence of *A. flavus* (G. Payne *et al*., unpublished), which has a recent and clonal relationship to *A. oryzae* [24], shows that many of the *AO2* genes have orthologs in that species. Therefore, the *AO2* genes were gained by *A. oryzae* after it diverged from *A. fumigatus* but before the split between *A. flavus* and *A. oryzae*.

If foreign DNA enters a recipient genome in a single event, the foreign genes might be expected to exhibit some clustering in the recipient genome. Conversely if they arrived in independent events, or if the hypothesis of HGT is incorrect, they should exhibit random genomic locations. If an HGT event is very recent, the transferred genes may also still be clustered in the donor genome. We applied the method of Lee and Sonnhammer [25] to test whether the locations of the *AO2* genes that may have undergone HGT from Sordariomycetes show significant physical clustering in the *A. oryzae* genome. For the 12 *AO2* genes with tree topology A (Supplementary Table S1), the clustering score is 56, as compared to a mean score of 21.7 for 100,000 replicates with randomized data (empirical *P* = 0.02). In contrast, the *AO1* partners of these genes do not show physical clustering (score = 6.6; *P* = 0.95), nor did we find significant clustering in comparisons of the *A. oryzae AO2* genes that gave B, C, or both B and C topologies (*P* = 0.19; *P* = 0.06 and *P* = 0.19 respectively). This result again highlights the distinctive nature of the *AO2* genes with tree topology A, in terms of both their phylogenetic and genomic placement. The physical clustering of the Topology A *AO2* genes is indicative of either a one-time transfer event, or a preference for integrating new genes into the genome at particular sites. We did not detect significant clustering in the *F. graminearum* genome of homologs of the *AO2* genes that form branches specifically with *F. graminearum* (though the dataset is small; only 5 genes).

## Discussion

A frustrating feature of our study was the extent of data loss that we encountered as the analysis progressed. The *A. oryzae* genome contains more than one thousand extra genes as compared to *A. nidulans* and *A. fumigatus*, but when we attempted to use phylogenetic methods to determine the origin of these genes the dataset collapsed to only 35 informative loci (Table 1). One major cause of the reduction of the dataset is that we were only able to work with genes that are duplicated in *A. oryzae*. The reason for this is pragmatic: we cannot actually identify which, out of the 12,074 genes in *A. oryzae*, are the ‘extra’ ones, except in the 456 cases where the *AO1* gene was retained too (Set S_AO_). Moreover, our method ignored recent species-specific duplications in all species. A second problem was the lack of identifiable basidiomycete outgroup sequences for many of the genes that are duplicated in *A. oryzae*. It is difficult to speculate why these sequences do not exist, but one possibility is that the set of genes that has been added to the *A. oryzae* genome tends to be derived from the fastest-evolving, and hence ascomycete-specific, portion of the fungal genome [26], [27].

We were able to rule out annotation error and WGD as possible sources of the extra genes in *A. oryzae* relative to other *Aspergillus* species. Our phylogenetic analysis also rules out species-specific gene duplication events in *A. oryzae* as the source of many (456) of these genes. These results leave only two viable possibilities for the origin of the extra genes: HGT, or older single-gene duplications (in the common ancestor of the three *Aspergillus* species) followed by independent parallel losses in *A. nidulans* and *A. fumigatus*. If multiple single-gene duplications occurred, then the level of retention of ancient duplicated genes must have been much higher in *A. oryzae* than in the other two *Aspergillus* species, and parallel losses of the same genes must have occurred in *A. nidulans* and *A. fumigatus*. This scenario seems unparsimonious, but it is not impossible and it may have occurred in a systematic manner if – for example – there were large differences in the effective population sizes of the species [28].

Parallel losses in *A. nidulans* and *A. fumigatus* may therefore account for some of the extra content in *A. oryzae*. However, barring phylogenetic tree error, parallel loss is specifically ruled out in the case of the 12 loci with Topology A (Figure 5 and Supplementary Table S1). This result seems to leave HGT as the most viable option for the source of much of the increased gene content of *A. oryzae*, which makes it all the more puzzling that we were unable to identify a consistent donor lineage. Instead, if we accept that all the tree topologies are correct, we must postulate that *A. oryzae* has received genes from at least five different donor lineages: one lineage that diverged prior to the Eurotiomycetes/Sordariomycetes split (giving Topology B trees), one lineage that is in Eurotiomycetes but outside *Aspergillus* (Topology C), and three separate Sordariomycete lineages that have specific affinities to *F. graminearum*, *N. crassa* and *M. grisea* respectively (accounting for the heterogeneity within the Topology A trees). We can speculate that some aspect of the lifestyle or physiology of *A. oryzae* (and *A. flavus*) could make it more able to take up foreign DNA by HGT than other *Aspergillus* species.

Many previous published examples of HGT have involved genes that were transferred between distantly related kingdoms, such as from bacteria to eukaryotes [29]–[34]. In many of these cases the gained genes were inferred to have changed the lifestyle of the recipient species markedly. In our case we are studying a more local exchange between reasonably closely related genomes that already share quite similar gene contents and functions. Our method of analysis necessitates that all the genes we identify as putative gains by HGT result in gene family expansion rather than in the acquisition of new biochemical functions. It is tempting to speculate that HGT helped *A. oryzae* adapt to some new evolutionary niche, but based on our analysis we can only assert that HGT enhanced the hydrolytic and fermentation abilities of this species. We can infer that, because these foreign genes were taken up and maintained in the genome, they contributed in some way to increasing *A. oryzae*'s evolutionary fitness, but we do not know exactly what that contribution was.

## Methods

### Data

Genome sequences and annotations of *A. nidulans*, *F. graminearum* and *U. maydis* were downloaded from the Broad Institute (http://www.broad.mit.edu/annotation/fgi/); *A. fumigatus* from TIGR (http://www.tigr.org/tdb/e2k1/afu1/); and *A. oryzae* from the DDBJ database (accession numbers AP007150-AP007177). BLAST searches against the *A. flavus* genome were performed on http://gaplabg5.cfr.ncsu.edu/blast/blast.html.

### Identification of Sets S_AO_, S_AN_ and S_AFU_

We accepted as triplet sets of orthologous genes in *A. oryzae*, *A. nidulans* and *A. fumigatus*, those genes that were best mutual BLASTP hits (*i.e*., the protein pairs AO1-AN, AO1-AFU, and AFU-AN were all bidirectional best BLASTP hits between their respective genomes, using NCBI BLASTP with default settings). We then tested whether a paralog of the *A. oryzae* gene was present. To identify pairs of paralogs in *A. oryzae* that satisfy topology S, we required that (*i*) the *A. nidulans* and *A. fumigatus* genes in the triplet do not have paralogs produced by species-specific duplications, and (*ii*) the amino acid sequence divergences between the three orthologs in the triplet were all lower than the distance between each of the orthologs and the *A. oryzae* paralog. If these conditions were met, the genes were retained for phylogenetic analysis and the *A. oryzae* orthologous and paralogous copies were termed *AO1* and *AO2* respectively. An automated search using these criteria resulted in the Set S_AO_ of 456 loci that are duplicated only in *A. oryzae* and have topology S. We then used phylogenetic methods to verify the topology of the trees in Set S_AO_. Using the likelihood ratio test, we tested whether the unrooted topology ((AO2,AN)(AO1,AFU)) has a significantly higher likelihood than both of the two possible alternative topologies for the four sequences. We found that at a cutoff of α = 0.05, the expected tree was significantly more likely at 443 of the 456 loci, and at the remaining 13 loci no other topology was significantly better than the expected. We retained all 456 loci for further analysis. Analogous methods were used to identify Sets S_AN_ and S_AFU_. Synonymous and nonsynonymous sequence divergence was calculated using the yn00 program in the PAML package [35]. Fop values were calculated using CodonW (http://mobyle.pasteur.fr) with its predefined set of optimal codons for *A. nidulans*.

### Testing for WGD

To evaluate the hypothesis that the pairs of genes in Set S_AO_ could be the products of a whole-genome duplication, we tested whether these pairs tend to be located on sister genomic regions. If a WGD occurred and produced two pairs of duplicates, A′ and A″, B′ and B″, we would expect that if A′ and B′ are physically close together in the genome, their paralogs A″ and B″ should also be close together somewhere else in the genome. We examined whether the Set S_AO_ paralogs in the *A. oryzae* genome tend to be arranged in this pattern, relative to random expectations. In this analysis we did not take into account the distinction between the *AO1* and *AO2* members of the *A. oryzae* pairs (which have and do not have, respectively, orthologs in *A. nidulans* and *A. fumigatus*). We used a sliding window approach to find regions of relatively high paralog density in *A. oryzae*. We considered windows of size *j* genes (*j* = 200 genes in the examples shown in Figure 4), and counted how many genes in Set S_AO_ they contained. We then retained only the denser windows for further analysis: those with some number *i* genes in Set S_AO_ (*i* = 6 in Figure 4C). For each window with a particular value of *i*, we then tried to measure the physical distance (in units of genes) spanned by their paralogs elsewhere in the *A. oryzae* genome. In many cases the paralogs were not all on the same chromosome. For those windows whose paralogs were all on the same chromosome, we computed the total chromosomal distance *d* occupied by the paralogous windows. We then evaluated the statistical significance of the observed value of *d* by comparing it to the empirical distribution of values obtained in 10,000 computer simulations where the locations of the paralogous genes were randomized. We carried out a parallel analysis on *S. cerevisiae* as a positive control, using the 551 gene pairs from Byrne and Wolfe [36] in place of Set S_AO_. In a second analysis, we also included data from Set S_AO_ genes whose paralogs were located on different *A. oryzae* chromosomes, by adding a fixed penalty to the *d* value in these cases (Figure 4D). The penalty was set at 3000, which is approximately the average number of genes on an *A. oryzae* chromosome. Experimenting with different values of *j* and *i* (data not shown) did not reveal any statistically significant evidence of WGD for *A. oryzae*, whereas the results for *S. cerevisiae* were consistently significant.

### Testing for HGT

We used the basidiomycete *U. maydis* as an outgroup for all phylogenetic analyses. Among the 456 loci in Set S_AO_, we retained a subset (Q_AO_) of 122 loci for which the same *U. maydis* protein was identified as the reciprocal best BLASTP hit of the S_AO_ set members from all three *Aspergillus* species. We then used BLASTP to identify additional fungal homologs of the Set Q_AO_ genes pairs in the NCBI nonredundant protein sequence database. Proteins were aligned using ClustalW [37], and Gblocks [38] was used to remove poorly aligned positions and regions. Maximum likelihood trees were constructed using PHYML [39] with the JTT amino acid substitution matrix and four categories of substitution rates. Bootstrapping was done using PHYML's default options with 100 replicates per run. The trees for each gene were inspected manually to detect common themes and classified into the three possible mutually exclusive topologies (Figure 5). Analogous methods were used to make and study Sets Q_AN_ and Q_AFU_.

## Supporting Information

Table S1 Names and putative functions of A. oryzae gene pairs showing Topologies A, B or C.
(0.03 MB DOC)

Figure S1 Trees classified as Types A, B and C in each Aspergillus species. Trees were constructed using PHYML as described in Methods. In each tree, the sequences identified as AO1 and AO2 (for duplications A. oryzae), AN1 and AN2 (for duplications in A. nidulans), or AFU1 and AFU2 (for duplications in A. fumigatus) are labeled. NCBI identifier (GI) numbers for each sequence are shown.
(0.93 MB PDF)

## References

[1] Hamada T, Fukushima Y, Motai H (1992). Continuous production of soy sauce in a bioreactor.. *Applications of Biotechnology to Traditional Fermented Foods* Report of an Ad Hoc panel of the Board on Science and Technology for International Development Office of International Affairs, National Research Council.

[2] Kobayashi T, Abe K, Asai K, Gomi K, Juvvadi PR (2007). Genomics of *Aspergillus oryzae*.. Biosci Biotechnol Biochem.

[3] Machida M, Asai K, Sano M, Tanaka T, Kumagai T (2005). Genome sequencing and analysis of *Aspergillus oryzae*.. Nature.

[4] Galagan JE, Calvo SE, Cuomo C, Ma LJ, Wortman JR (2005). Sequencing of *Aspergillus nidulans* and comparative analysis with *A. fumigatus* and *A. oryzae*.. Nature.

[5] Nierman WC, Pain A, Anderson MJ, Wortman JR, Kim HS (2005). Genomic sequence of the pathogenic and allergenic filamentous fungus *Aspergillus fumigatus*.. Nature.

[6] Wolfe KH, Shields DC (1997). Molecular evidence for an ancient duplication of the entire yeast genome.. Nature.

[7] Kellis M, Birren BW, Lander ES (2004). Proof and evolutionary analysis of ancient genome duplication in the yeast *Saccharomyces cerevisiae*.. Nature.

[8] Felsenstein J (1978). Cases in which parsimony and compatibility methods will be positively misleading.. Syst Zool.

[9] Fares MA, Byrne KP, Wolfe KH (2006). Rate asymmetry after genome duplication causes substantial long-branch attraction artifacts in the phylogeny of *Saccharomyces* species.. Mol Biol Evol.

[10] Lynch M, Conery JS (2000). The evolutionary fate and consequences of duplicate genes.. Science.

[11] van de Peer Y, Taylor JS, Braasch I, Meyer A (2001). The ghost of selection past: rates of evolution and functional divergence of anciently duplicated genes.. J Mol Evol.

[12] Conant GC, Wagner A (2003). Asymmetric sequence divergence of duplicate genes.. Genome Res.

[13] Delsuc F, Brinkmann H, Philippe H (2005). Phylogenomics and the reconstruction of the tree of life.. Nat Rev Genet.

[14] Langkjaer RB, Cliften PF, Johnston M, Piskur J (2003). Yeast genome duplication was followed by asynchronous differentiation of duplicated genes.. Nature.

[15] Sharp PM, Li WH (1987). The rate of synonymous substitution in enterobacterial genes is inversely related to codon usage bias.. Mol Biol Evol.

[16] Tamano K, Sano M, Yamane N, Terabayashi Y, Toda T (2008). Transcriptional regulation of genes on the non-syntenic blocks of *Aspergillus oryzae* and its functional relationship to solid-state cultivation.. Fungal Genet Biol.

[17] van de Peer Y (2004). Computational approaches to unveiling ancient genome duplications.. Nat Rev Genet.

[18] Simillion C, Vandepoele K, Van Montagu MC, Zabeau M, van de Peer Y (2002). The hidden duplication past of *Arabidopsis thaliana*.. Proc Natl Acad Sci U S A.

[19] Dehal P, Boore JL (2005). Two rounds of whole genome duplication in the ancestral vertebrate.. PLoS Biol.

[20] Aury JM, Jaillon O, Duret L, Noel B, Jubin C (2006). Global trends of whole-genome duplications revealed by the ciliate *Paramecium tetraurelia*.. Nature.

[21] Jaillon O, Aury JM, Noel B, Policriti A, Clepet C (2007). The grapevine genome sequence suggests ancestral hexaploidization in major angiosperm phyla.. Nature.

[22] Paterson AH, Bowers JE, Burow MD, Draye X, Elsik CG (2000). Comparative genomics of plant chromosomes.. Plant Cell.

[23] Kamper J, Kahmann R, Bolker M, Ma LJ, Brefort T (2006). Insights from the genome of the biotrophic fungal plant pathogen *Ustilago maydis*.. Nature.

[24] Geiser DM, Pitt JI, Taylor JW (1998). Cryptic speciation and recombination in the aflatoxin-producing fungus *Aspergillus flavus*.. Proc Natl Acad Sci U S A.

[25] Lee JM, Sonnhammer EL (2003). Genomic gene clustering analysis of pathways in eukaryotes.. Genome Res.

[26] Cai JJ, Woo PC, Lau SK, Smith DK, Yuen KY (2006). Accelerated evolutionary rate may be responsible for the emergence of lineage-specific genes in ascomycota.. J Mol Evol.

[27] Nishida H (2006). Detection and characterization of fungal-specific proteins in *Saccharomyces cerevisiae*.. Biosci Biotechnol Biochem.

[28] Lynch M, Conery JS (2003). The origins of genome complexity.. Science.

[29] Hall C, Brachat S, Dietrich FS (2005). Contribution of horizontal gene transfer to the evolution of *Saccharomyces cerevisiae*.. Eukaryot Cell.

[30] Nixon JE, Wang A, Field J, Morrison HG, McArthur AG (2002). Evidence for lateral transfer of genes encoding ferredoxins, nitroreductases, NADH oxidase, and alcohol dehydrogenase 3 from anaerobic prokaryotes to *Giardia lamblia* and *Entamoeba histolytica*.. Eukaryot Cell.

[31] Garcia-Vallve S, Romeu A, Palau J (2000). Horizontal gene transfer of glycosyl hydrolases of the rumen fungi.. Mol Biol Evol.

[32] Ricard G, McEwan NR, Dutilh BE, Jouany JP, Macheboeuf D (2006). Horizontal gene transfer from bacteria to rumen ciliates indicates adaptation to their anaerobic, carbohydrates-rich environment.. BMC Genomics.

[33] Andersson JO, Sjogren AM, Davis LA, Embley TM, Roger AJ (2003). Phylogenetic analyses of diplomonad genes reveal frequent lateral gene transfers affecting eukaryotes.. Curr Biol.

[34] Frigaard NU, Martinez A, Mincer TJ, DeLong EF (2006). Proteorhodopsin lateral gene transfer between marine planktonic Bacteria and Archaea.. Nature.

[35] Yang Z (2007). PAML 4: phylogenetic analysis by maximum likelihood.. Mol Biol Evol.

[36] Byrne KP, Wolfe KH (2005). The Yeast Gene Order Browser: combining curated homology and syntenic context reveals gene fate in polyploid species.. Genome Res.

[37] Thompson JD, Higgins DG, Gibson TJ (1994). CLUSTAL W: improving the sensitivity of progressive multiple sequence alignment through sequence weighting, position-specific gap penalties and weight matrix choice.. Nucleic Acids Res.

[38] Castresana J (2000). Selection of conserved blocks from multiple alignments for their use in phylogenetic analysis.. Mol Biol Evol.

[39] Guindon S, Gascuel O (2003). A simple, fast, and accurate algorithm to estimate large phylogenies by maximum likelihood.. Syst Biol.

[40] Yang Z (1997). PAML: a program package for phylogenetic analysis by maximum likelihood.. Comput Appl Biosci.

