# Supplementary material for: Elusive Origins of the Extra Genes in Aspergillus oryzae
Source: PLoS One. 2008 Aug 22;3(8):e3036. doi: 10.1371/journal.pone.0003036 (PMC2515630; doi:10.1371/journal.pone.0003036)
Supplement: Table S1 — Names and putative functions of A. oryzae gene pairs showing Topologies A, B or C. (0.03 MB DOC) [file pone.0003036.s001.doc]

**Table S1.** Names and putative functions of *A. oryzae* gene pairs showing Topologies A, B or C.

| AO1 | AO2 | Protein domains present |
| --- | --- | --- |
|  | | |
| Topology A | | |
| AO090701000472 | AO090103000225 | Isopenicillin N synthase and related dioxygenases family |
| AO090120000414 | AO090010000135 | Iron transporter |
| AO090003000721 | AO090009000136 | Homoserine dehydrogenase, NAD binding domain |
| AO090001000581 | AO090010000132 | Serine/threonine kinase TIP30/CC3. Predicted nucleoside-diphosphate-sugar epimerase |
| AO090102000393 | AO090003000572 | Fatty acyl-CoA elongation enzyme |
| AO090009000593 | AO090103000299 | Metal-dependent amidase/aminoacylase/carboxypeptidase |
| AO090012000848 | AO090009000109 | Haloacid dehalogenase-like hydrolase |
| AO090023000127 | AO090012000890 | Dihydroxy-acid dehydratase |
| AO090003000571 | AO090166000076 | Acetohydroxyacid synthase (AHAS) subfamily |
| AO090206000073 | AO090011000052 | Protease-associated (PA) domain |
| AO090011000374 | AO090003000277 | Short chain dehydrogenase |
| AO090102000131 | AO090012000011 | Conserved protein of unknown function |
|  |  |  |
| Topology B | | |
| AO090102000148 | AO090010000406 | Mitochondrial carrier protein |
| AO090102000626 | AO090113000140 | Mitochondrial carrier protein |
| AO090113000163 | AO090026000246 | X-Pro dipeptidyl-peptidase (S15 family) |
| AO090003000972 | AO090023000534 | Protease-associated (PA) domain |
| AO090012000452 | AO090012000771 | Conserved protein of unknown function |
| AO090038000292 | AO090012000674 | Dehydrogenase |
| AO090701000122 | AO090012000618 | Phosphodiesterase / nucleotide, GPI anchor synthesis protein |
| AO090001000454 | AO090120000159 | Mitochondrial carrier protein |
| AO090020000590 | AO090701000520 | 2-Nitropropane dioxygenase |
|  |  |  |
| Topology C | | |
| AO090010000480 | AO090124000011 | Actin depolymerisation factor/cofilin |
| AO090701000373 | AO090308000002 | Zn-dependent alcohol dehydrogenase |
| AO090026000284 | AO090023000874 | Tryptophan synthase, beta chain |
| AO090005001147 | AO090701000660 | Putative NADP-dependent oxidoreductase |
| AO090026000661 | AO090166000064 | Heavy metal exporter HMT1, ABC superfamily |
| AO090120000229 | AO090010000496 | Histidine kinase-like ATPase, HAMP domain |
| AO090102000109 | AO090012000014 | Unknown |
| AO090701000582 | AO090103000289 | Serine racemase |
| AO090003000922 | AO090124000052 | Ca2+/H+ antiporter VCX1 and related proteins |
| AO090206000105 | AO090010000720 | Ribonuclease T2 |
| AO090003000627 | AO090001000179 | RTA1 like protein |
| AO090011000825 | AO090138000089 | Mitochondrial endonuclease |
| AO090701000555 | AO090103000285 | Enoyl-CoA isomerase |
| AO090102000343 | AO090011000734 | Na+/K+ transporter |
