# Supplementary material for: Elusive Origins of the Extra Genes in Aspergillus oryzae
Source: PLoS One. 2008 Aug 22;3(8):e3036. doi: 10.1371/journal.pone.0003036 (PMC2515630; doi:10.1371/journal.pone.0003036)
Supplement: Figure S1 — Trees classified as Types A, B and C in each Aspergillus species. Trees were constructed using PHYML as described in Methods. In each tree, the sequences identified as AO1 and AO2 (for duplications A. oryzae), AN1 and AN2 (for duplications in A. nidulans), or AFU1 and AFU2 (for duplications in A. fumigatus) are labeled. NCBI identifier (GI) numbers for each sequence are shown. (0.93 MB PDF) [file pone.0003036.s002.pdf]

Figure S1. Trees classified as Types A, B and C in each Aspergillus species. Trees were constructed using PHYML as described in Methods. In each tree, the sequences identified as *AO1* and *AO2* (for duplications in *A. oryzae*), *AN1* and *AN2* (for duplications in *A. nidulans*), or *AFU1* and *AFU2* (for duplications in *A. fumigatus*) are labeled. NCBI identifier (GI) numbers for each sequence are shown.

Trees classified as Topology A in *Aspergillus oryzae* (12).

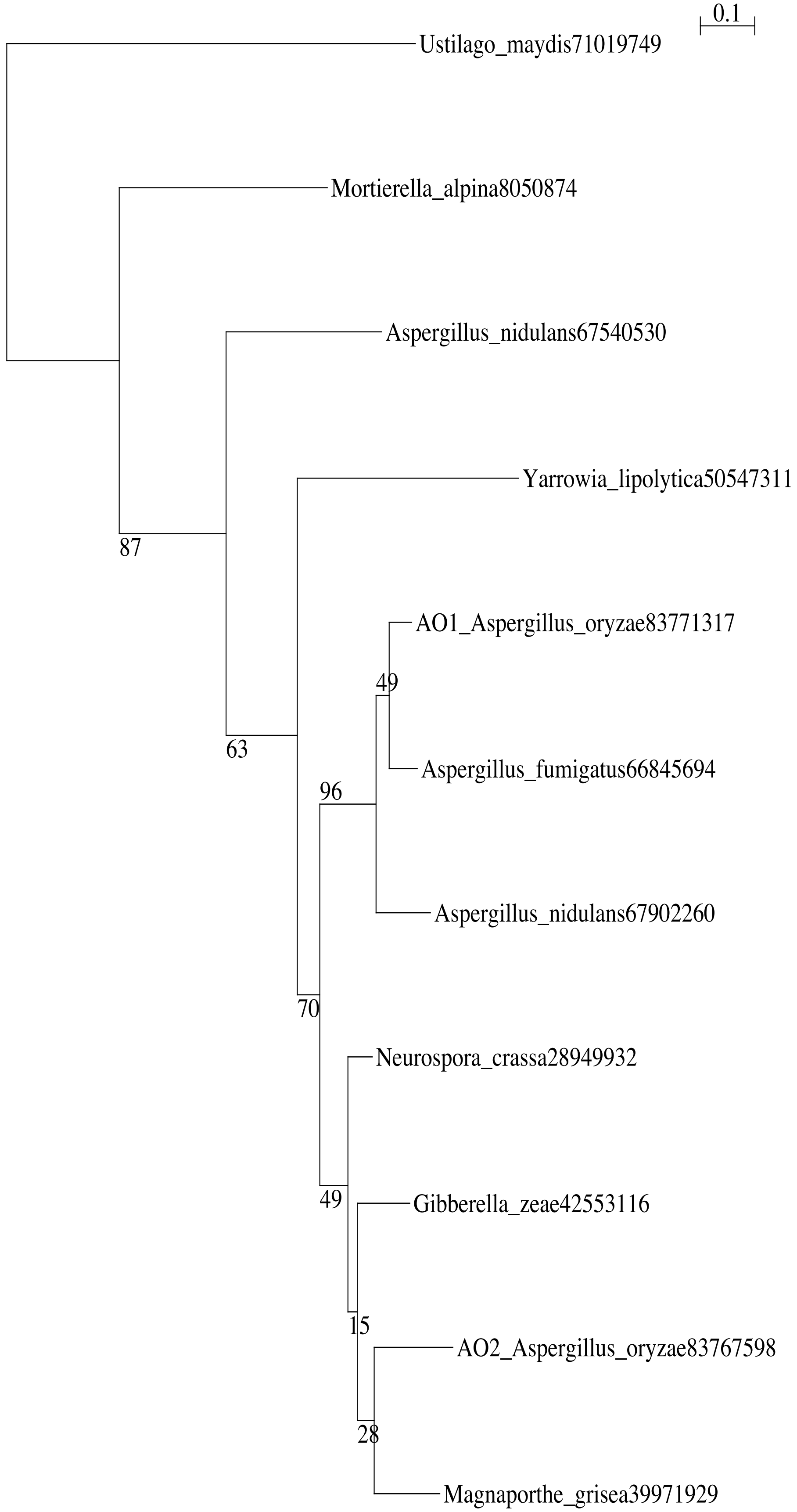
0.1
Ustilago_maydis71019749
Mortierella_alpina8050874
Aspergillus_nidulans67540530
Yarrowia_lipolytica50547311
AO1_Aspergillus_oryzae83771317
Aspergillus_fumigatus66845694
Aspergillus_nidulans67902260
Neurospora_crassa28949932
Gibberella_zeae42553116
AO2_Aspergillus_oryzae83767598
Magnaporthe_grisea39971929
87
63
70
96
49
49
15
28

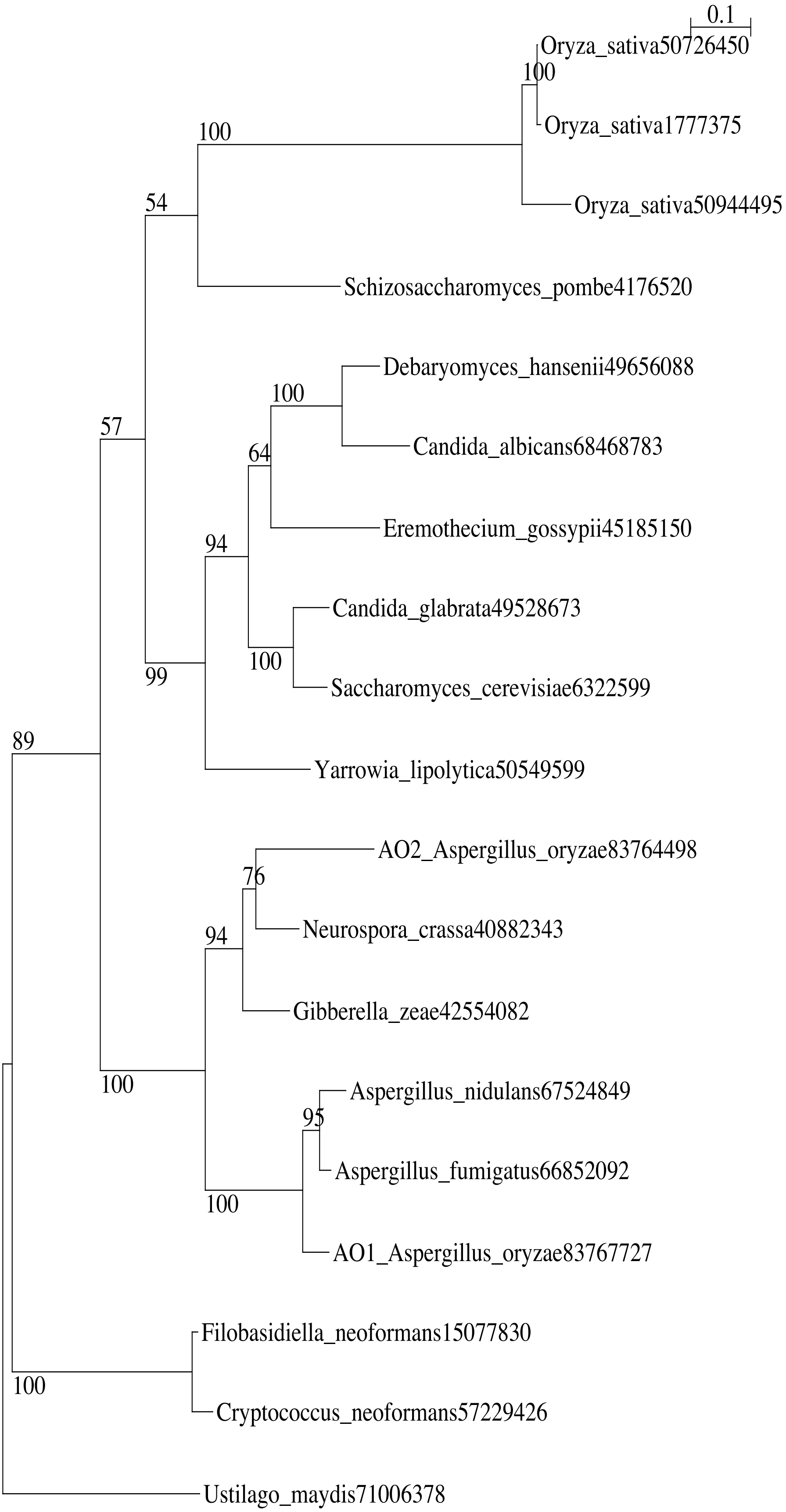
0.1
Oryza_sativa50726450
100
Oryza_sativa1777375
100
Oryza_sativa50944495
54
Schizosaccharomyces_pombe4176520
Debaryomyces_hansenii49656088
100
57
Candida_albicans68468783
64
Eremothecium_gossypii45185150
94
Candida_glabrata49528673
100
99
Saccharomyces_cerevisiae6322599
89
Yarrowia_lipolytica50549599
AO2_Aspergillus_oryzae83764498
76
Neurospora_crassa40882343
94
Gibberella_zeae42554082
100
Aspergillus_nidulans67524849
95
Aspergillus_fumigatus66852092
100
AO1_Aspergillus_oryzae83767727
Filobasidiella_neoformans15077830
100
Cryptococcus_neoformans57229426
Ustilago_maydis71006378

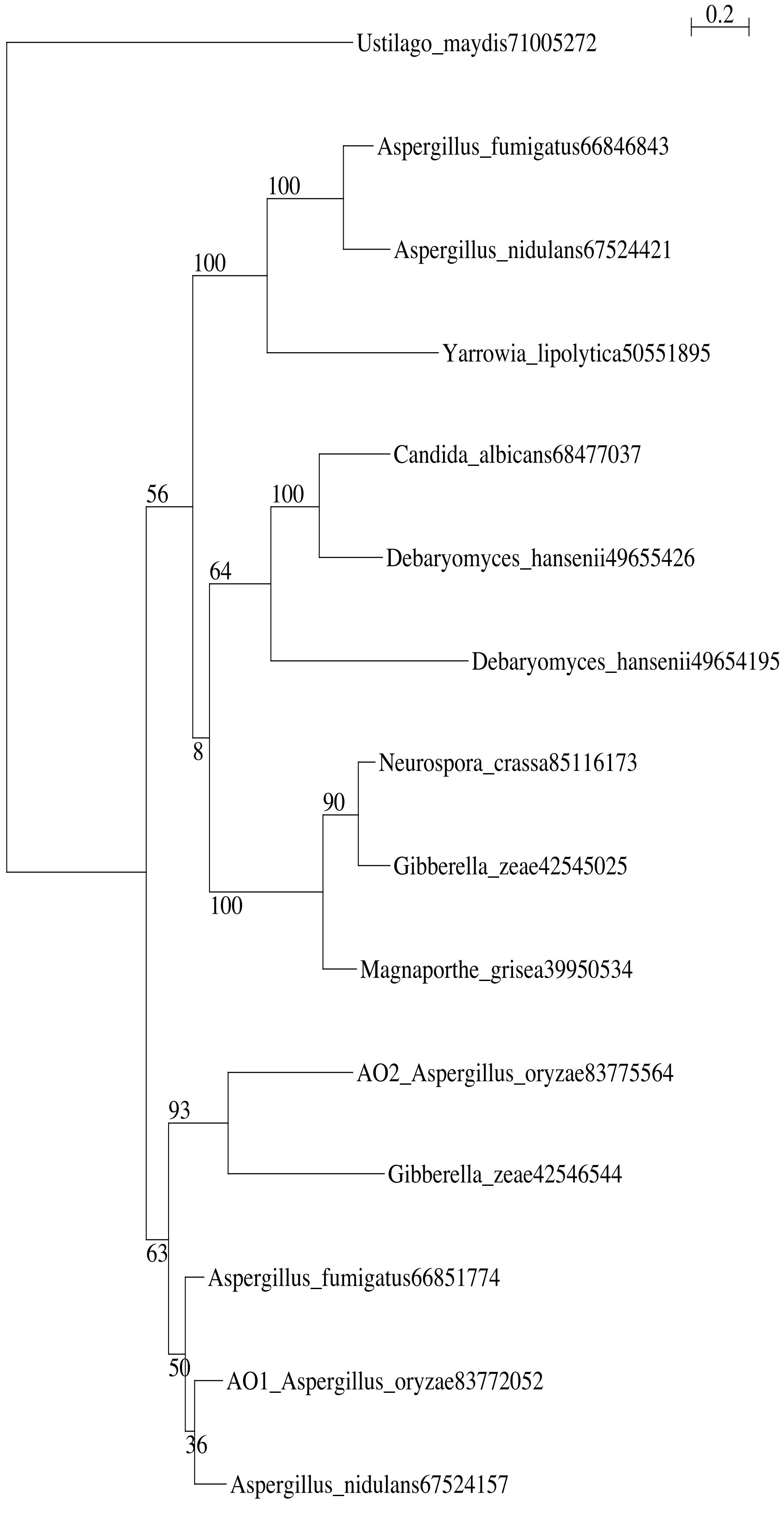
0.2
Ustilago_maydis71005272
Aspergillus_fumigatus66846843
Aspergillus_nidulans67524421
Yarrowia_lipolytica50551895
Candida_albicans68477037
Debaryomyces_hansenii49655426
Debaryomyces_hansenii49654195
Neurospora_crassa85116173
Gibberella_zeae42545025
Magnaporthe_grisea39950534
AO2_Aspergillus_oryzae83775564
Gibberella_zeae42546544
Aspergillus_fumigatus66851774
AO1_Aspergillus_oryzae83772052
Aspergillus_nidulans67524157
100
100
56
100
64
8
90
100
93
63
50
36

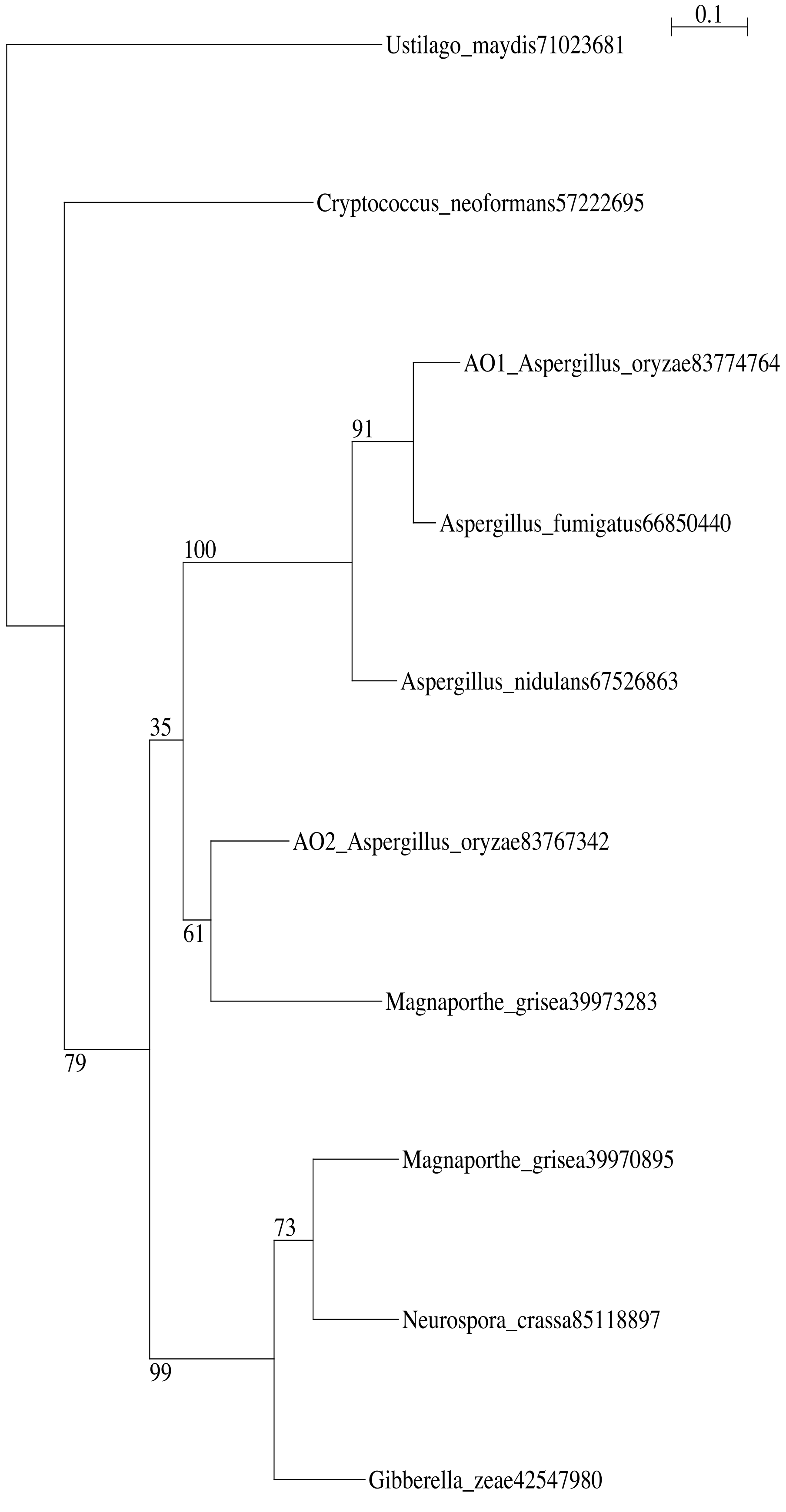
0.1
Ustilago_maydis71023681
Cryptococcus_neoformans57222695
AO1_Aspergillus_oryzae83774764
91
Aspergillus_fumigatus66850440
100
Aspergillus_nidulans67526863
35
AO2_Aspergillus_oryzae83767342
61
Magnaporthe_grisea39973283
79
Magnaporthe_grisea39970895
73
Neurospora_crassa85118897
99
Gibberella_zeae42547980

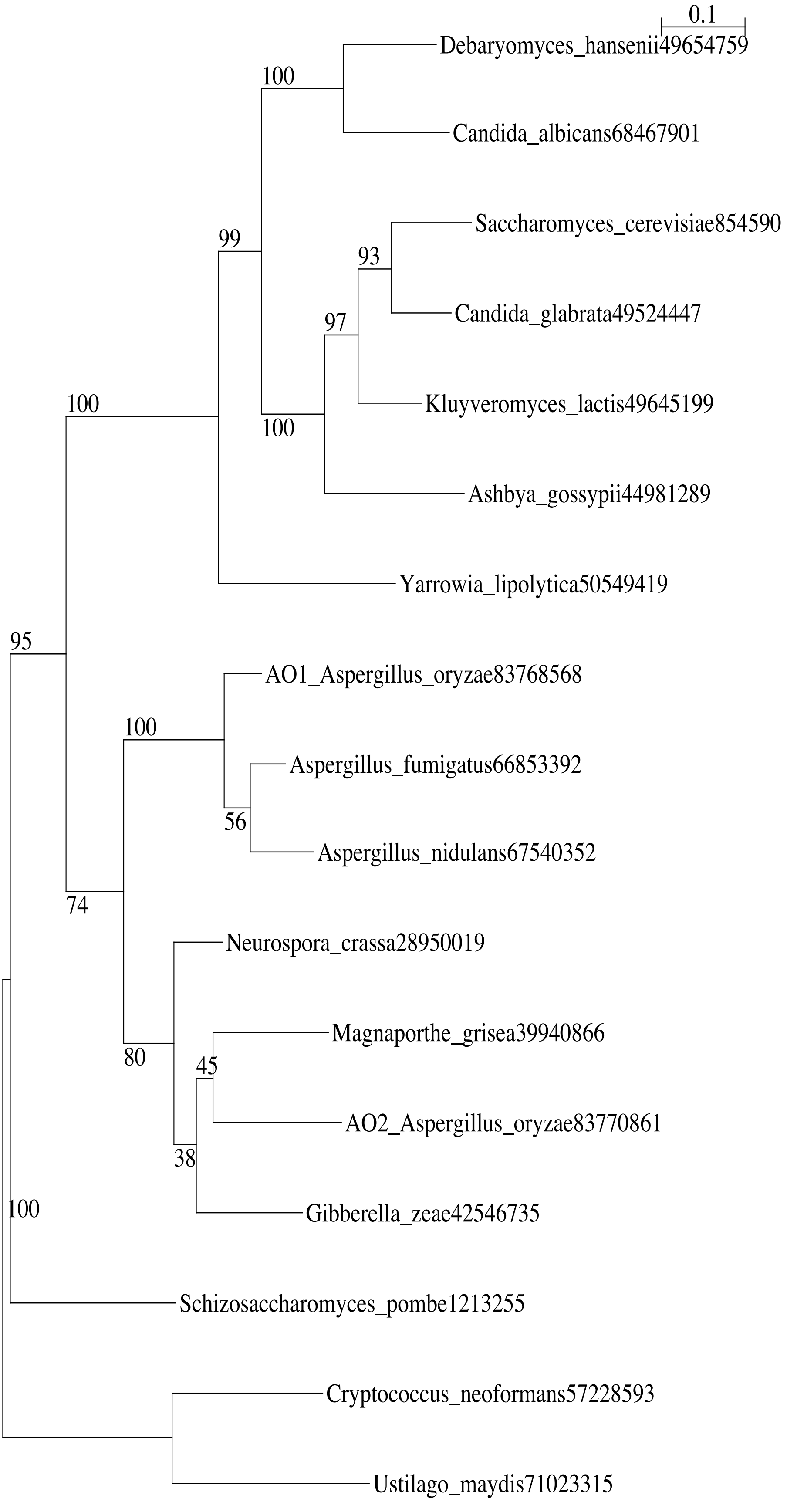
0.1
Debaryomyces_hansenii49654759
Candida_albicans68467901
Saccharomyces_cerevisiae854590
Candida_glabrata49524447
Kluyveromyces_lactis49645199
Ashbya_gossypii44981289
Yarrowia_lipolytica50549419
AO1_Aspergillus_oryzae83768568
Aspergillus_fumigatus66853392
Aspergillus_nidulans67540352
Neurospora_crassa28950019
Magnaporthe_grisea39940866
AO2_Aspergillus_oryzae83770861
Gibberella_zeae42546735
Schizosaccharomyces_pombe1213255
Cryptococcus_neoformans57228593
Ustilago_maydis71023315
100
99
93
97
100
100
95
100
56
74
80
45
38
100

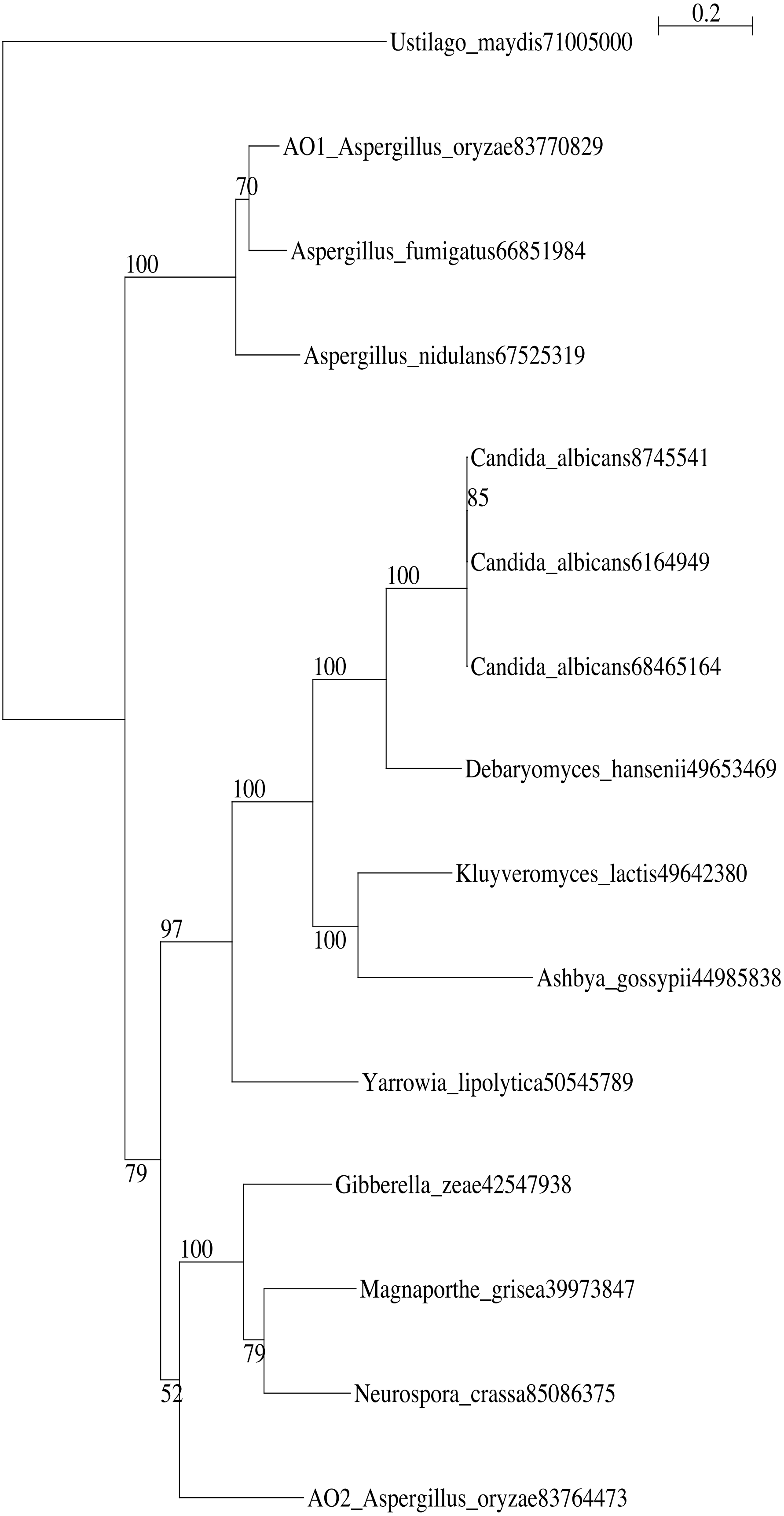

0.2
Ustilago_maydis71005000
AO1_Aspergillus_oryzae83770829
70
Aspergillus_fumigatus66851984
100
Aspergillus_nidulans67525319
Candida_albicans8745541
85
Candida_albicans6164949
100
Candida_albicans68465164
100
Debaryomyces_hansenii49653469
100
Kluyveromyces_lactis49642380
97
100
Ashbya_gossypii44985838
Yarrowia_lipolytica50545789
79
Gibberella_zeae42547938
100
Magnaporthe_grisea39973847
79
52
Neurospora_crassa85086375
AO2_Aspergillus_oryzae83764473

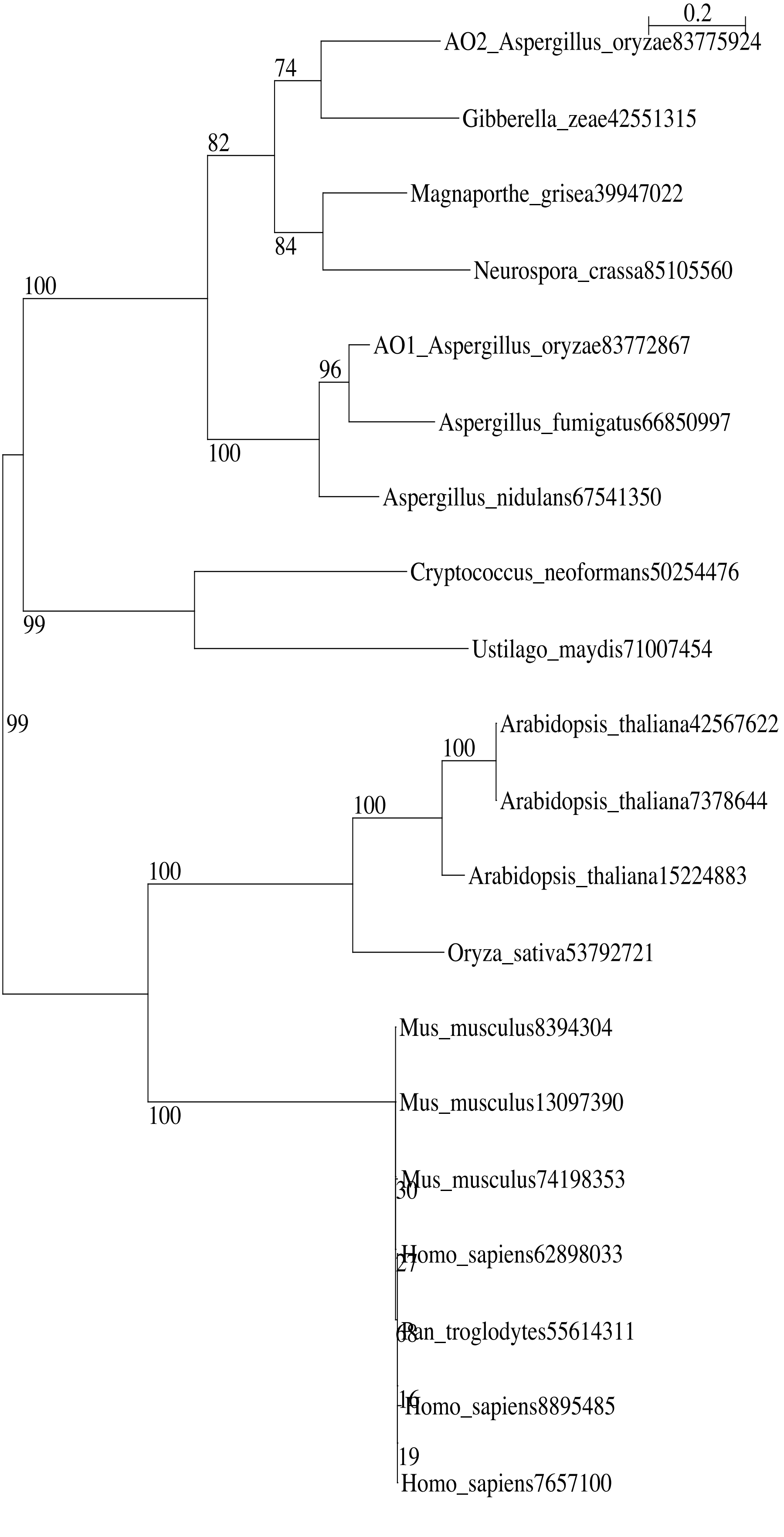
0.2
AO2_Aspergillus_oryzae83775924
Gibberella_zeae42551315
Magnaporthe_grisea39947022
Neurospora_crassa85105560
AO1_Aspergillus_oryzae83772867
Aspergillus_fumigatus66850997
Aspergillus_nidulans67541350
Cryptococcus_neoformans50254476
Ustilago_maydis71007454
Arabidopsis_thaliana42567622
Arabidopsis_thaliana7378644
Arabidopsis_thaliana15224883
Oryza_sativa53792721
Mus_musculus8394304
Mus_musculus13097390
Mus_musculus74198353
Homo_sapiens62898033
Pan_troglodytes55614311
Homo_sapiens8895485
Homo_sapiens7657100
74
82
84
100
96
100
99
99
100
100
100
100
30
27
68
16
19

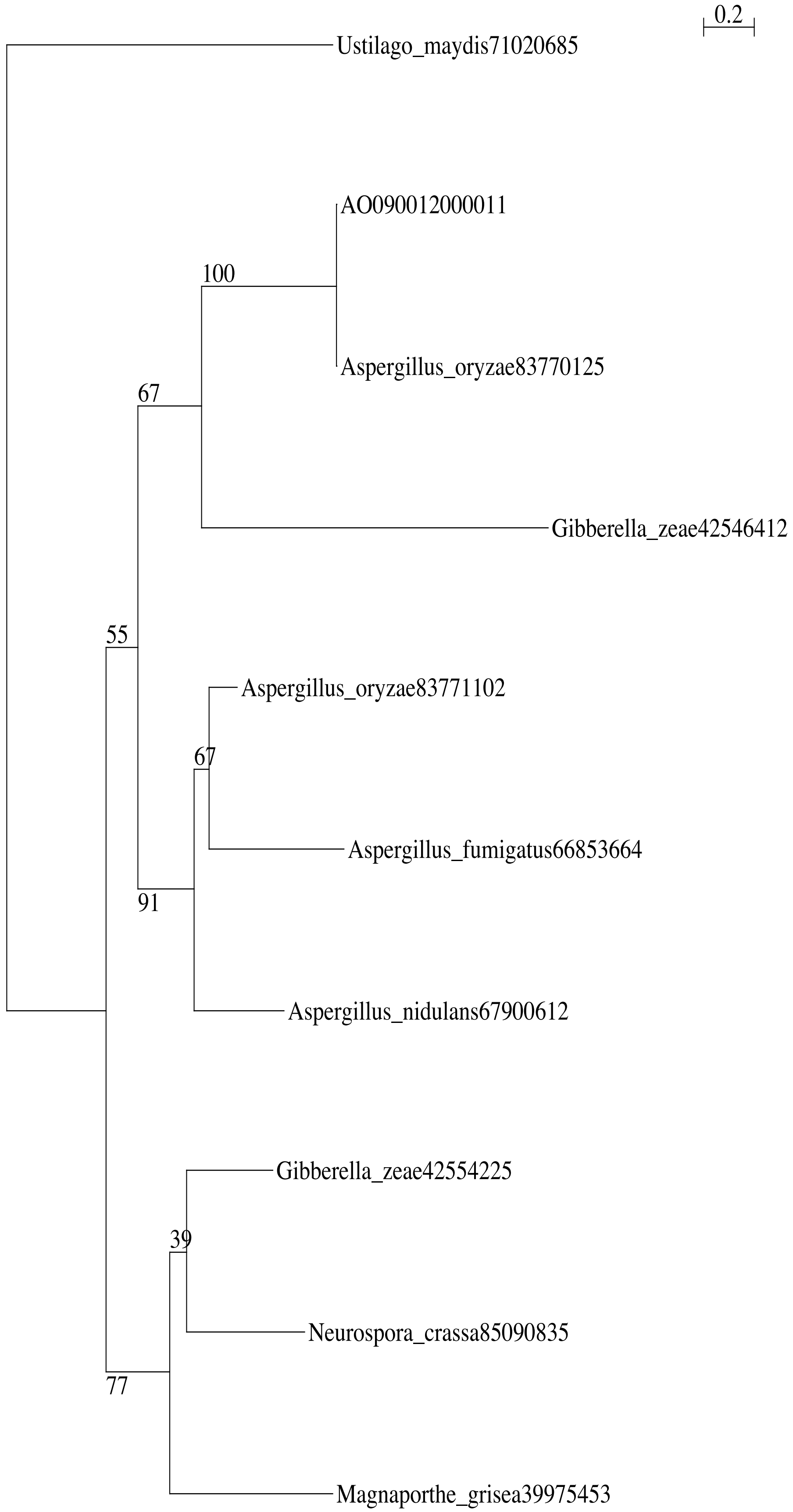

0.2
Ustilago_maydis71020685
AO090012000011
100
Aspergillus_oryzae83770125
67
Gibberella_zeae42546412
55
Aspergillus_oryzae83771102
67
Aspergillus_fumigatus66853664
91
Aspergillus_nidulans67900612
Gibberella_zeae42554225
39
Neurospora_crassa85090835
77
Magnaporthe_grisea39975453

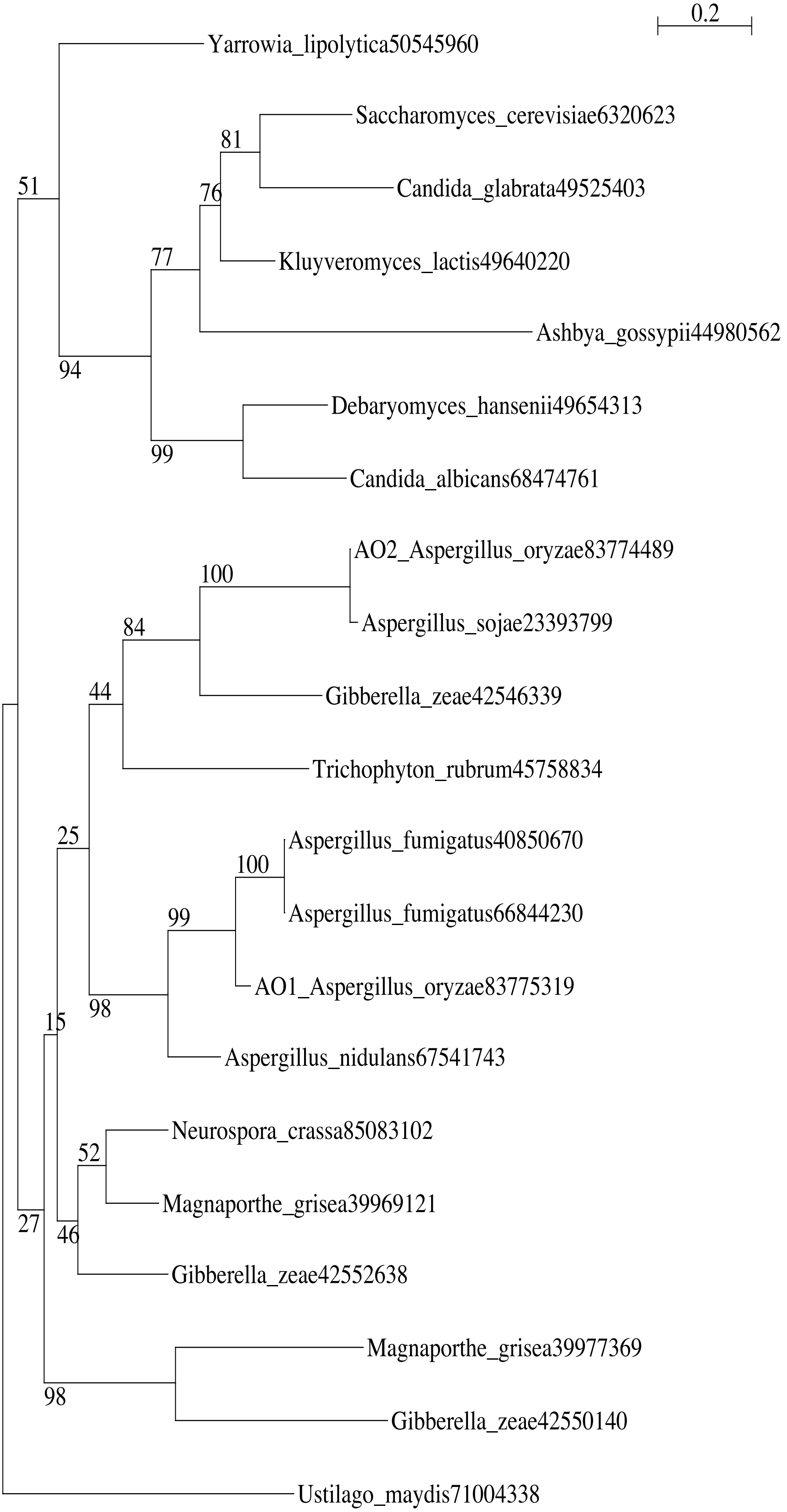
0.2
Yarrowia_lipolytica50545960
Saccharomyces_cerevisiae6320623
81
Candida_glabrata49525403
51
76
Kluyveromyces_lactis49640220
77
Ashbya_gossypii44980562
94
Debaryomyces_hansenii49654313
99
Candida_albicans68474761
AO2_Aspergillus_oryzae83774489
100
Aspergillus_sojae23393799
84
Gibberella_zeae42546339
44
Trichophyton_rubrum45758834
25
Aspergillus_fumigatus40850670
100
Aspergillus_fumigatus66844230
99
AO1_Aspergillus_oryzae83775319
98
15
Aspergillus_nidulans67541743
Neurospora_crassa85083102
52
Magnaporthe_grisea39969121
27
46
Gibberella_zeae42552638
Magnaporthe_grisea39977369
98
Gibberella_zeae42550140
Ustilago_maydis71004338

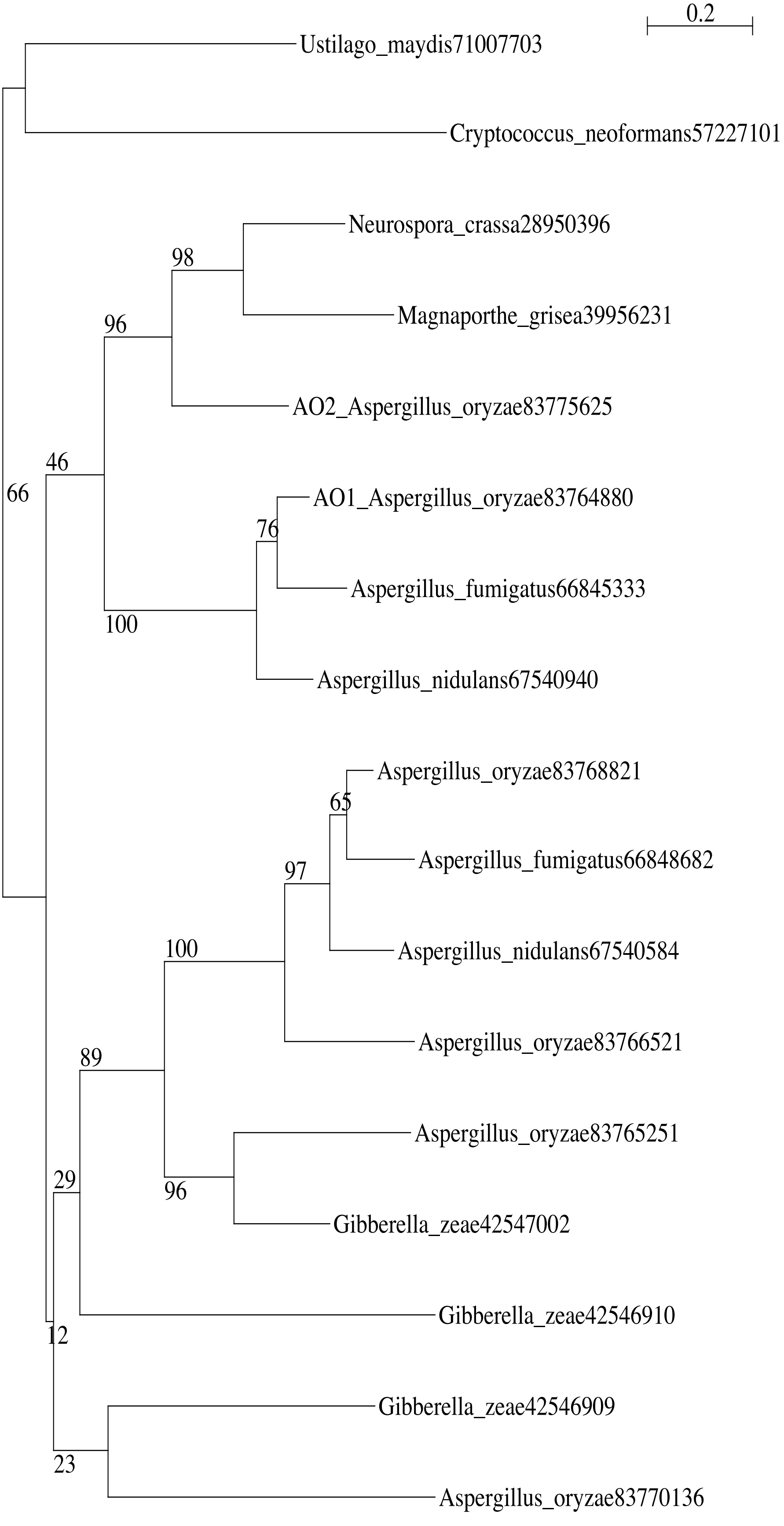
0.2
Ustilago_maydis71007703
Cryptococcus_neoformans57227101
Neurospora_crassa28950396
98
Magnaporthe_grisea39956231
96
AO2_Aspergillus_oryzae83775625
46
66
AO1_Aspergillus_oryzae83764880
76
Aspergillus_fumigatus66845333
100
Aspergillus_nidulans67540940
Aspergillus_oryzae83768821
65
Aspergillus_fumigatus66848682
97
Aspergillus_nidulans67540584
100
Aspergillus_oryzae83766521
89
Aspergillus_oryzae83765251
29
96
Gibberella_zeae42547002
Gibberella_zeae42546910
12
Gibberella_zeae42546909
23
Aspergillus_oryzae83770136

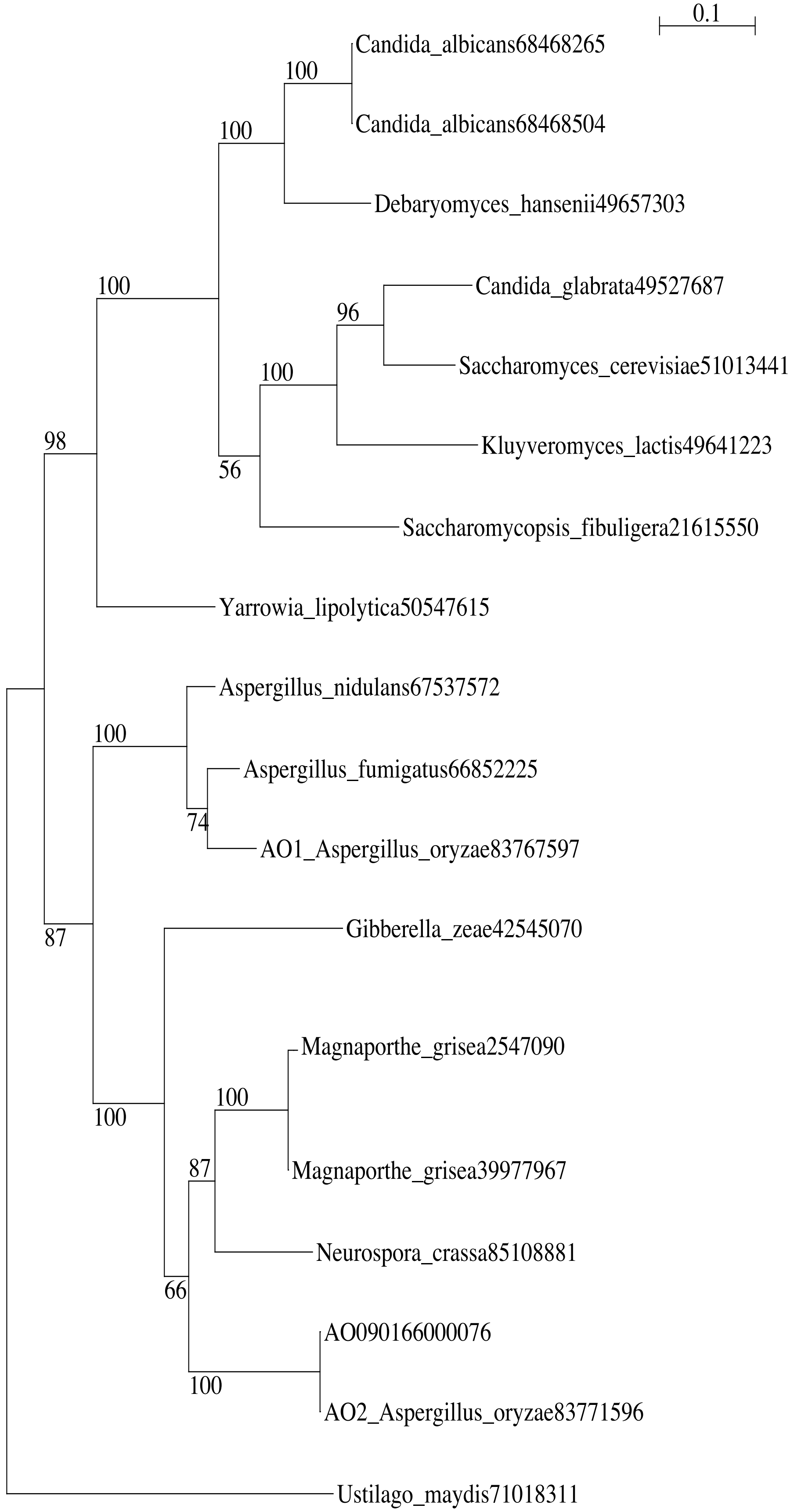
0.1
Candida_albicans68468265
Candida_albicans68468504
Debaryomyces_hansenii49657303
Candida_glabrata49527687
Saccharomyces_cerevisiae51013441
Kluyveromyces_lactis49641223
Saccharomycopsis_fibuligera21615550
Yarrowia_lipolytica50547615
Aspergillus_nidulans67537572
Aspergillus_fumigatus66852225
AO1_Aspergillus_oryzae83767597
Gibberella_zeae42545070
Magnaporthe_grisea2547090
Magnaporthe_grisea39977967
Neurospora_crassa85108881
AO090166000076
AO2_Aspergillus_oryzae83771596
Ustilago_maydis71018311
100
100
100
96
100
98
56
100
74
87
100
87
100
66
100

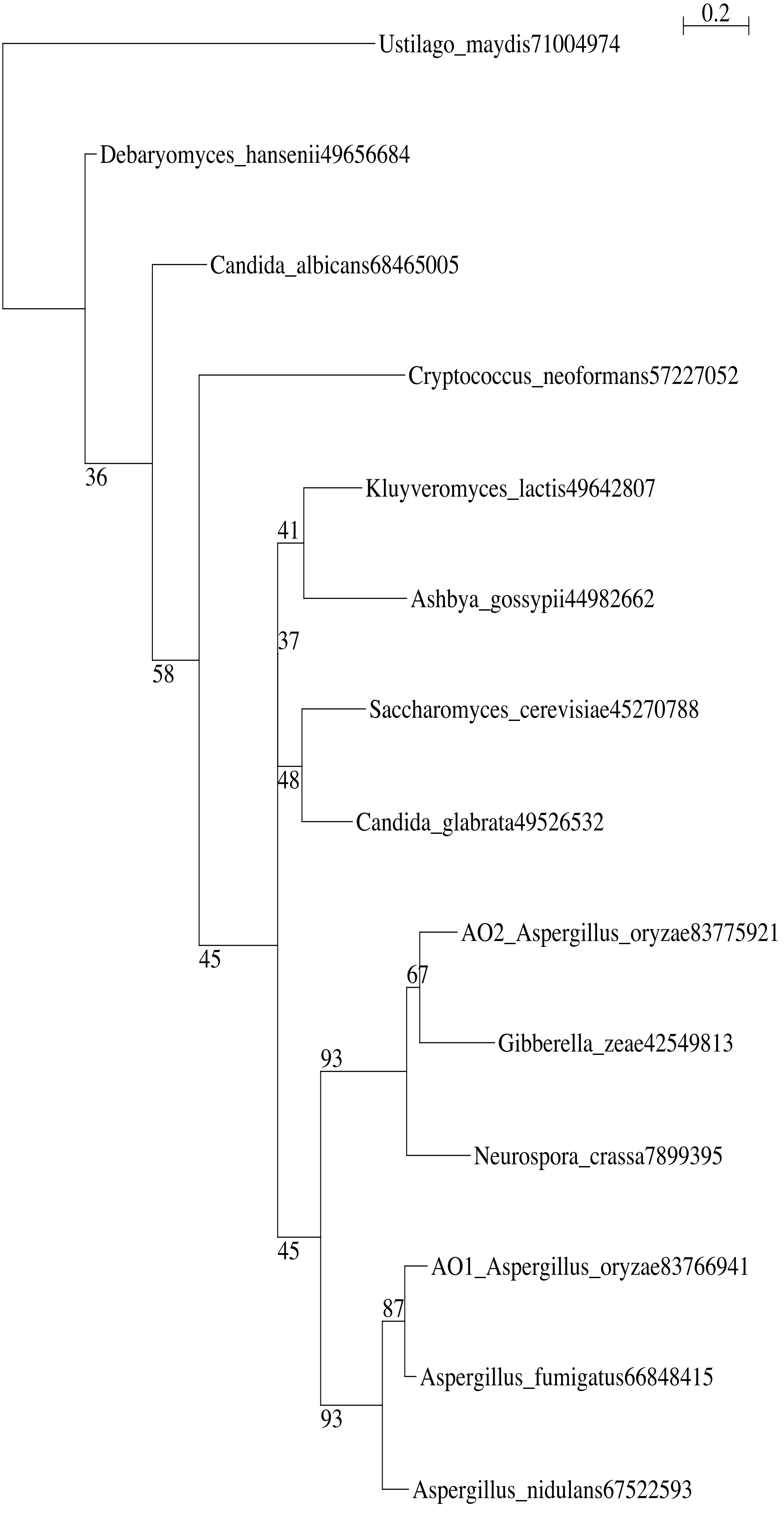
0.2
Ustilago_maydis71004974
Debaryomyces_hansenii49656684
Candida_albicans68465005
Cryptococcus_neoformans57227052
Kluyveromyces_lactis49642807
Ashbya_gossypii44982662
Saccharomyces_cerevisiae45270788
Candida_glabrata49526532
AO2_Aspergillus_oryzae83775921
Gibberella_zeae42549813
Neurospora_crassa7899395
AO1_Aspergillus_oryzae83766941
Aspergillus_fumigatus66848415
Aspergillus_nidulans67522593
36
58
45
41
37
48
45
67
93
87
93

Trees classified as Topology B in *Aspergillus oryzae* (9).

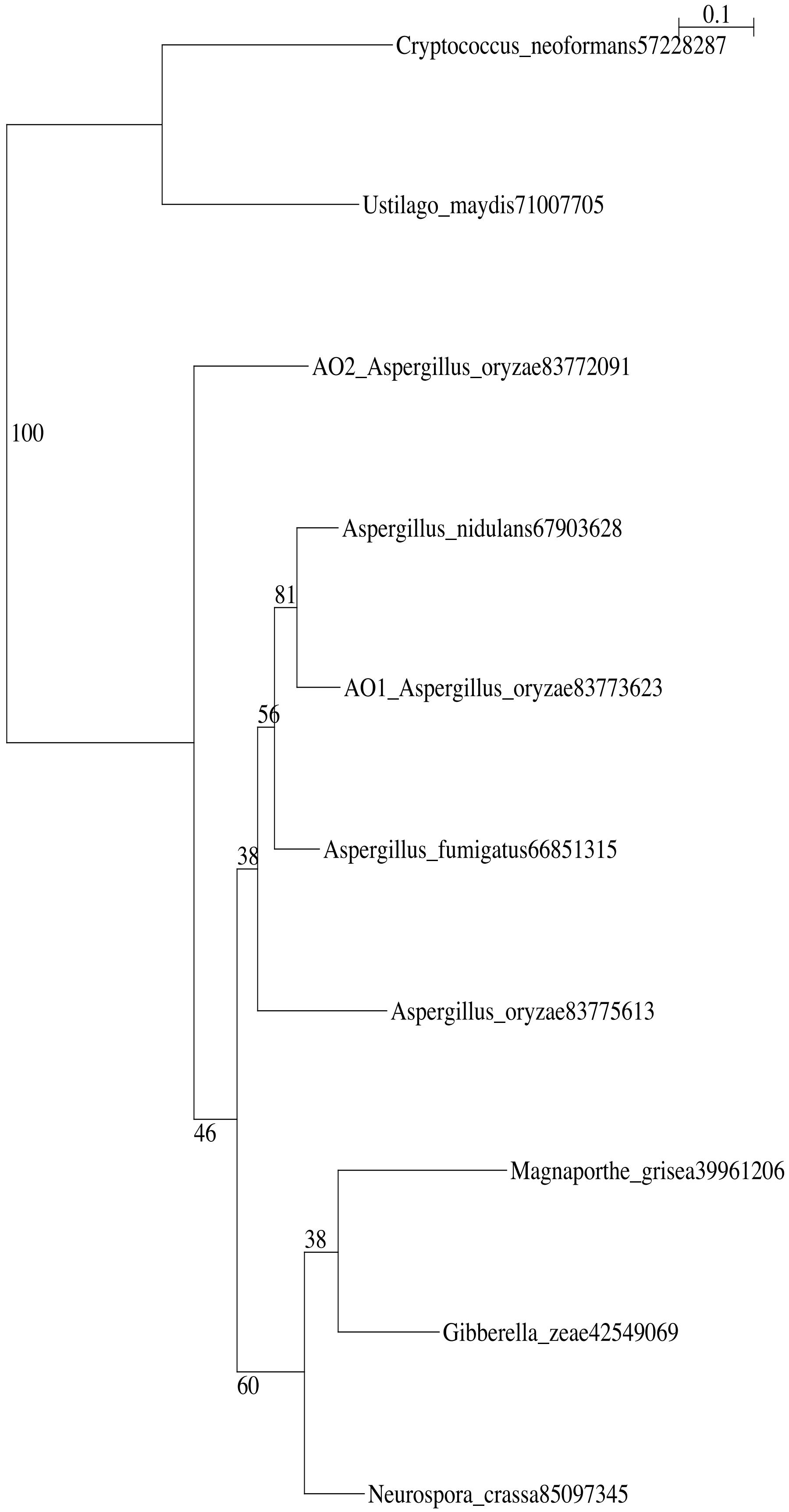
0.1
Cryptococcus_neoformans57228287
Ustilago_maydis71007705
100
AO2_Aspergillus_oryzae83772091
Aspergillus_nidulans67903628
81
AO1_Aspergillus_oryzae83773623
56
Aspergillus_fumigatus66851315
38
Aspergillus_oryzae83775613
46
Magnaporthe_grisea39961206
38
Gibberella_zeae42549069
60
Neurospora_crassa85097345

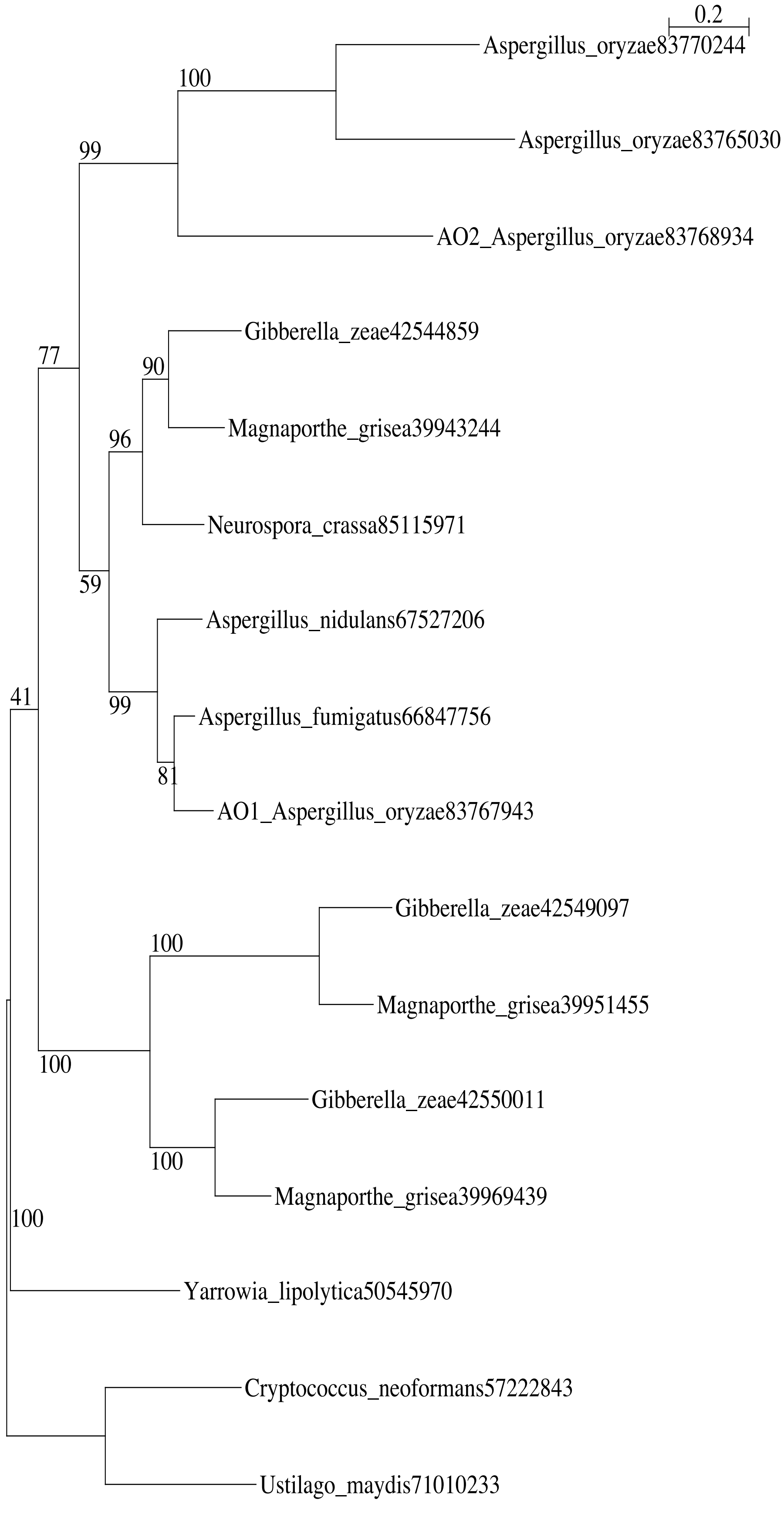
0.2
Aspergillus_oryzae83770244
Aspergillus_oryzae83765030
AO2_Aspergillus_oryzae83768934
Gibberella_zeae42544859
Magnaporthe_grisea39943244
Neurospora_crassa85115971
Aspergillus_nidulans67527206
Aspergillus_fumigatus66847756
AO1_Aspergillus_oryzae83767943
Gibberella_zeae42549097
Magnaporthe_grisea39951455
Gibberella_zeae42550011
Magnaporthe_grisea39969439
Yarrowia_lipolytica50545970
Cryptococcus_neoformans57222843
Ustilago_maydis71010233
100
99
77
90
96
59
99
81
41
100
100
100
100

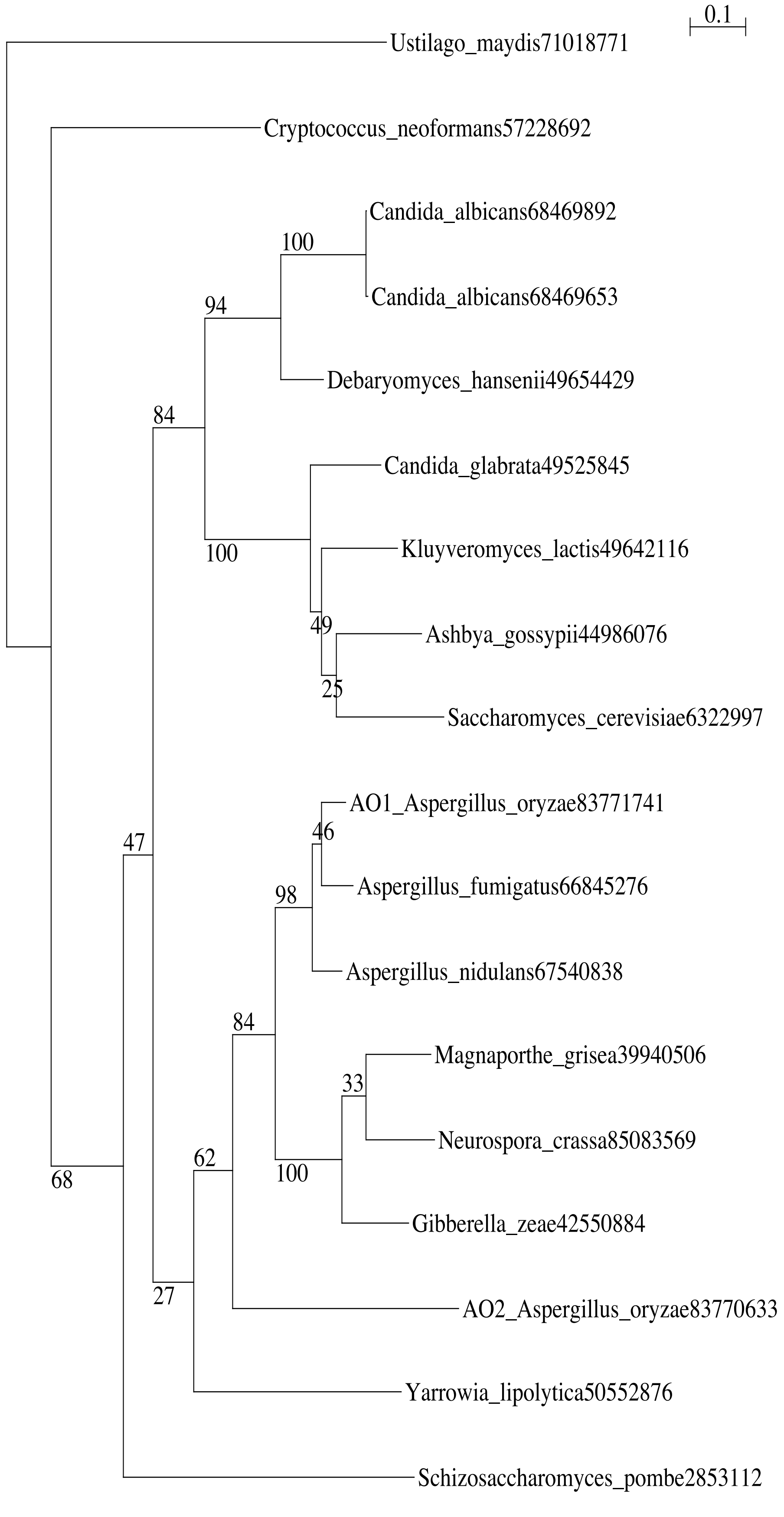
0.1
Ustilago_maydis71018771
Cryptococcus_neoformans57228692
Candida_albicans68469892
Candida_albicans68469653
Debaryomyces_hansenii49654429
Candida_glabrata49525845
Kluyveromyces_lactis49642116
Ashbya_gossypii44986076
Saccharomyces_cerevisiae6322997
AO1_Aspergillus_oryzae83771741
Aspergillus_fumigatus66845276
Aspergillus_nidulans67540838
Magnaporthe_grisea39940506
Neurospora_crassa85083569
Gibberella_zeae42550884
AO2_Aspergillus_oryzae83770633
Yarrowia_lipolytica50552876
Schizosaccharomyces_pombe2853112
100
94
84
100
49
25
46
98
84
33
100
62
47
68
27

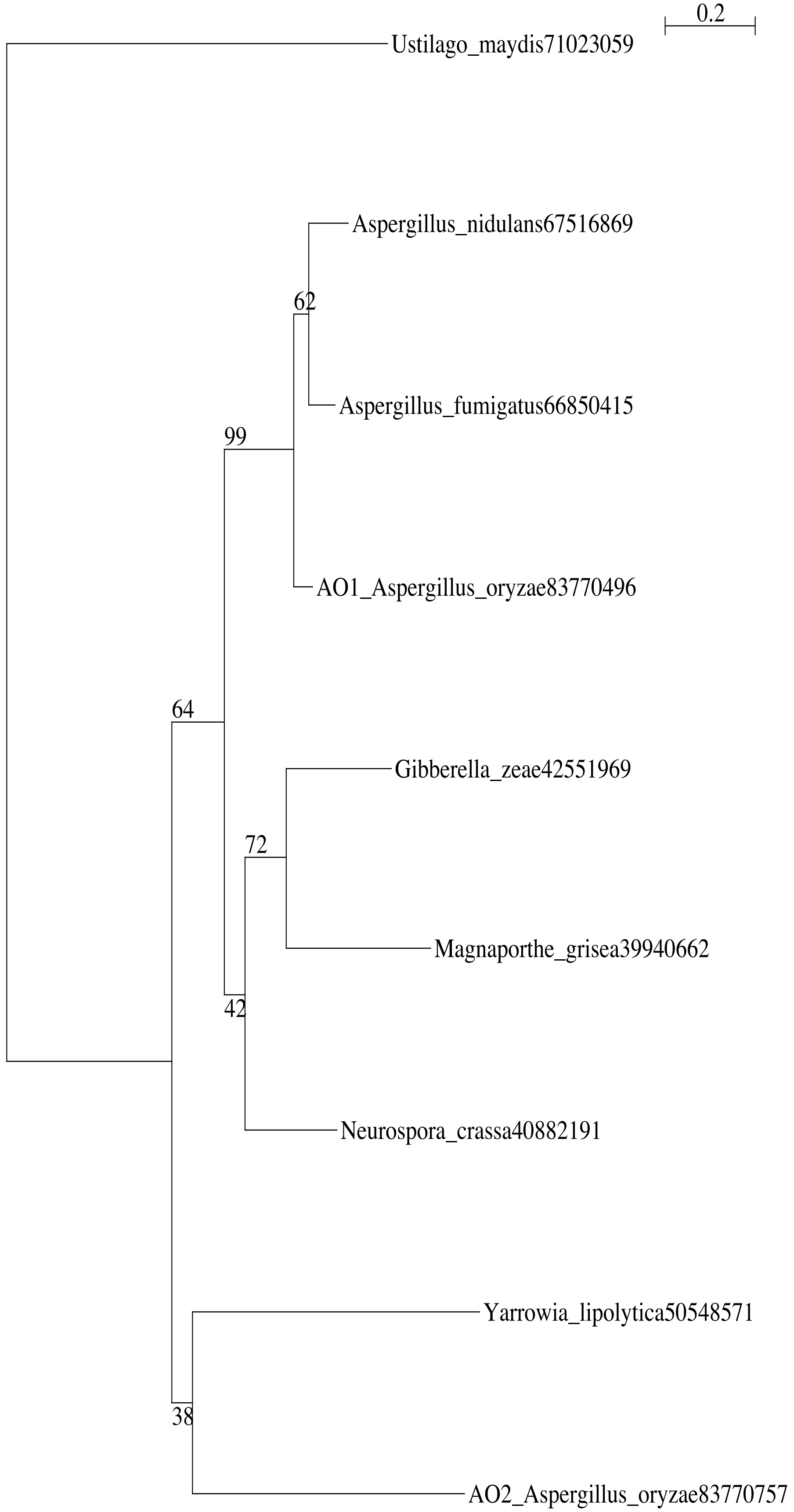
0.2
Ustilago_maydis71023059
Aspergillus_nidulans67516869
62
Aspergillus_fumigatus66850415
99
AO1_Aspergillus_oryzae83770496
64
Gibberella_zeae42551969
72
Magnaporthe_grisea39940662
42
Neurospora_crassa40882191
Yarrowia_lipolytica50548571
38
AO2_Aspergillus_oryzae83770757

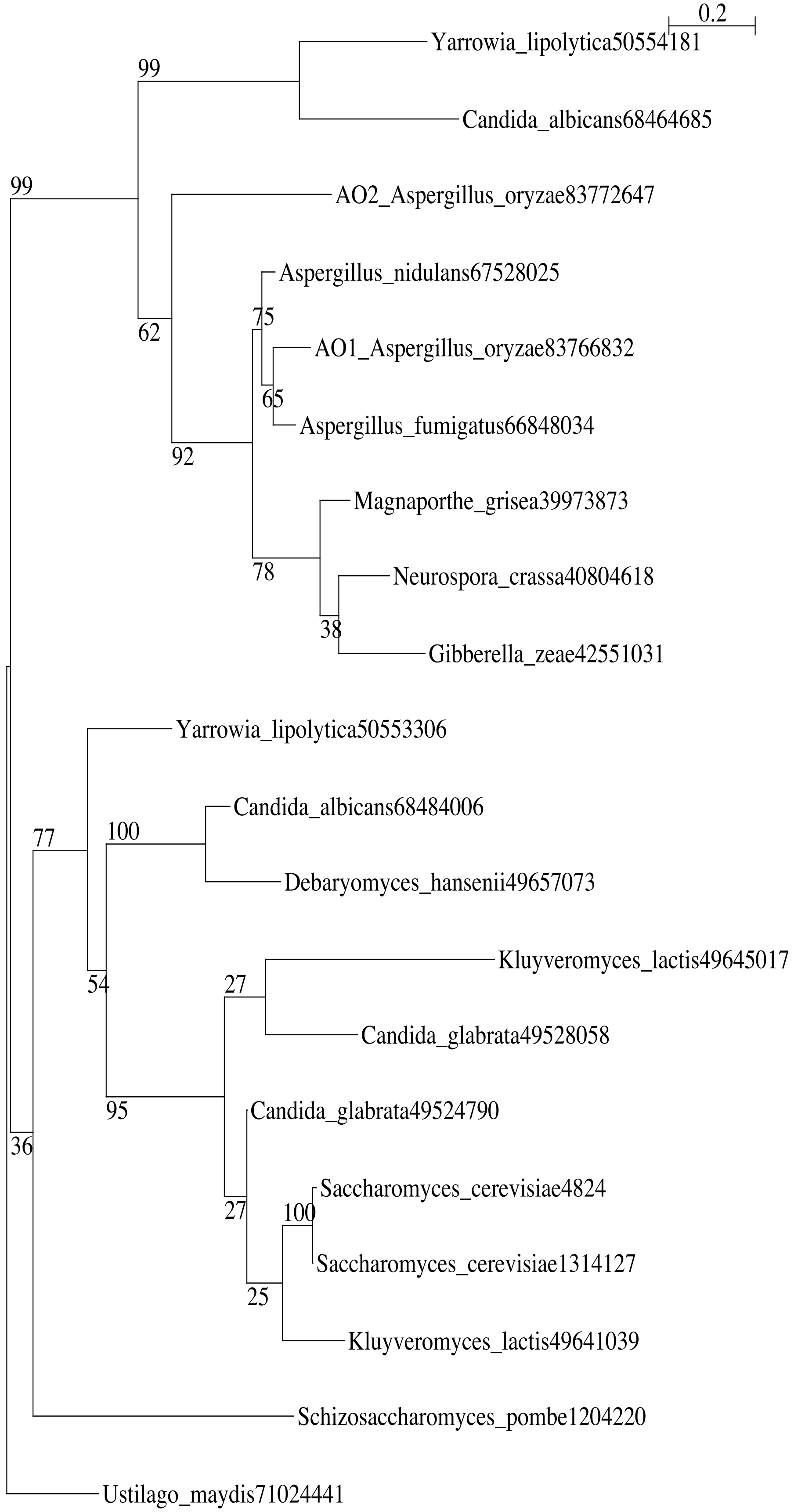
0.2
Yarrowia_lipolytica50554181
Candida_albicans68464685
AO2_Aspergillus_oryzae83772647
Aspergillus_nidulans67528025
AO1_Aspergillus_oryzae83766832
Aspergillus_fumigatus66848034
Magnaporthe_grisea39973873
Neurospora_crassa40804618
Gibberella_zeae42551031
Yarrowia_lipolytica50553306
Candida_albicans68484006
Debaryomyces_hansenii49657073
Kluyveromyces_lactis49645017
Candida_glabrata49528058
Candida_glabrata49524790
Saccharomyces_cerevisiae4824
Saccharomyces_cerevisiae1314127
Kluyveromyces_lactis49641039
Schizosaccharomyces_pombe1204220
Ustilago_maydis71024441
99
99
62
75
65
92
78
38
77
100
54
27
95
36
27
100
25

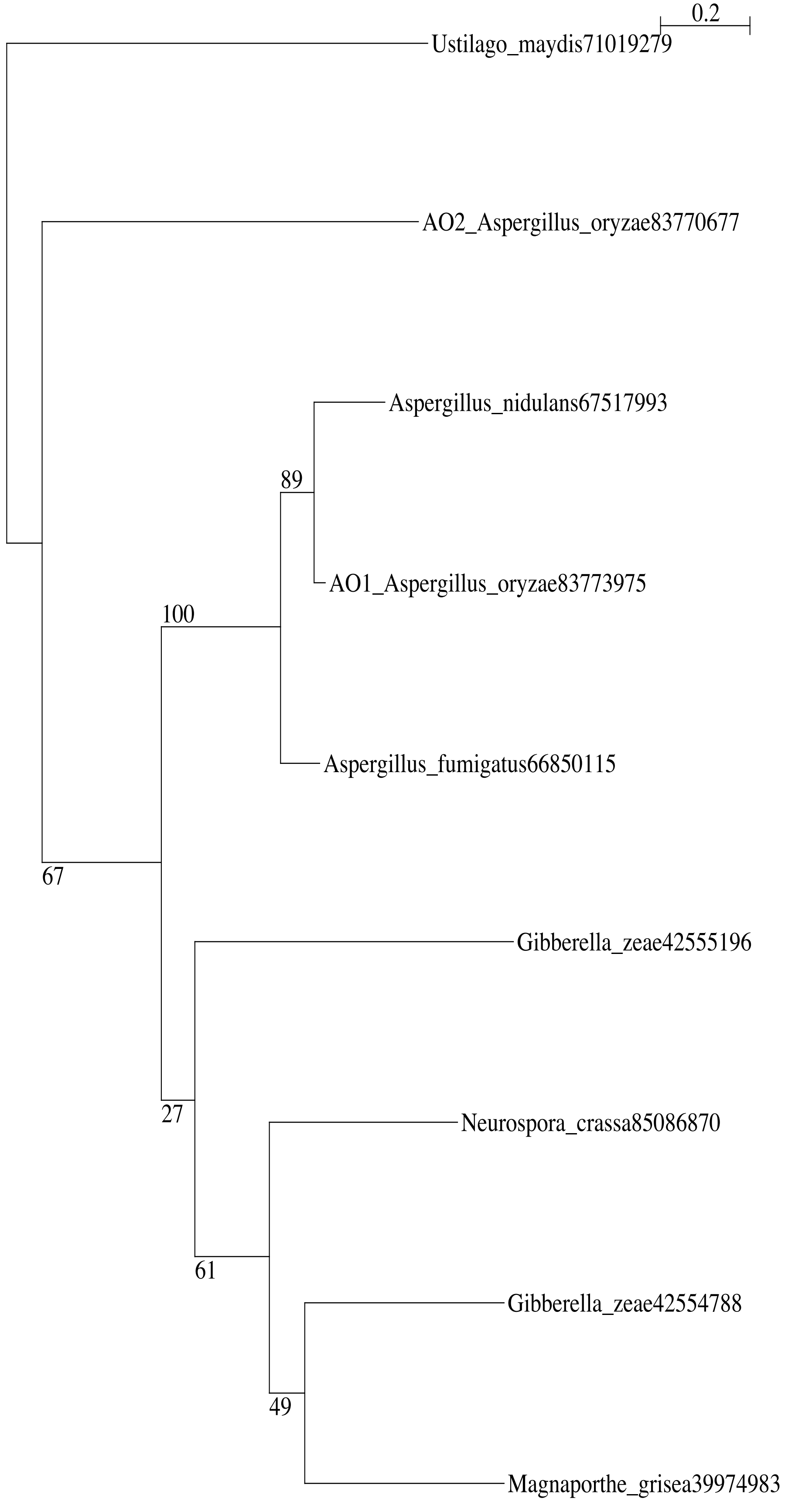
0.2
Ustilago_maydis71019279
AO2_Aspergillus_oryzae83770677
Aspergillus_nidulans67517993
89
AO1_Aspergillus_oryzae83773975
100
Aspergillus_fumigatus66850115
67
Gibberella_zeae42555196
27
Neurospora_crassa85086870
61
Gibberella_zeae42554788
49
Magnaporthe_grisea39974983

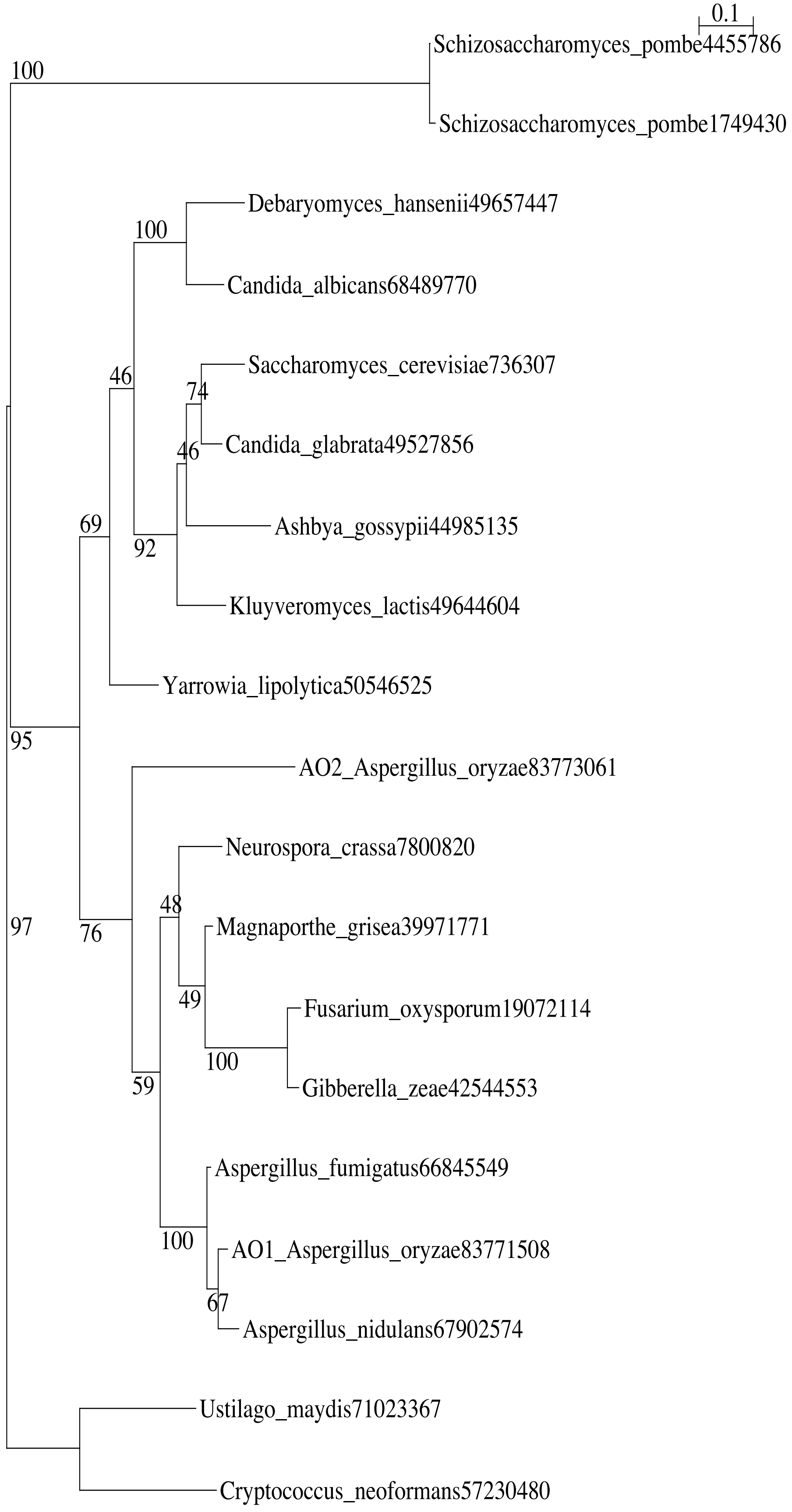
0.1
Schizosaccharomyces_pombe4455786
Schizosaccharomyces_pombe1749430
Debaryomyces_hansenii49657447
Candida_albicans68489770
Saccharomyces_cerevisiae736307
Candida_glabrata49527856
Ashbya_gossypii44985135
Kluyveromyces_lactis49644604
Yarrowia_lipolytica50546525
AO2_Aspergillus_oryzae83773061
Neurospora_crassa7800820
Magnaporthe_grisea39971771
Fusarium_oxysporum19072114
Gibberella_zeae42544553
Aspergillus_fumigatus66845549
AO1_Aspergillus_oryzae83771508
Aspergillus_nidulans67902574
Ustilago_maydis71023367
Cryptococcus_neoformans57230480
100
100
46
74
46
69
92
95
76
48
49
100
97
59
100
67

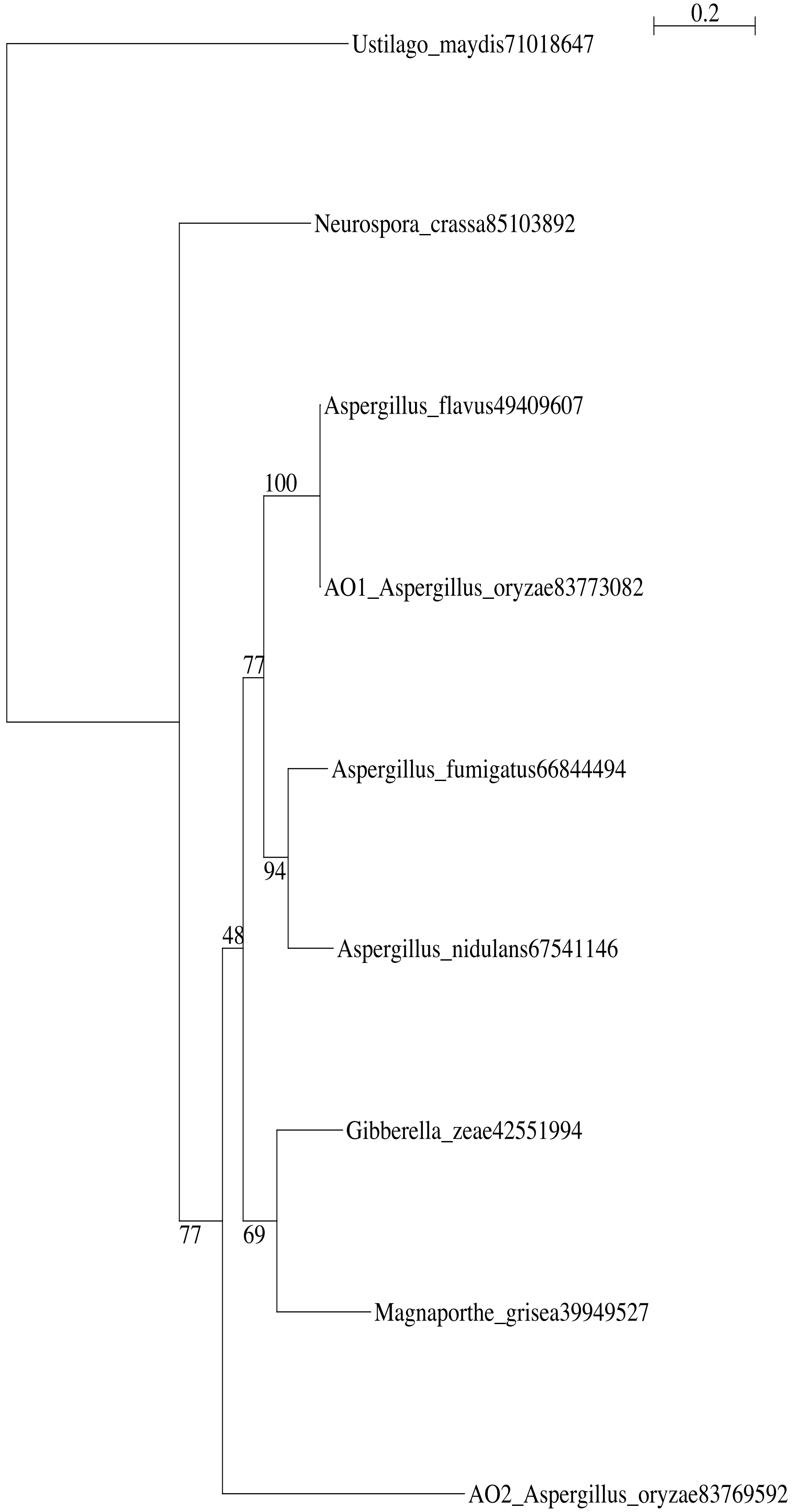
0.2
Ustilago_maydis71018647
Neurospora_crassa85103892
Aspergillus_flavus49409607
100
AO1_Aspergillus_oryzae83773082
77
Aspergillus_fumigatus66844494
94
48
Aspergillus_nidulans67541146
Gibberella_zeae42551994
77
69
Magnaporthe_grisea39949527
AO2_Aspergillus_oryzae83769592

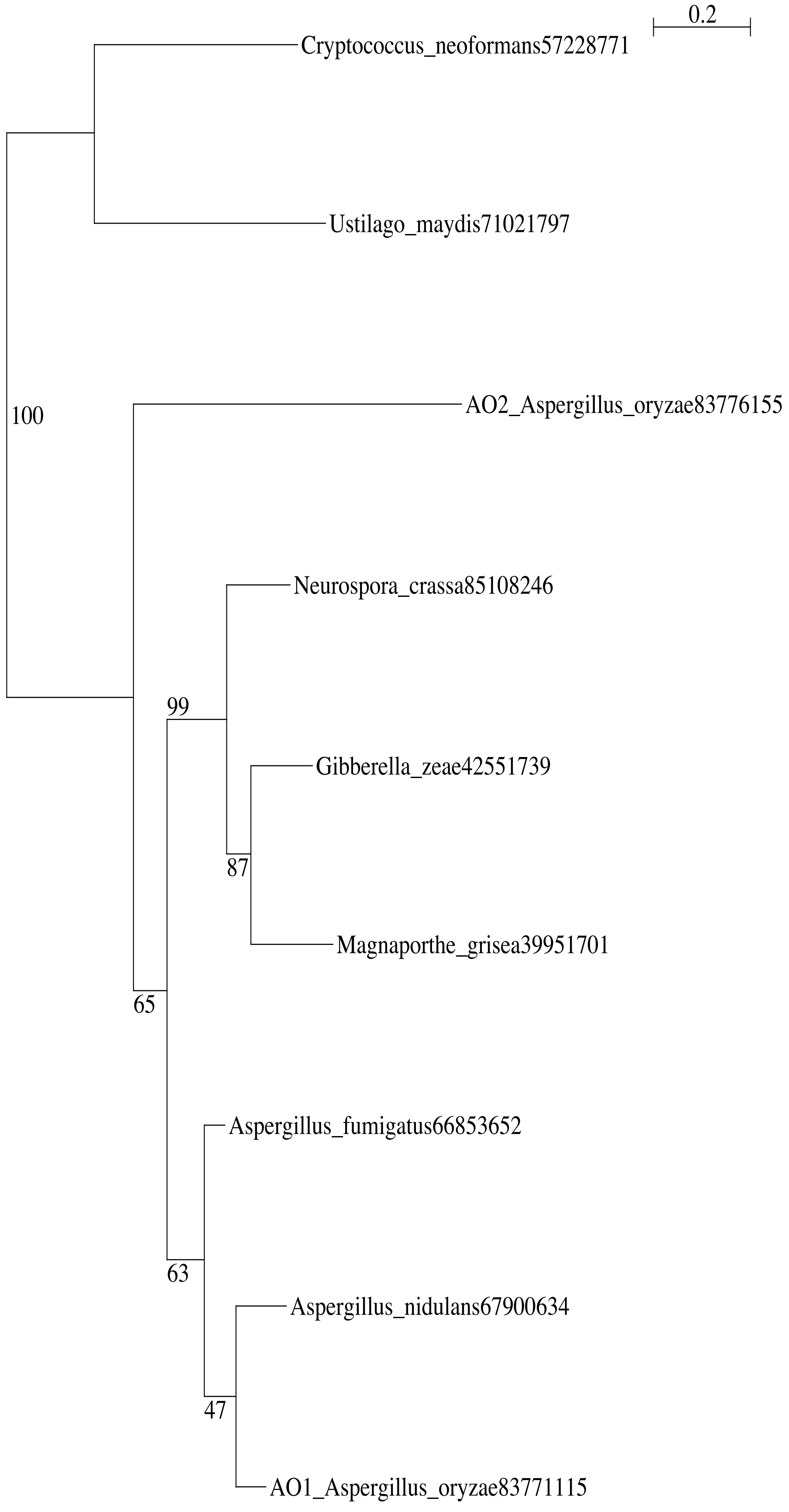
0.2
Cryptococcus_neoformans57228771
Ustilago_maydis71021797
100
AO2_Aspergillus_oryzae83776155
Neurospora_crassa85108246
99
Gibberella_zeae42551739
87
Magnaporthe_grisea39951701
65
Aspergillus_fumigatus66853652
63
Aspergillus_nidulans67900634
47
AO1_Aspergillus_oryzae83771115

Trees classified as Topology C in *Aspergillus oryzae* (14).

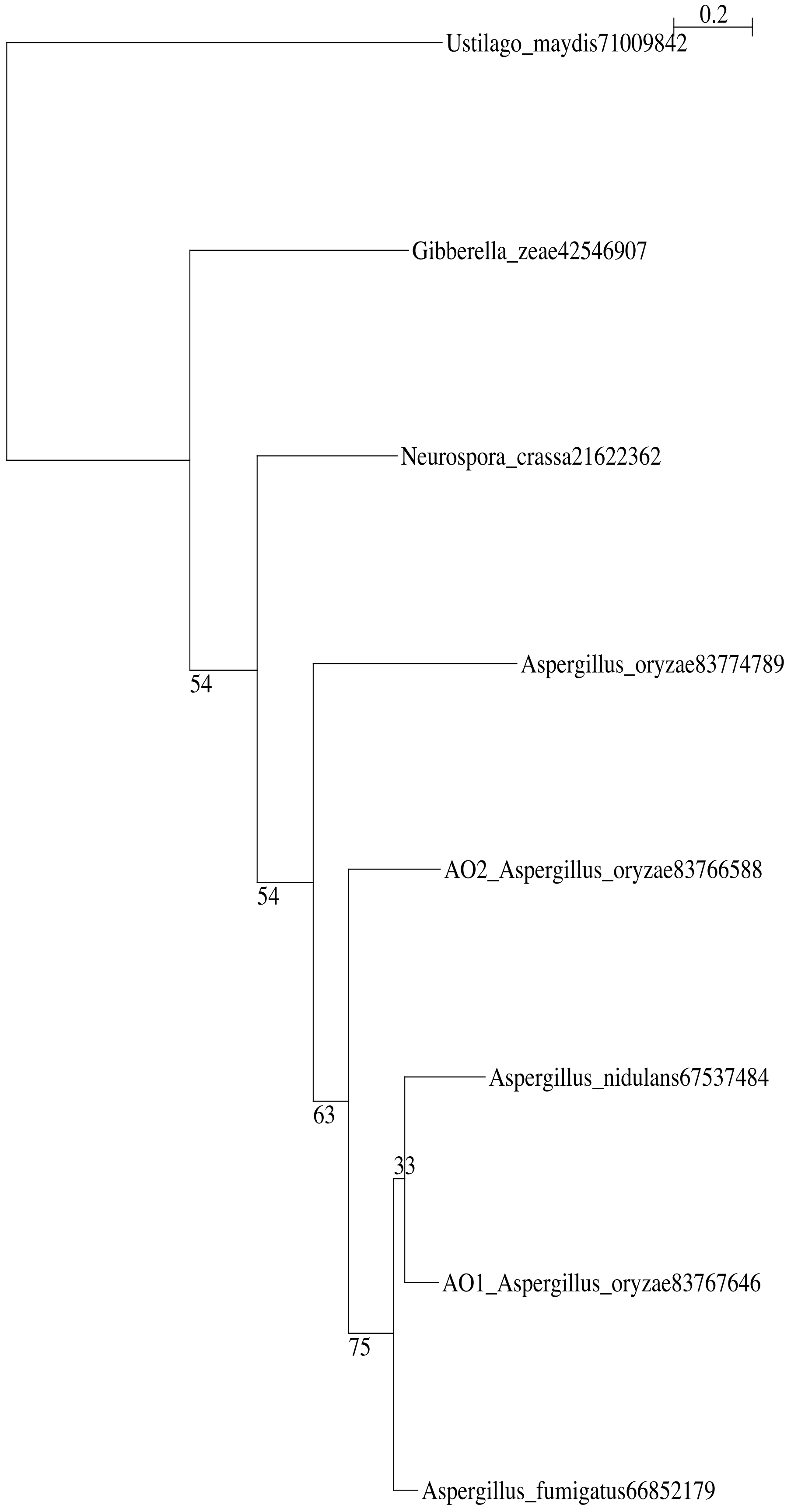
0.2
Ustilago_maydis71009842
Gibberella_zeae42546907
Neurospora_crassa21622362
54
Aspergillus_oryzae83774789
54
AO2_Aspergillus_oryzae83766588
63
Aspergillus_nidulans67537484
33
AO1_Aspergillus_oryzae83767646
75
Aspergillus_fumigatus66852179

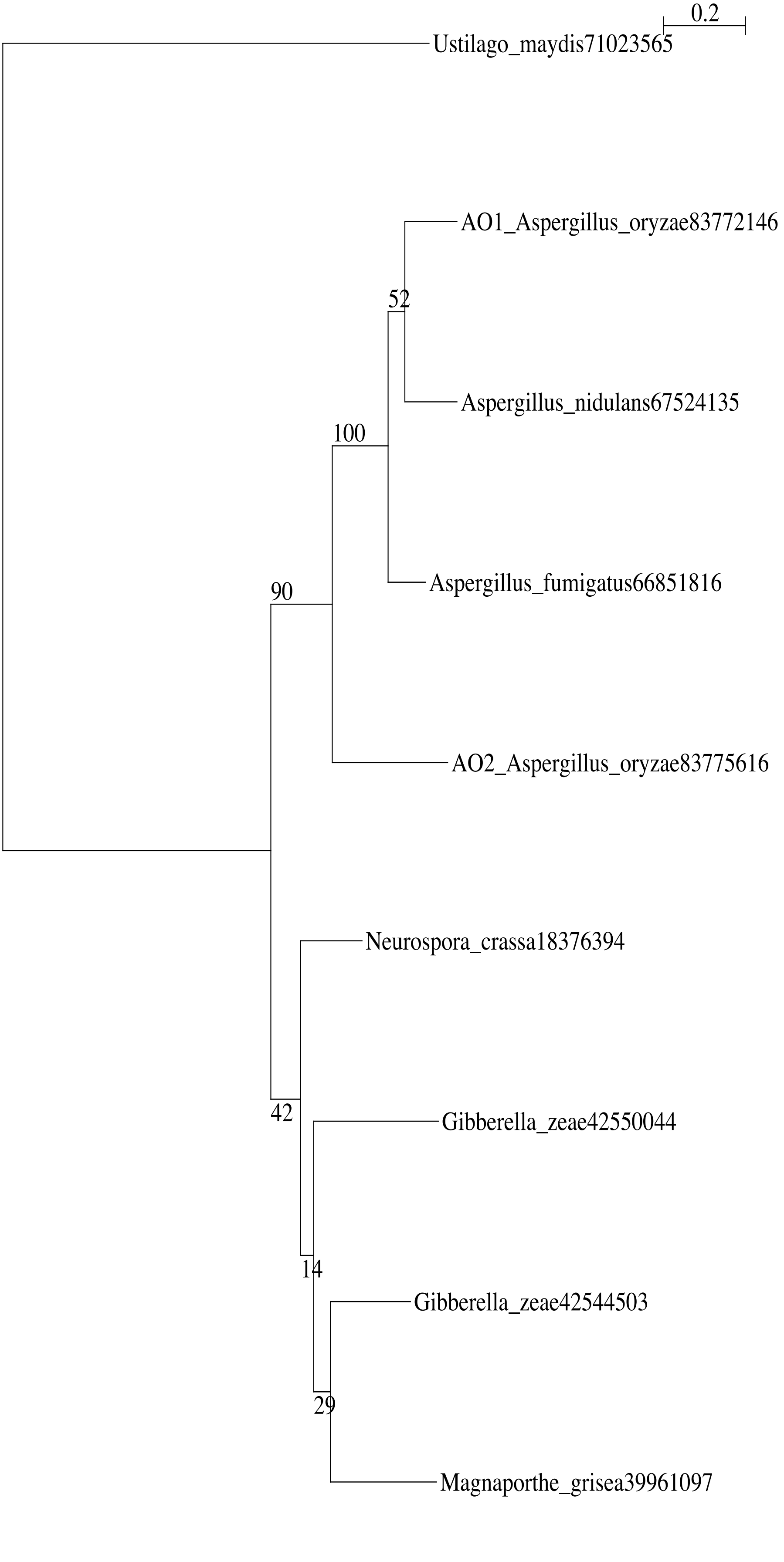
0.2
Ustilago_maydis71023565
AO1_Aspergillus_oryzae83772146
52
Aspergillus_nidulans67524135
100
Aspergillus_fumigatus66851816
90
AO2_Aspergillus_oryzae83775616
Neurospora_crassa18376394
42
Gibberella_zeae42550044
14
Gibberella_zeae42544503
29
Magnaporthe_grisea39961097

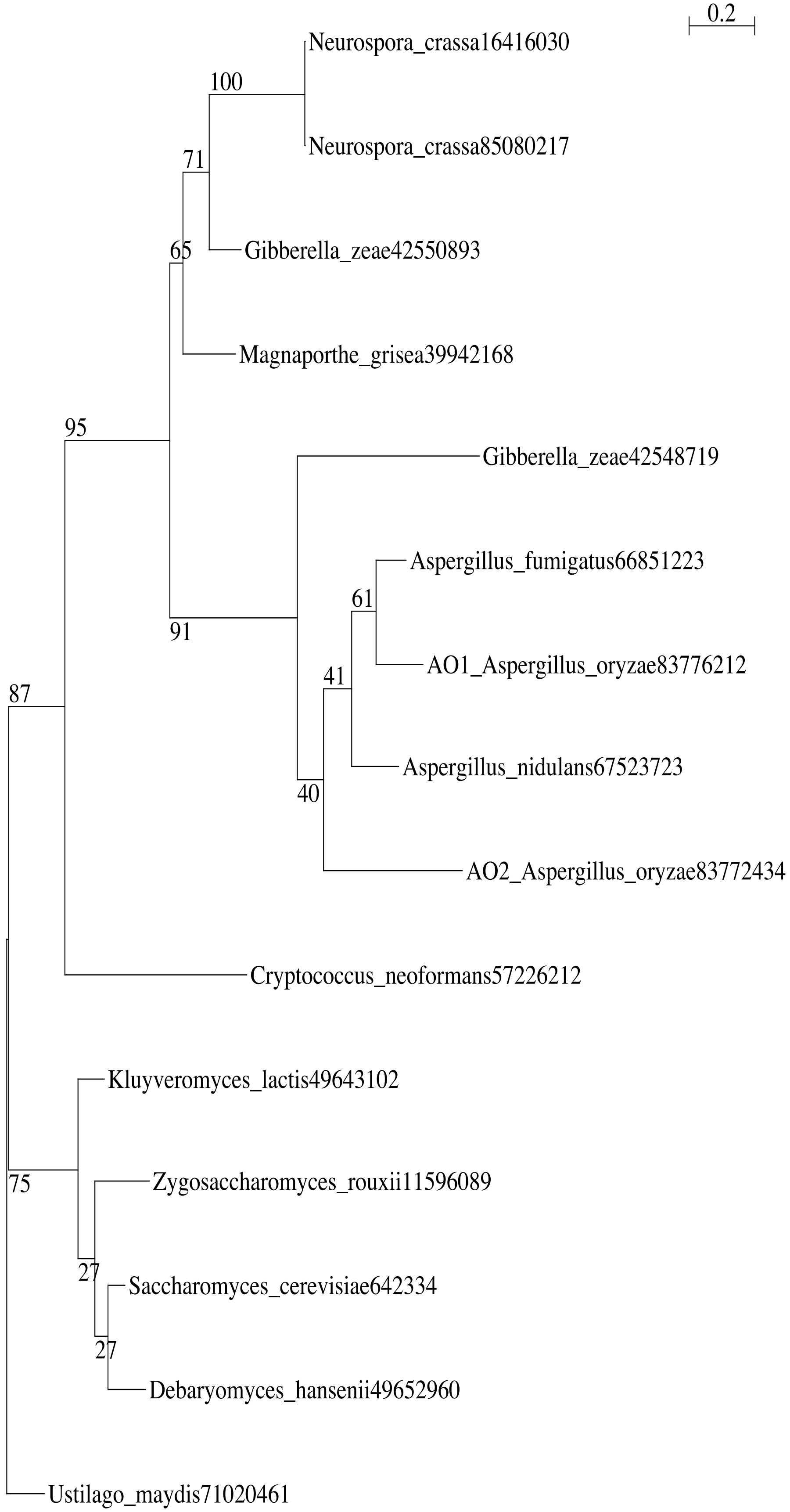
0.2
Neurospora_crassa16416030
100
Neurospora_crassa85080217
71
Gibberella_zeae42550893
65
Magnaporthe_grisea39942168
95
Gibberella_zeae42548719
Aspergillus_fumigatus66851223
61
91
AO1_Aspergillus_oryzae83776212
41
87
Aspergillus_nidulans67523723
40
AO2_Aspergillus_oryzae83772434
Cryptococcus_neoformans57226212
Kluyveromyces_lactis49643102
75
Zygosaccharomyces_rouxii11596089
27
Saccharomyces_cerevisiae642334
27
Debaryomyces_hansenii49652960
Ustilago_maydis71020461

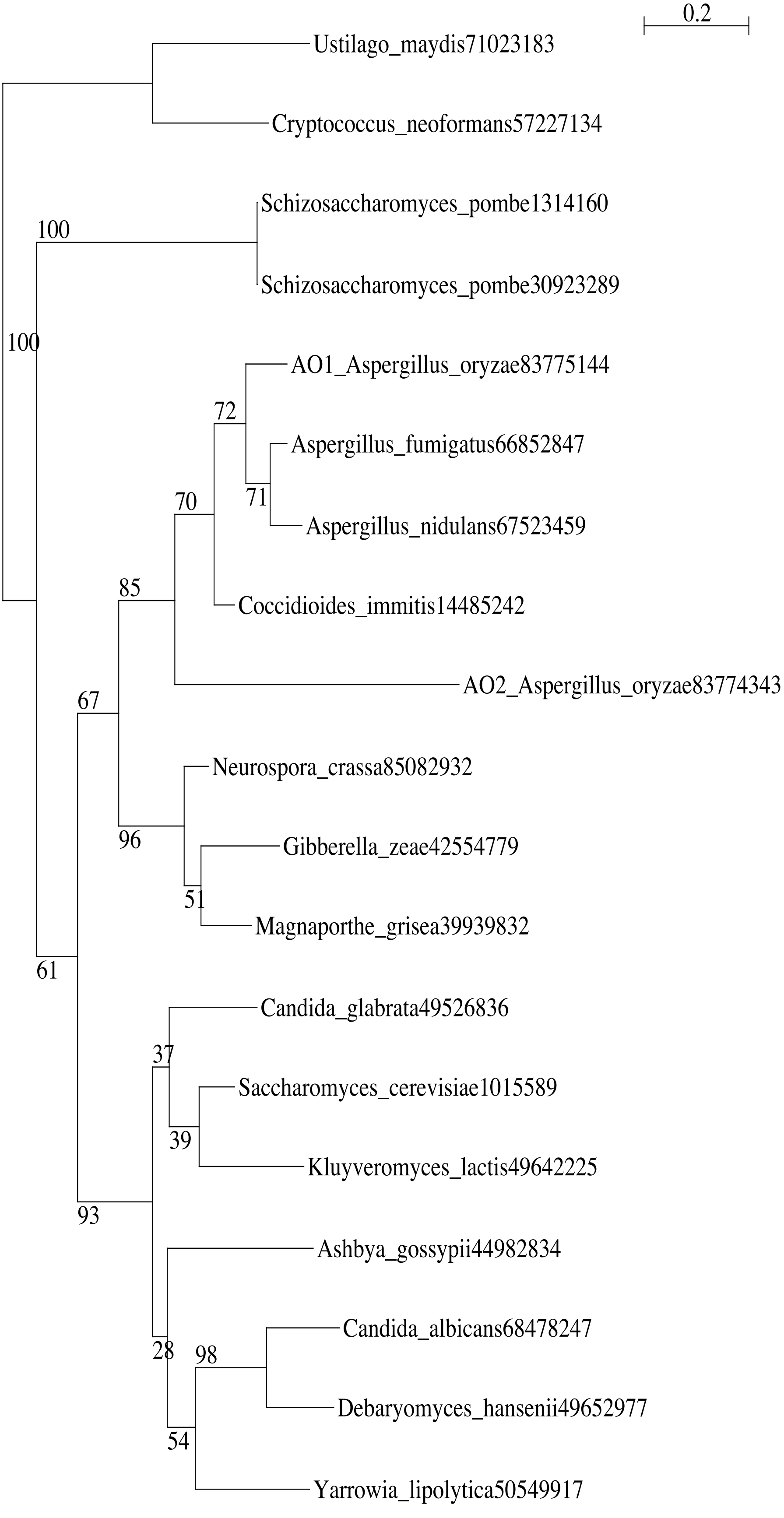

0.2
Ustilago_maydis71023183
Cryptococcus_neoformans57227134
Schizosaccharomyces_pombe1314160
Schizosaccharomyces_pombe30923289
AO1_Aspergillus_oryzae83775144
Aspergillus_fumigatus66852847
Aspergillus_nidulans67523459
Coccidioides_immitis14485242
AO2_Aspergillus_oryzae83774343
Neurospora_crassa85082932
Gibberella_zeae42554779
Magnaporthe_grisea39939832
Candida_glabrata49526836
Saccharomyces_cerevisiae1015589
Kluyveromyces_lactis49642225
Ashbya_gossypii44982834
Candida_albicans68478247
Debaryomyces_hansenii49652977
Yarrowia_lipolytica50549917
100
100
72
71
70
85
67
96
51
61
37
39
93
28
98
54

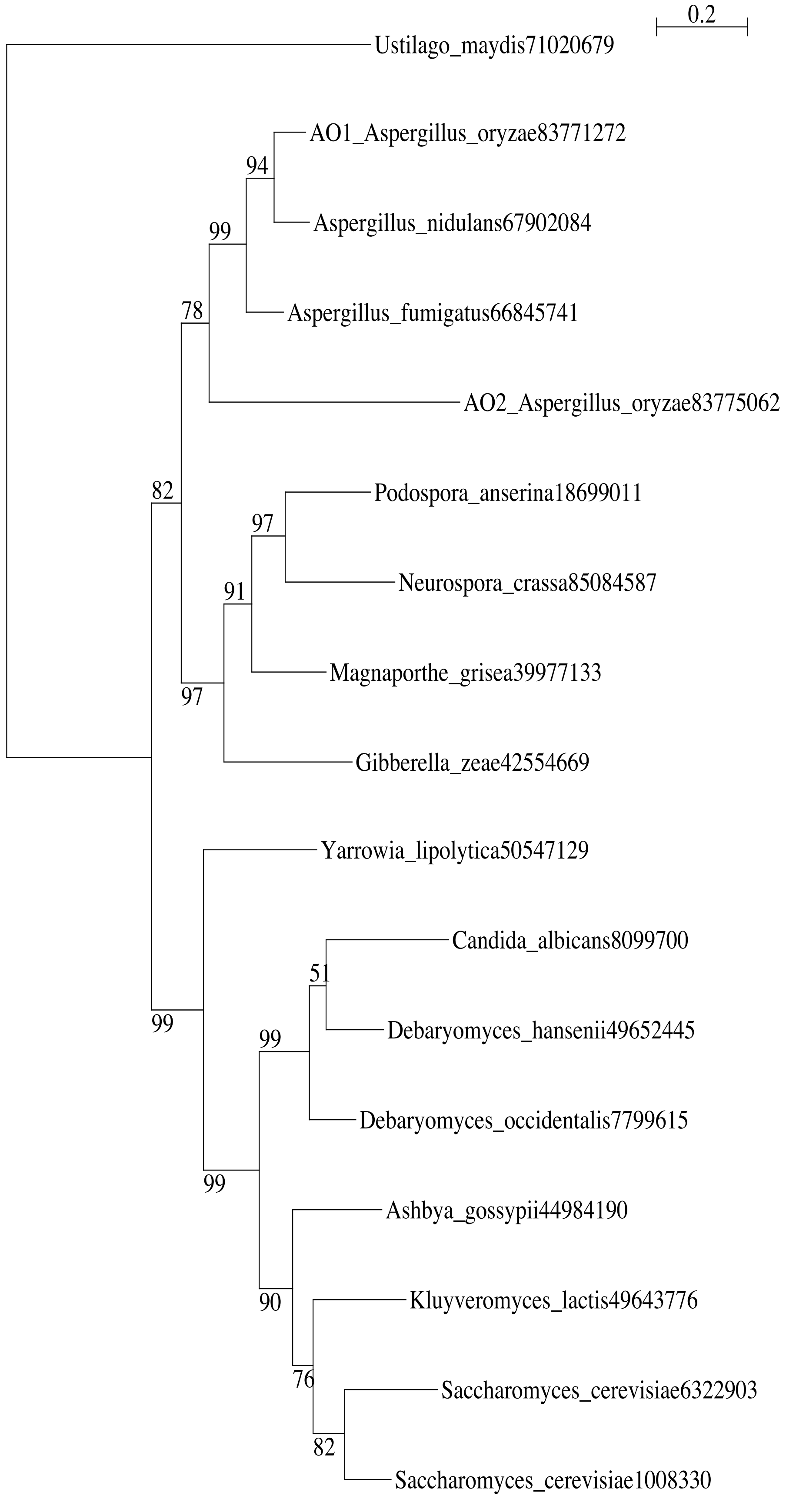
0.2
Ustilago_maydis71020679
AO1_Aspergillus_oryzae83771272
Aspergillus_nidulans67902084
Aspergillus_fumigatus66845741
AO2_Aspergillus_oryzae83775062
Podospora_anserina18699011
Neurospora_crassa85084587
Magnaporthe_grisea39977133
Gibberella_zeae42554669
Yarrowia_lipolytica50547129
Candida_albicans8099700
Debaryomyces_hansenii49652445
Debaryomyces_occidentalis7799615
Ashbya_gossypii44984190
Kluyveromyces_lactis49643776
Saccharomyces_cerevisiae6322903
Saccharomyces_cerevisiae1008330
94
99
78
82
97
91
97
51
99
99
99
90
76
82

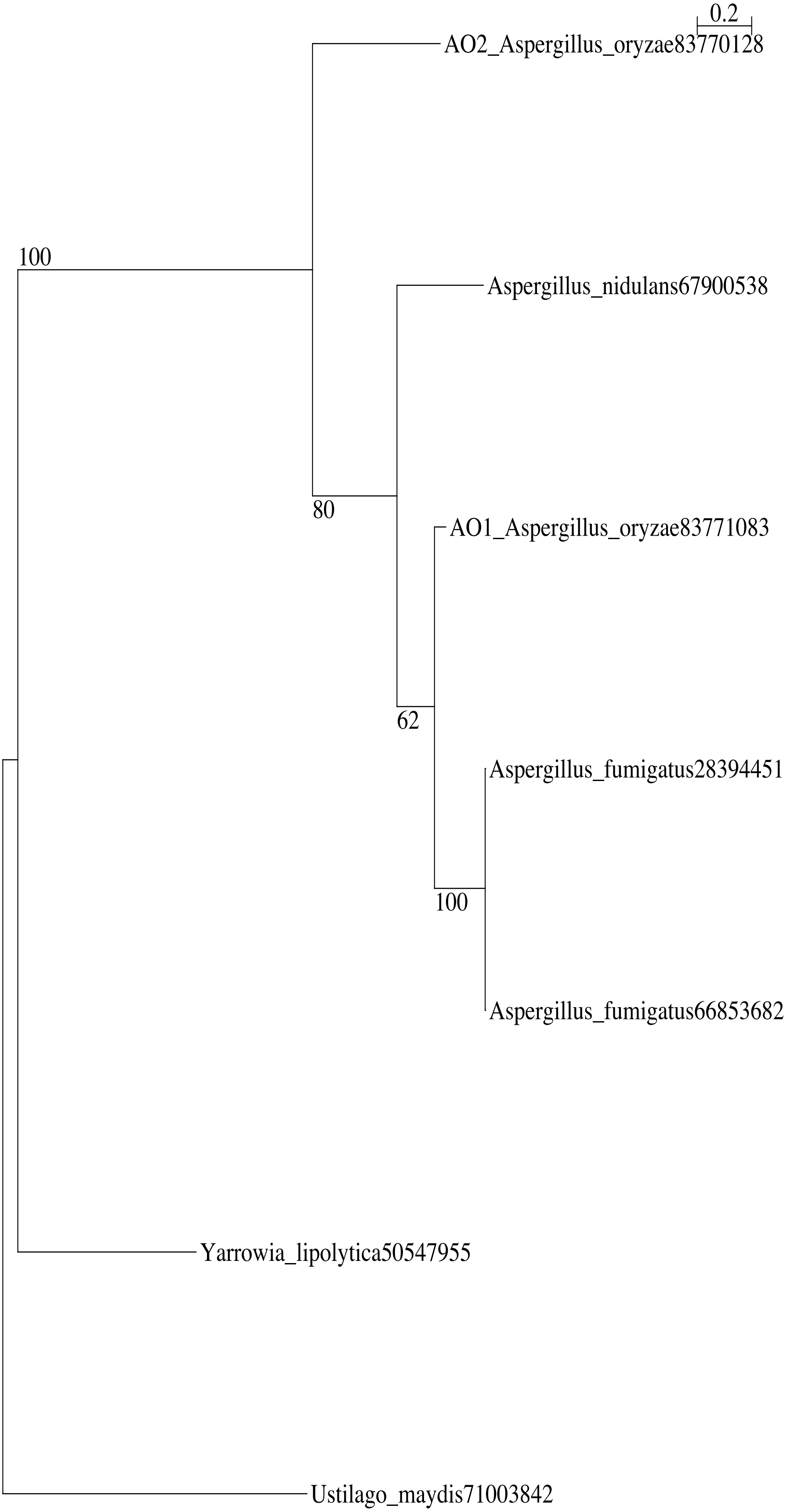
0.2
AO2_Aspergillus_oryzae83770128
100
Aspergillus_nidulans67900538
80
AO1_Aspergillus_oryzae83771083
62
Aspergillus_fumigatus28394451
100
Aspergillus_fumigatus66853682
Yarrowia_lipolytica50547955
Ustilago_maydis71003842

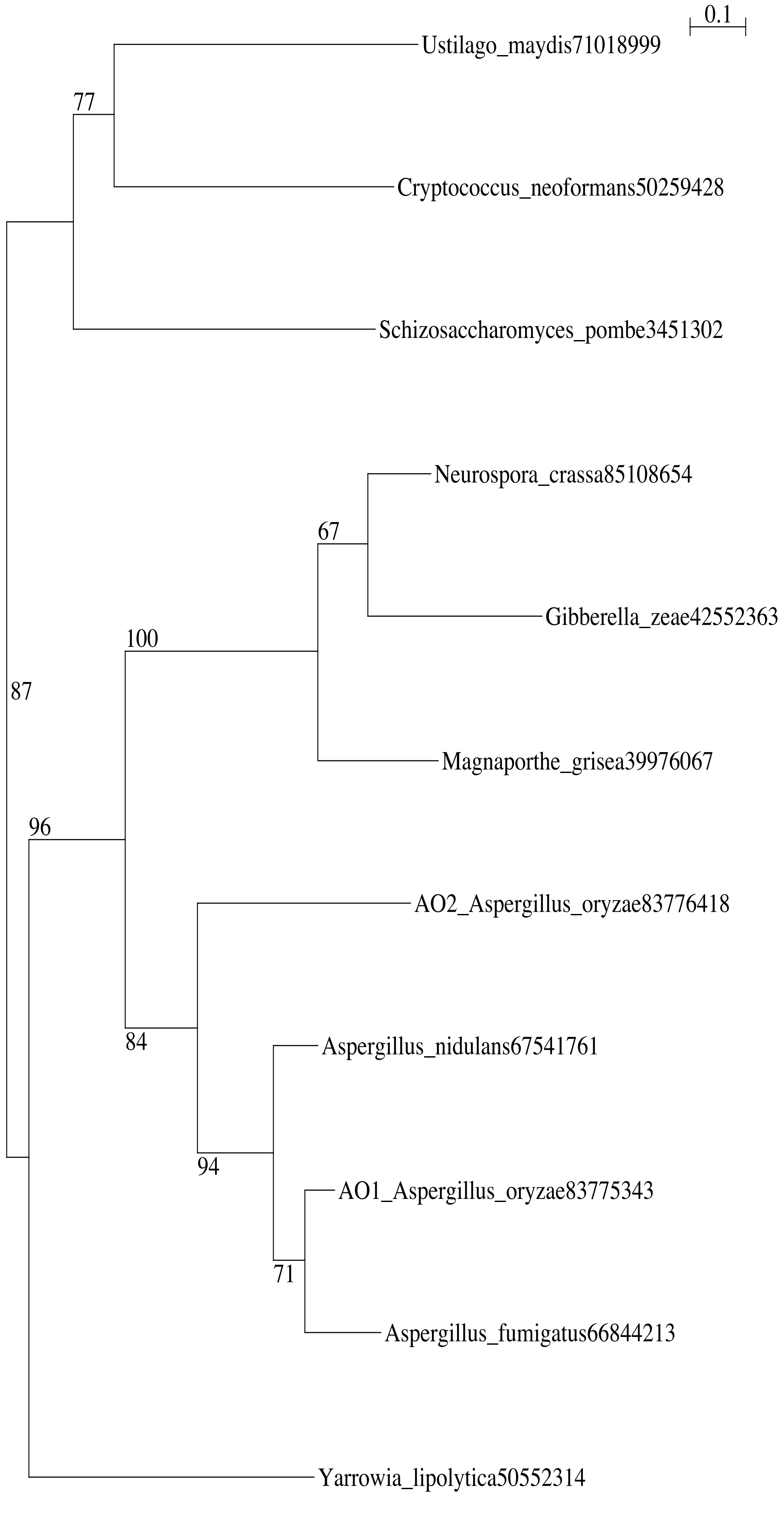
0.1
Ustilago_maydis71018999
77
Cryptococcus_neoformans50259428
Schizosaccharomyces_pombe3451302
Neurospora_crassa85108654
67
Gibberella_zeae42552363
100
87
Magnaporthe_grisea39976067
96
AO2_Aspergillus_oryzae83776418
84
Aspergillus_nidulans67541761
94
AO1_Aspergillus_oryzae83775343
71
Aspergillus_fumigatus66844213
Yarrowia_lipolytica50552314

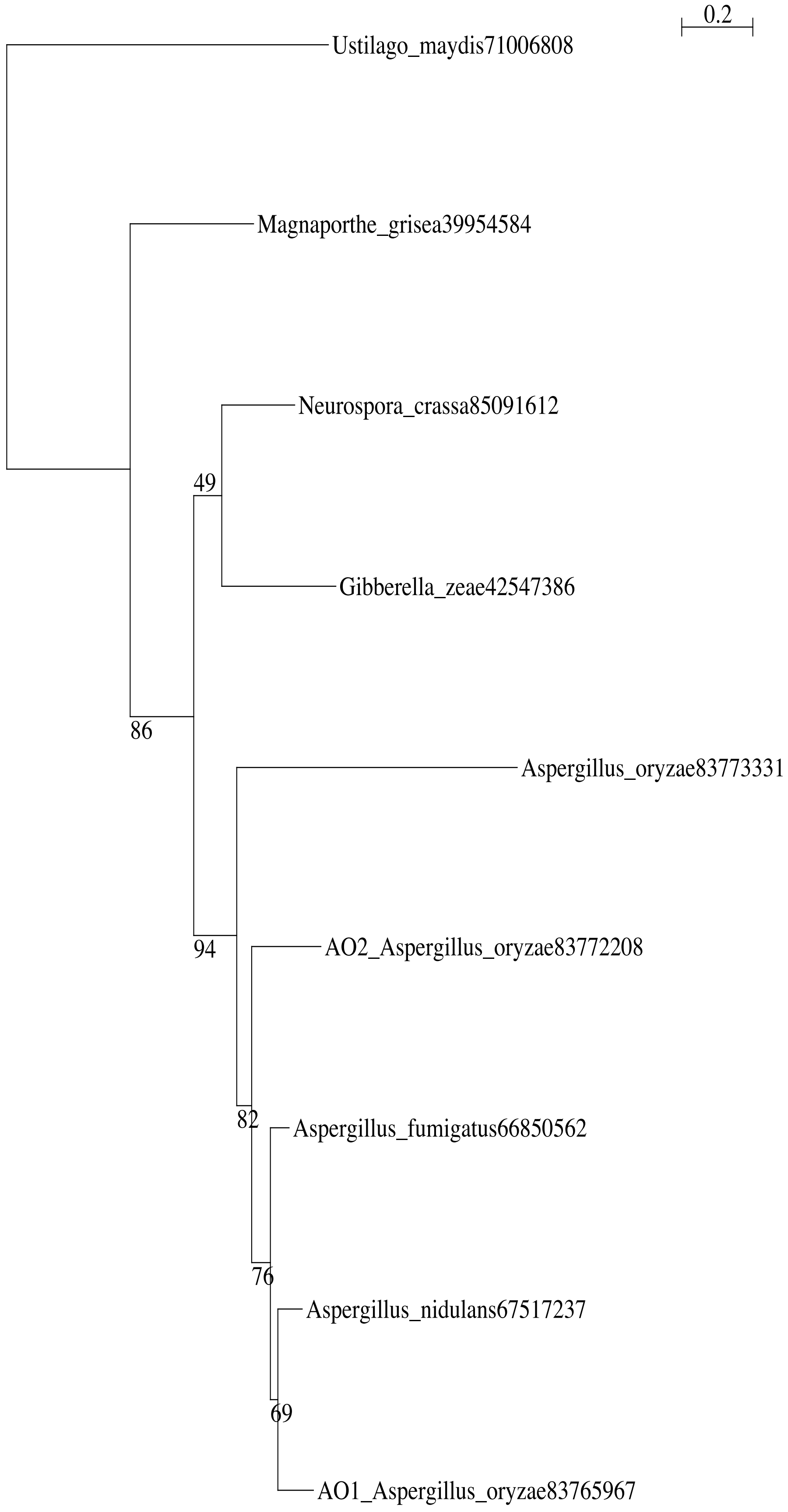

0.2
Ustilago_maydis71006808
Magnaporthe_grisea39954584
Neurospora_crassa85091612
49
Gibberella_zeae42547386
86
Aspergillus_oryzae83773331
94
AO2_Aspergillus_oryzae83772208
82
Aspergillus_fumigatus66850562
76
Aspergillus_nidulans67517237
69
AO1_Aspergillus_oryzae83765967

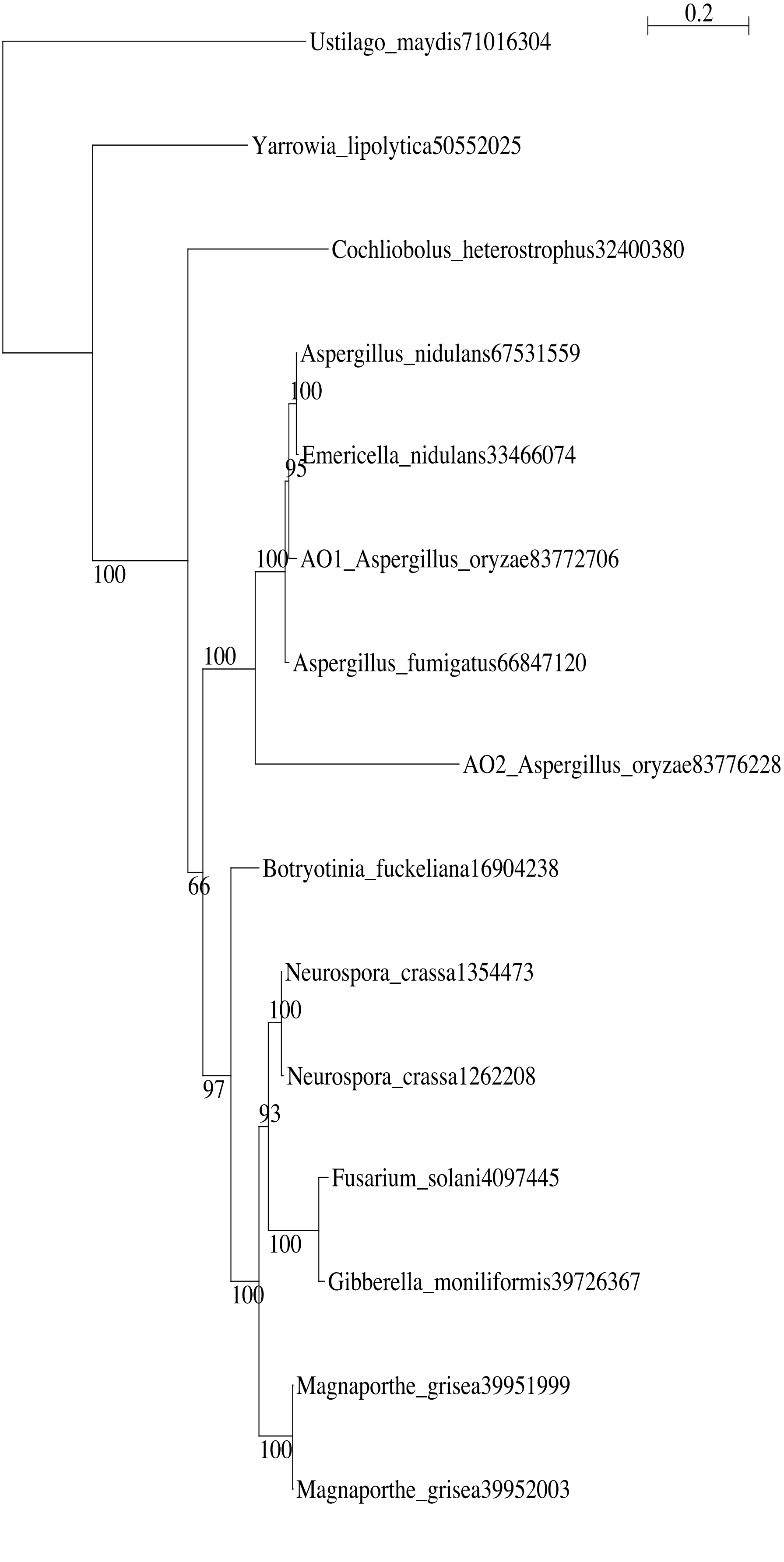
0.2
Ustilago_maydis71016304
Yarrowia_lipolytica50552025
Cochliobolus_heterostrophus32400380
Aspergillus_nidulans67531559
100
Emericella_nidulans33466074
95
100
AO1_Aspergillus_oryzae83772706
100
Aspergillus_fumigatus66847120
AO2_Aspergillus_oryzae83776228
100
Botryotinia_fuckeliana16904238
66
Neurospora_crassa1354473
100
Neurospora_crassa1262208
97
93
Fusarium_solani4097445
100
Gibberella_moniliformis39726367
100
Magnaporthe_grisea39951999
100
Magnaporthe_grisea39952003

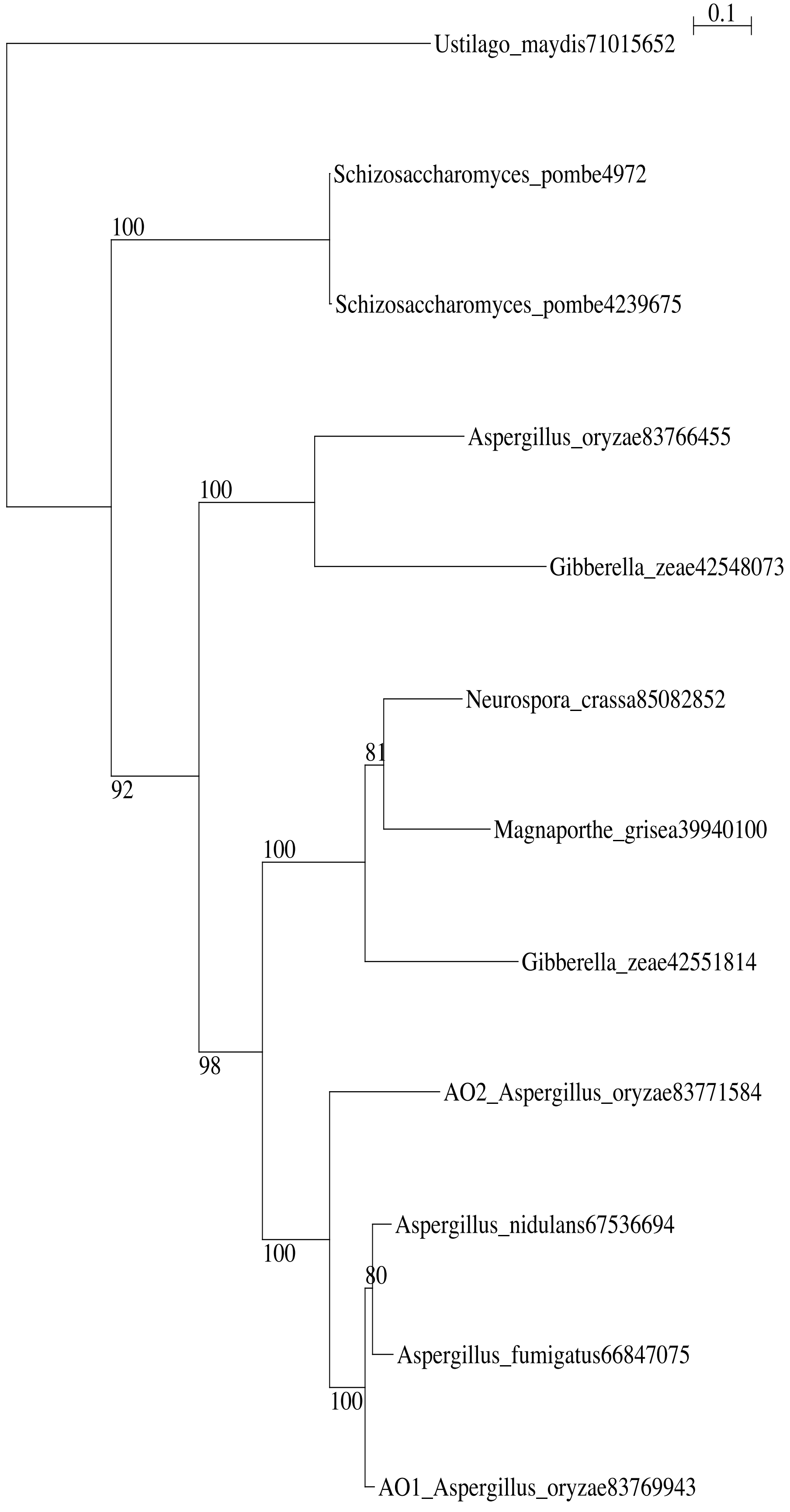
0.1
Ustilago_maydis71015652
Schizosaccharomyces_pombe4972
100
Schizosaccharomyces_pombe4239675
Aspergillus_oryzae83766455
100
Gibberella_zeae42548073
Neurospora_crassa85082852
81
92
Magnaporthe_grisea39940100
100
Gibberella_zeae42551814
98
AO2_Aspergillus_oryzae83771584
Aspergillus_nidulans67536694
100
80
Aspergillus_fumigatus66847075
100
AO1_Aspergillus_oryzae83769943

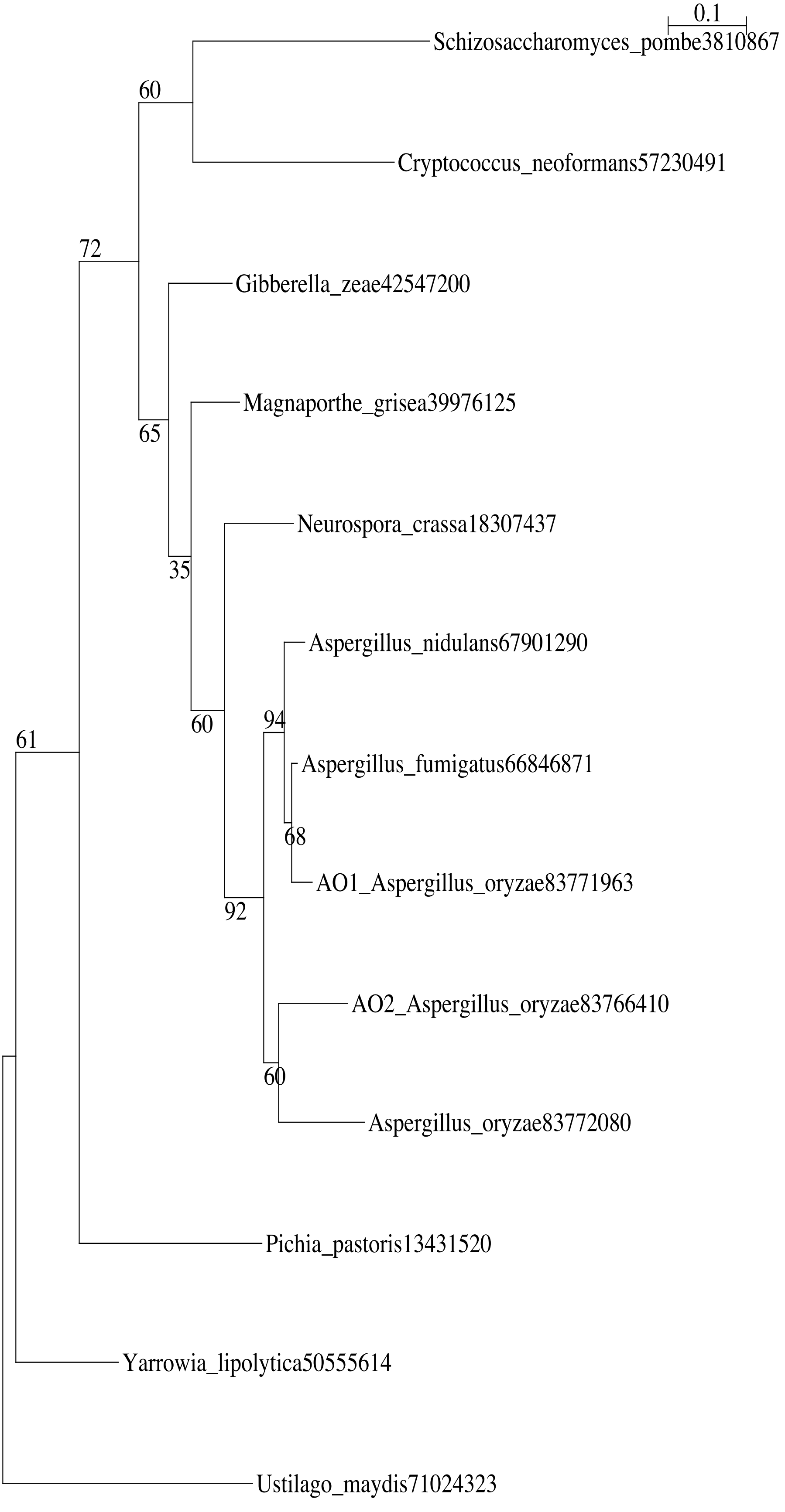

0.1
Schizosaccharomyces_pombe3810867
60
Cryptococcus_neoformans57230491
72
Gibberella_zeae42547200
Magnaporthe_grisea39976125
65
Neurospora_crassa18307437
35
Aspergillus_nidulans67901290
94
60
61
Aspergillus_fumigatus66846871
68
AO1_Aspergillus_oryzae83771963
92
AO2_Aspergillus_oryzae83766410
60
Aspergillus_oryzae83772080
Pichia_pastoris13431520
Yarrowia_lipolytica50555614
Ustilago_maydis71024323

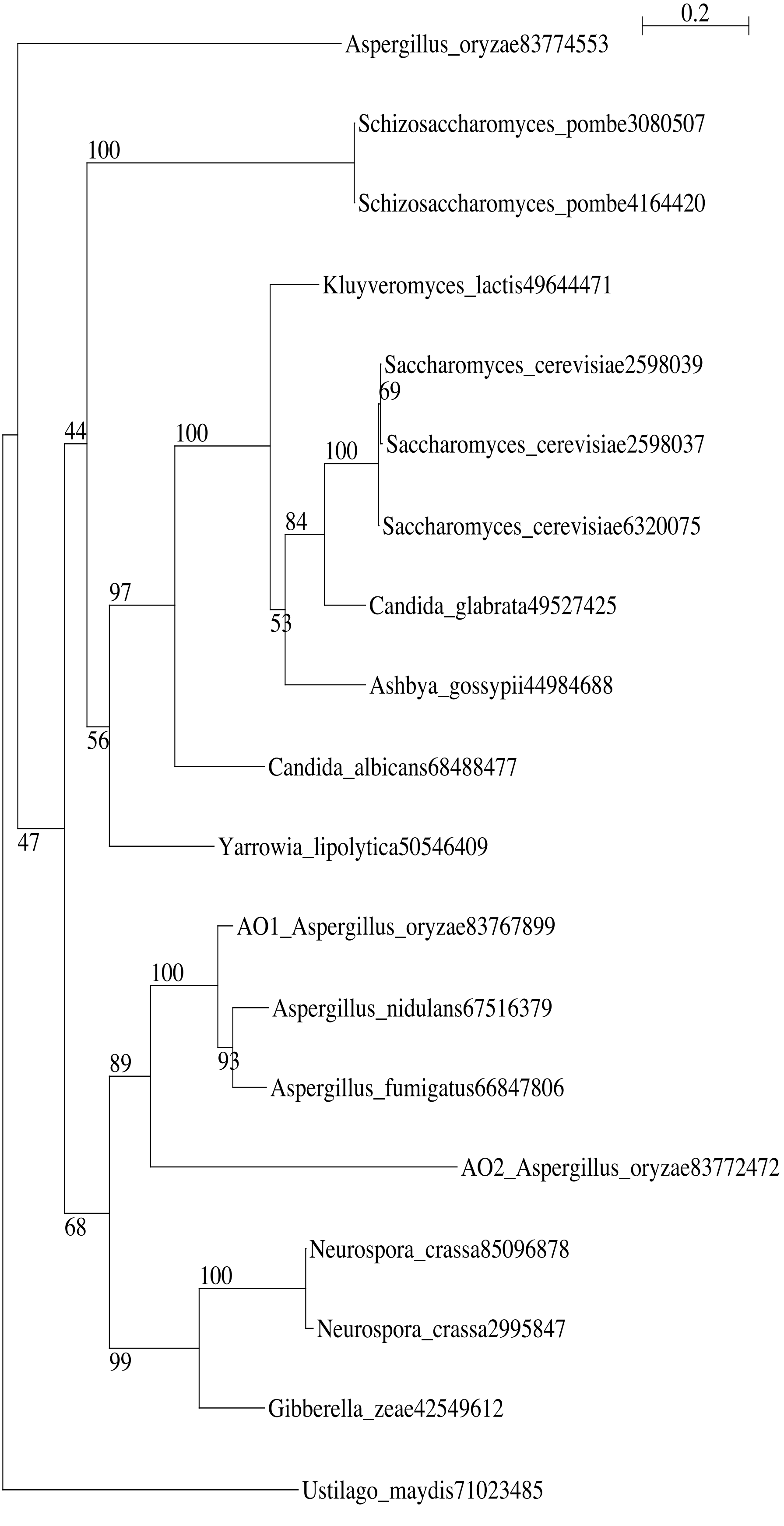
0.2
Aspergillus_oryzae83774553
100
Schizosaccharomyces_pombe3080507
Schizosaccharomyces_pombe4164420
Kluyveromyces_lactis49644471
Saccharomyces_cerevisiae2598039
69
Saccharomyces_cerevisiae2598037
44
100
100
Saccharomyces_cerevisiae6320075
84
97
Candida_glabrata49527425
53
Ashbya_gossypii44984688
56
Candida_albicans68488477
47
Yarrowia_lipolytica50546409
AO1_Aspergillus_oryzae83767899
100
Aspergillus_nidulans67516379
93
89
Aspergillus_fumigatus66847806
AO2_Aspergillus_oryzae83772472
68
Neurospora_crassa85096878
100
Neurospora_crassa2995847
99
Gibberella_zeae42549612
Ustilago_maydis71023485

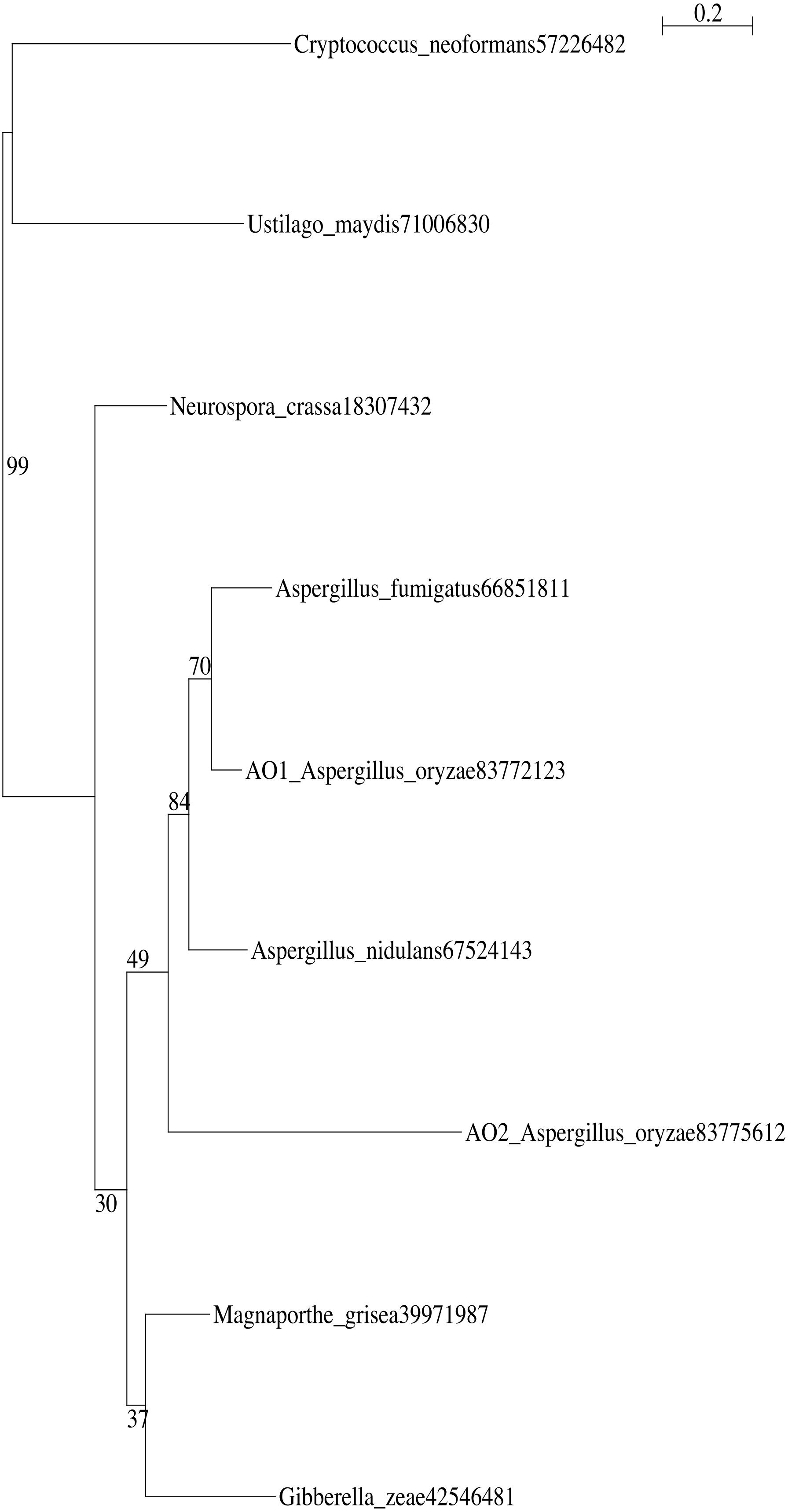
0.2
Cryptococcus_neoformans57226482
Ustilago_maydis71006830
99
Neurospora_crassa18307432
Aspergillus_fumigatus66851811
70
AO1_Aspergillus_oryzae83772123
84
Aspergillus_nidulans67524143
49
AO2_Aspergillus_oryzae83775612
30
Magnaporthe_grisea39971987
37
Gibberella_zeae42546481

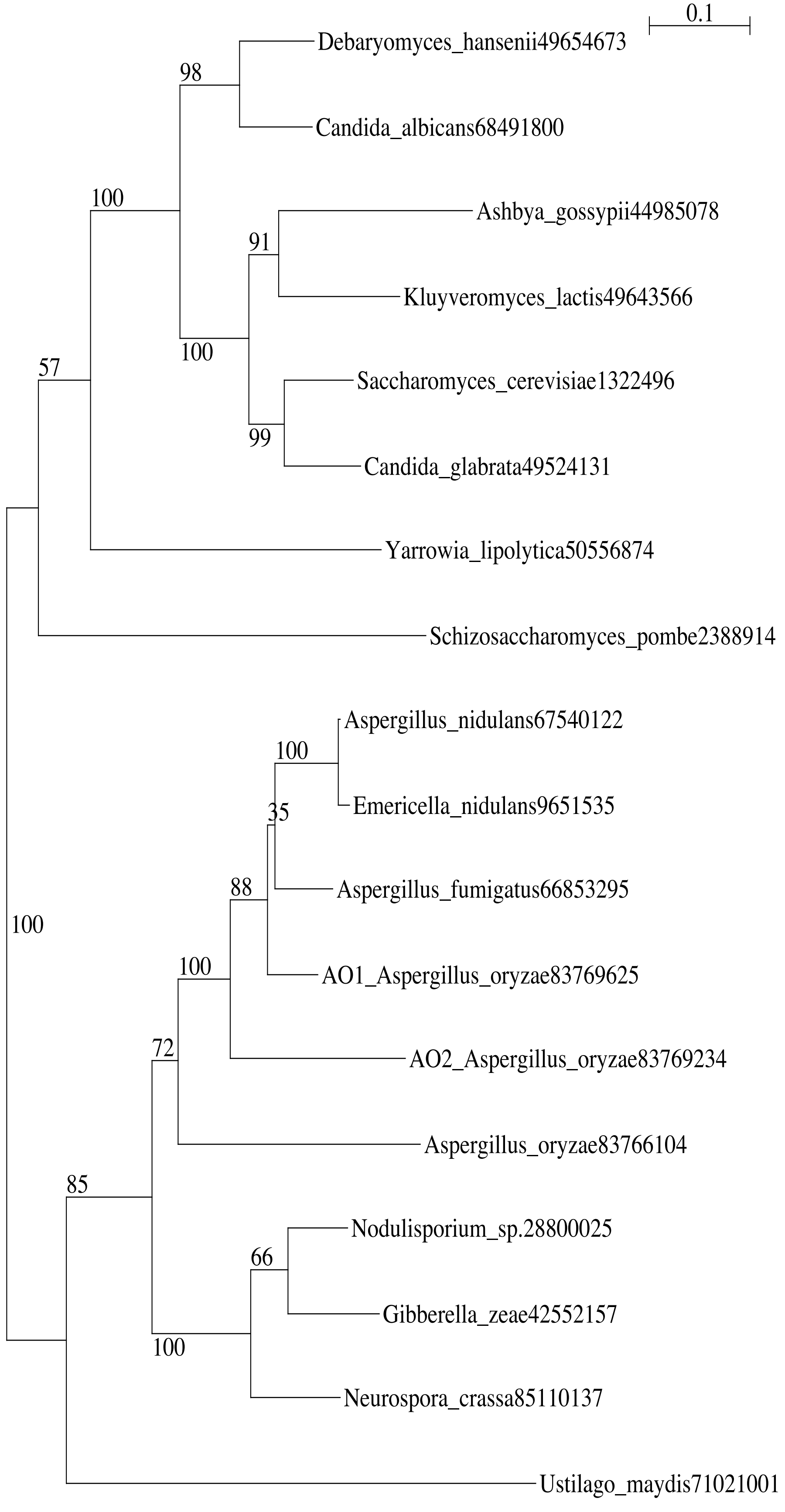
0.1
Debaryomyces_hansenii49654673
Candida_albicans68491800
Ashbya_gossypii44985078
Kluyveromyces_lactis49643566
Saccharomyces_cerevisiae1322496
Candida_glabrata49524131
Yarrowia_lipolytica50556874
Schizosaccharomyces_pombe2388914
Aspergillus_nidulans67540122
Emericella_nidulans9651535
Aspergillus_fumigatus66853295
AO1_Aspergillus_oryzae83769625
AO2_Aspergillus_oryzae83769234
Aspergillus_oryzae83766104
Nodulisporium_sp.28800025
Gibberella_zeae42552157
Neurospora_crassa85110137
Ustilago_maydis71021001
98
100
91
100
99
57
100
35
88
100
72
85
66
100
100

Trees classified as Topology B in *Aspergillus nidulans* (9).

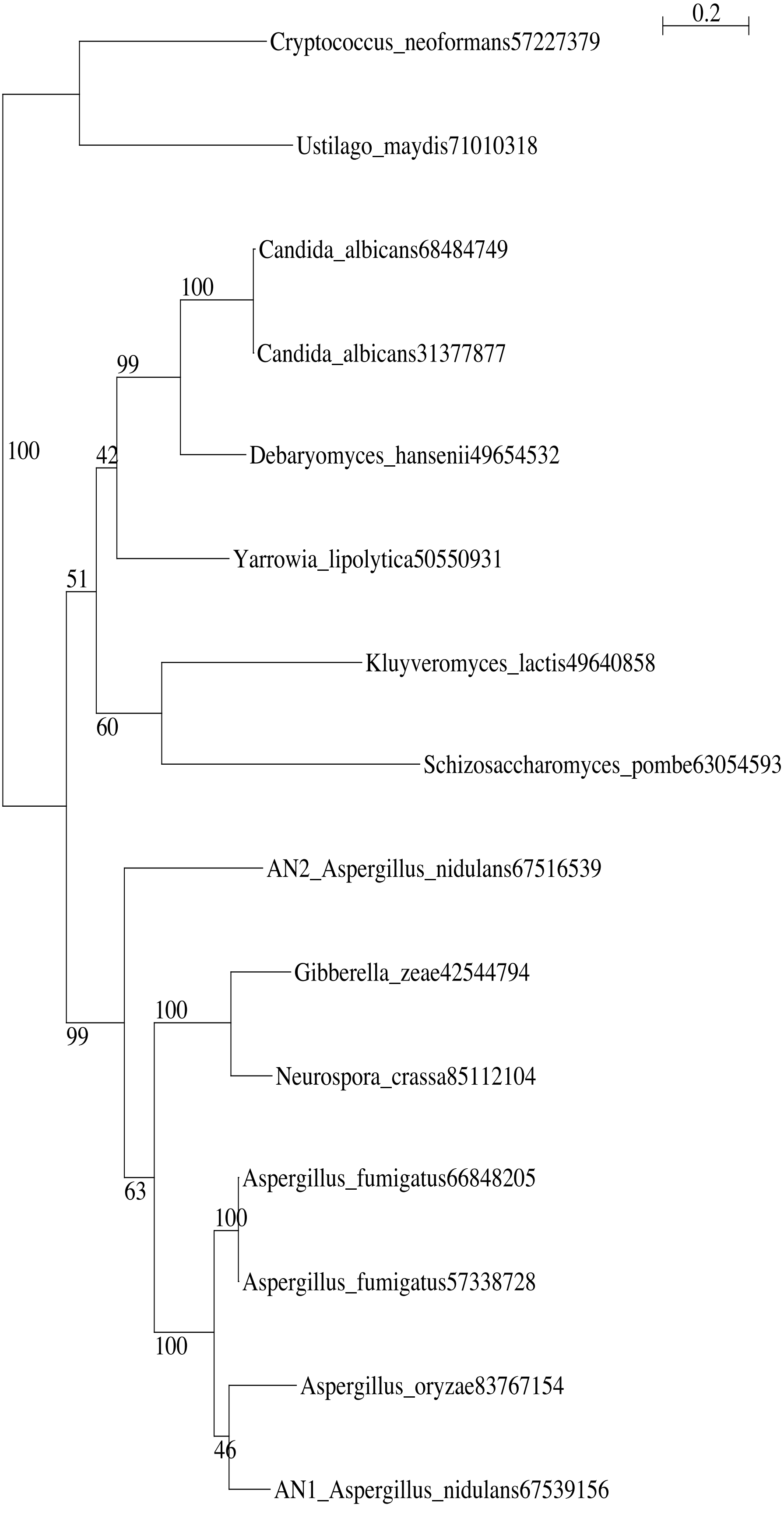
0.2
Cryptococcus_neoformans57227379
Ustilago_maydis71010318
Candida_albicans68484749
100
Candida_albicans31377877
99
Debaryomyces_hansenii49654532
100
42
Yarrowia_lipolytica50550931
51
Kluyveromyces_lactis49640858
60
Schizosaccharomyces_pombe63054593
AN2_Aspergillus_nidulans67516539
Gibberella_zeae42544794
100
99
Neurospora_crassa85112104
Aspergillus_fumigatus66848205
63
100
Aspergillus_fumigatus57338728
100
Aspergillus_oryzae83767154
46
AN1_Aspergillus_nidulans67539156

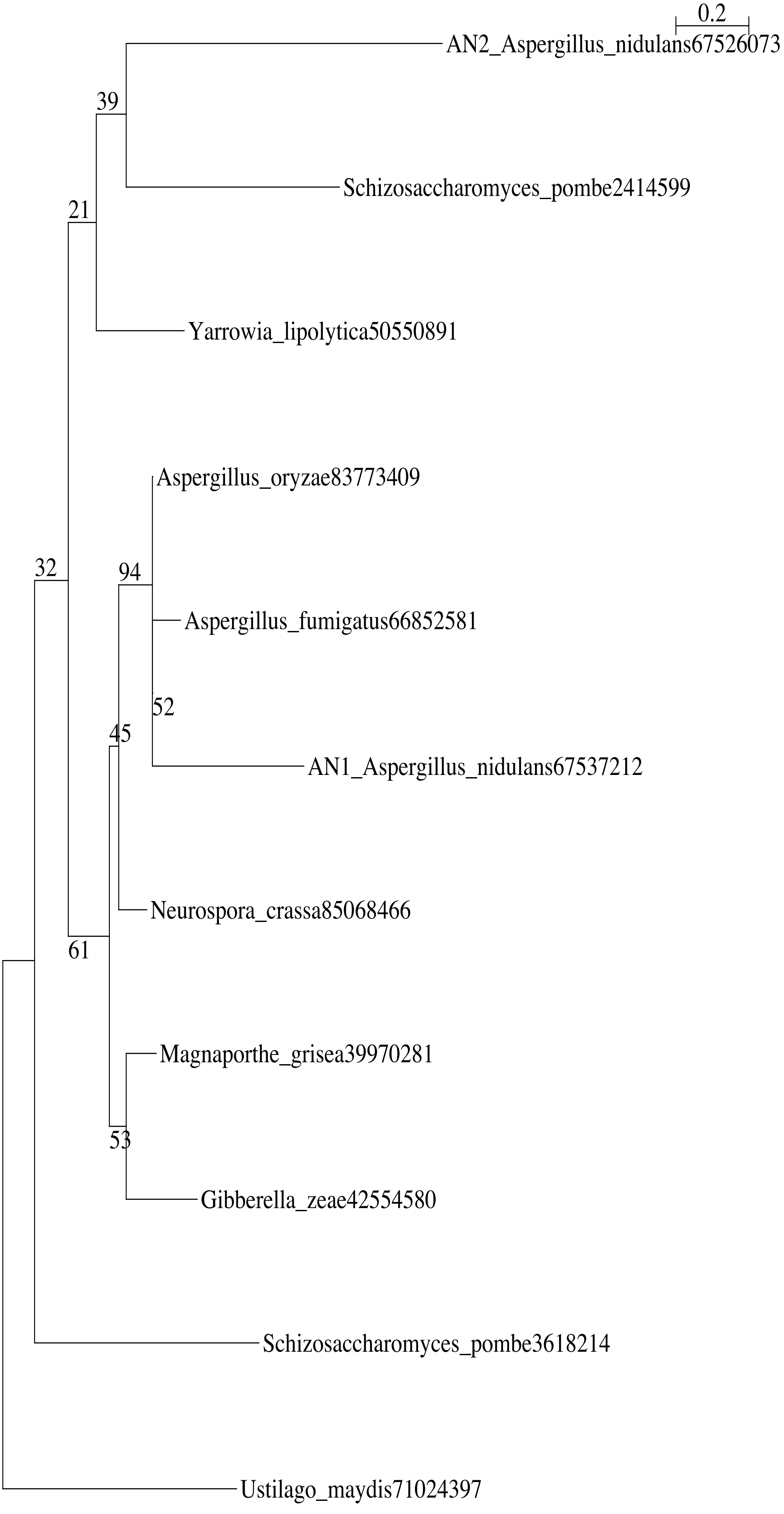
0.2
AN2_Aspergillus_nidulans67526073
39
Schizosaccharomyces_pombe2414599
21
Yarrowia_lipolytica50550891
Aspergillus_oryzae83773409
32
94
Aspergillus_fumigatus66852581
52
45
AN1_Aspergillus_nidulans67537212
Neurospora_crassa85068466
61
Magnaporthe_grisea39970281
53
Gibberella_zeae42554580
Schizosaccharomyces_pombe3618214
Ustilago_maydis71024397

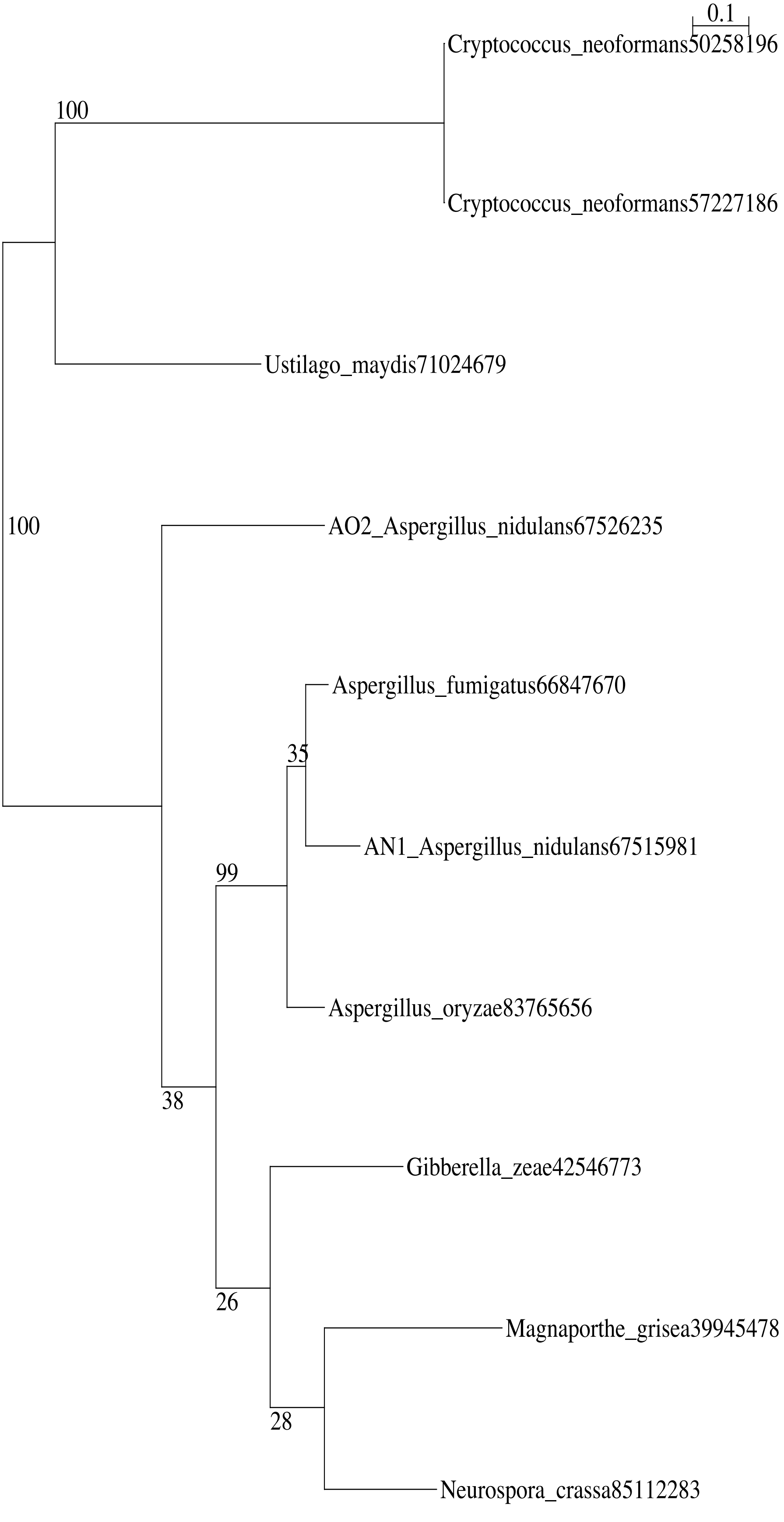
0.1
Cryptococcus_neoformans50258196
Cryptococcus_neoformans57227186
100
Ustilago_maydis71024679
100
AO2_Aspergillus_nidulans67526235
Aspergillus_fumigatus66847670
35
AN1_Aspergillus_nidulans67515981
99
Aspergillus_oryzae83765656
38
Gibberella_zeae42546773
26
Magnaporthe_grisea39945478
28
Neurospora_crassa85112283

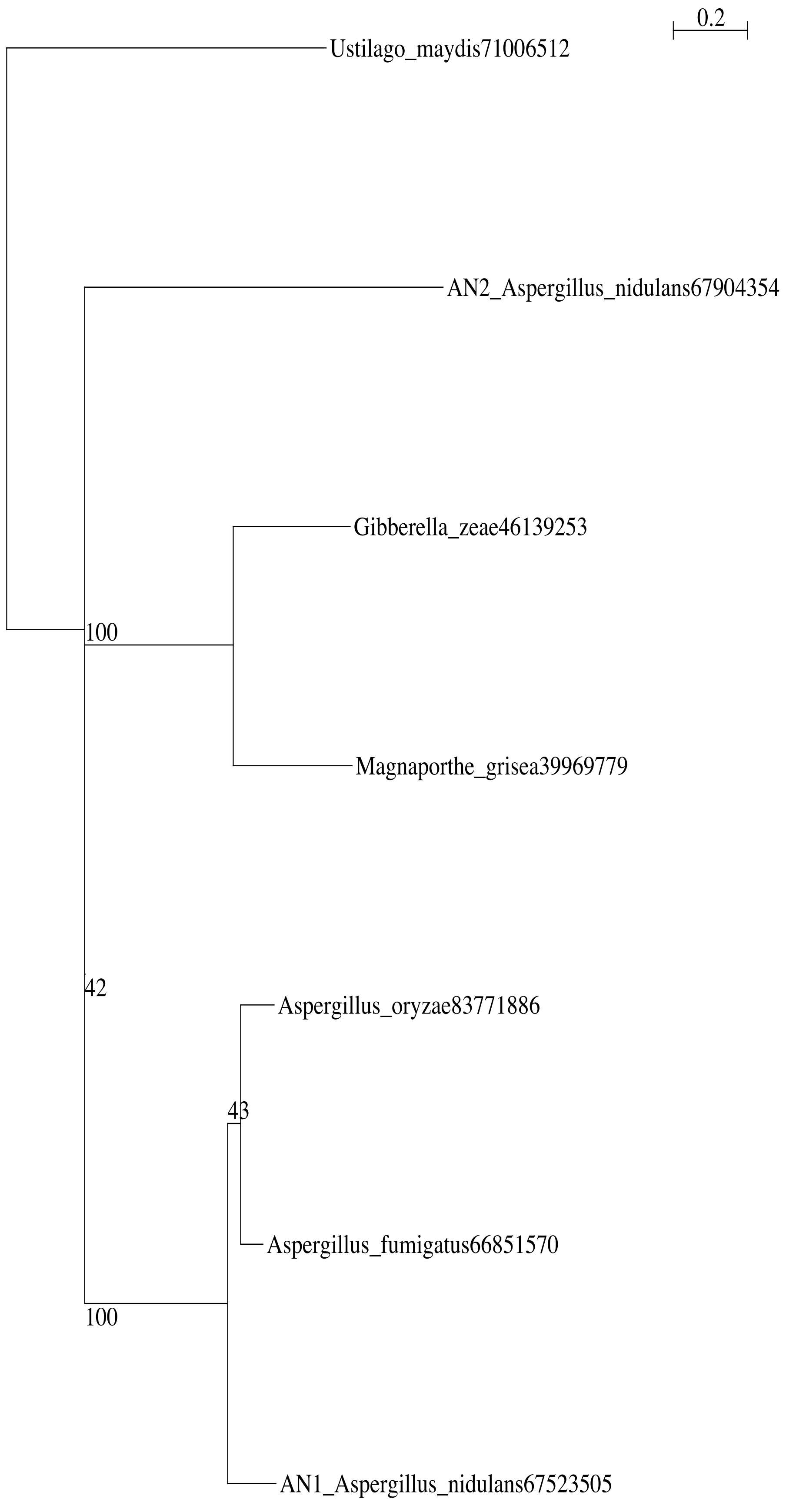
0.2
Ustilago_maydis71006512
AN2_Aspergillus_nidulans67904354
Gibberella_zeae46139253
100
Magnaporthe_grisea39969779
42
Aspergillus_oryzae83771886
43
Aspergillus_fumigatus66851570
100
AN1_Aspergillus_nidulans67523505

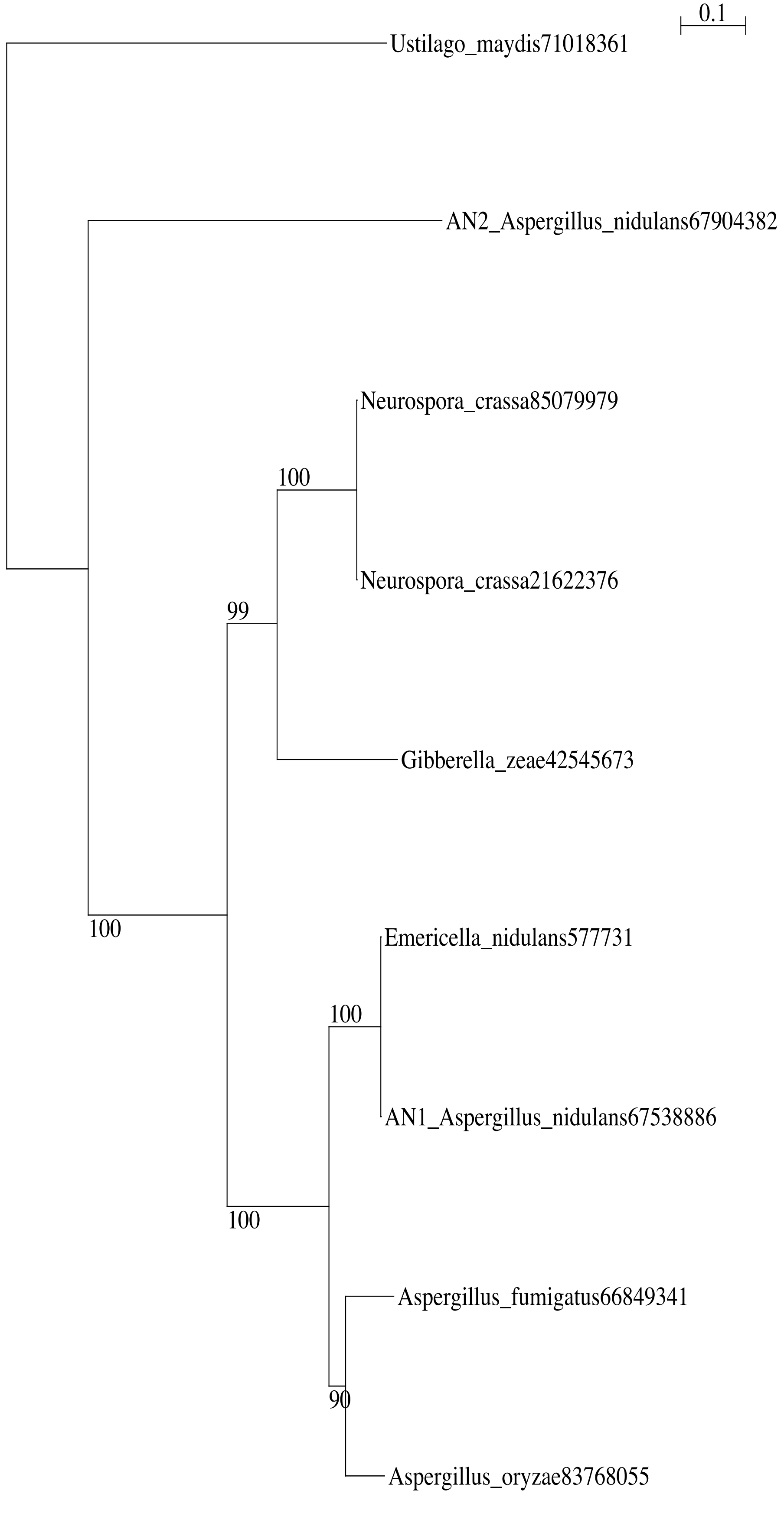
0.1
Ustilago_maydis71018361
AN2_Aspergillus_nidulans67904382
Neurospora_crassa85079979
100
Neurospora_crassa21622376
99
Gibberella_zeae42545673
100
Emericella_nidulans577731
100
AN1_Aspergillus_nidulans67538886
100
Aspergillus_fumigatus66849341
90
Aspergillus_oryzae83768055

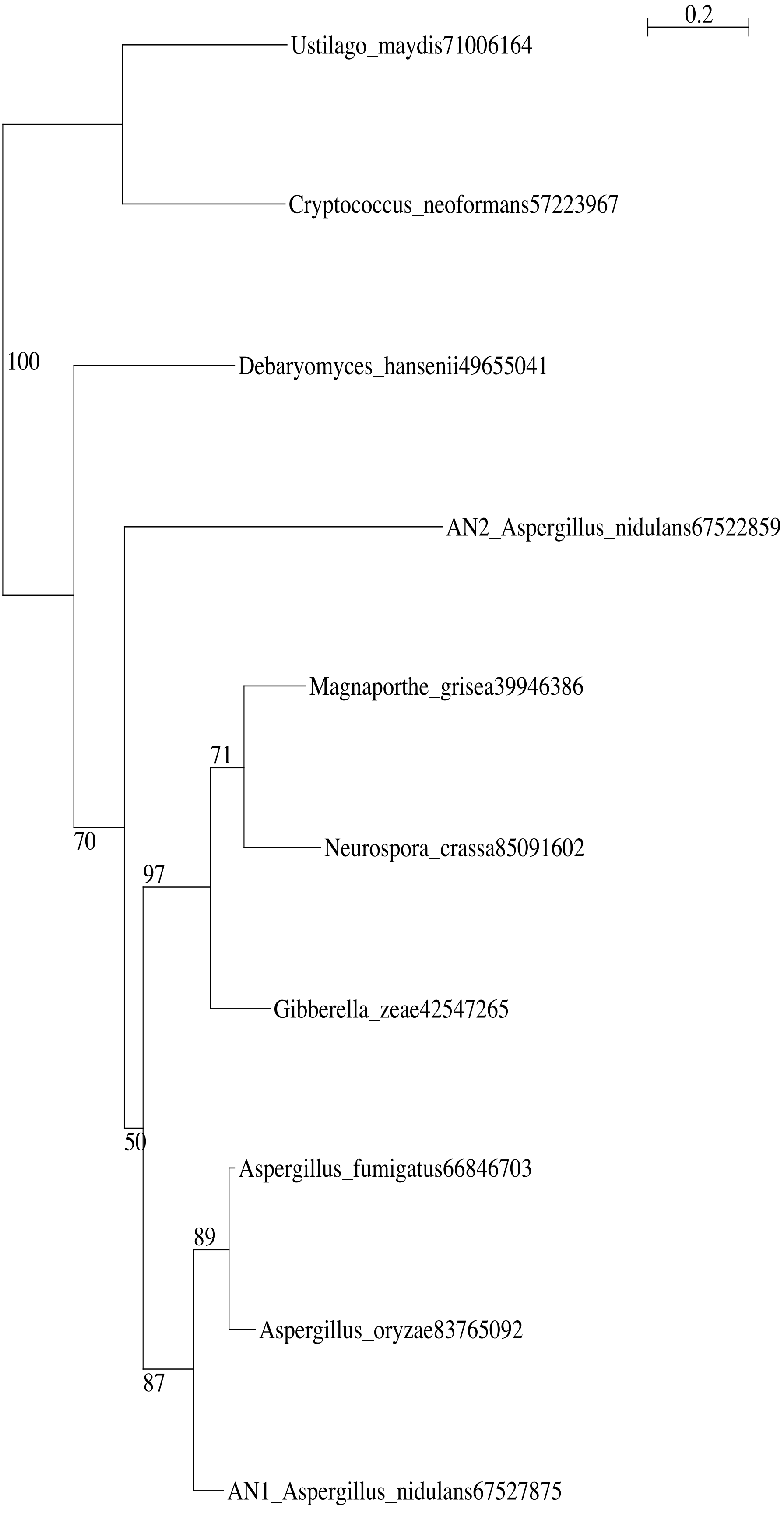
0.2
Ustilago_maydis71006164
Cryptococcus_neoformans57223967
100
Debaryomyces_hansenii49655041
AN2_Aspergillus_nidulans67522859
Magnaporthe_grisea39946386
71
70
Neurospora_crassa85091602
97
Gibberella_zeae42547265
50
Aspergillus_fumigatus66846703
89
Aspergillus_oryzae83765092
87
AN1_Aspergillus_nidulans67527875

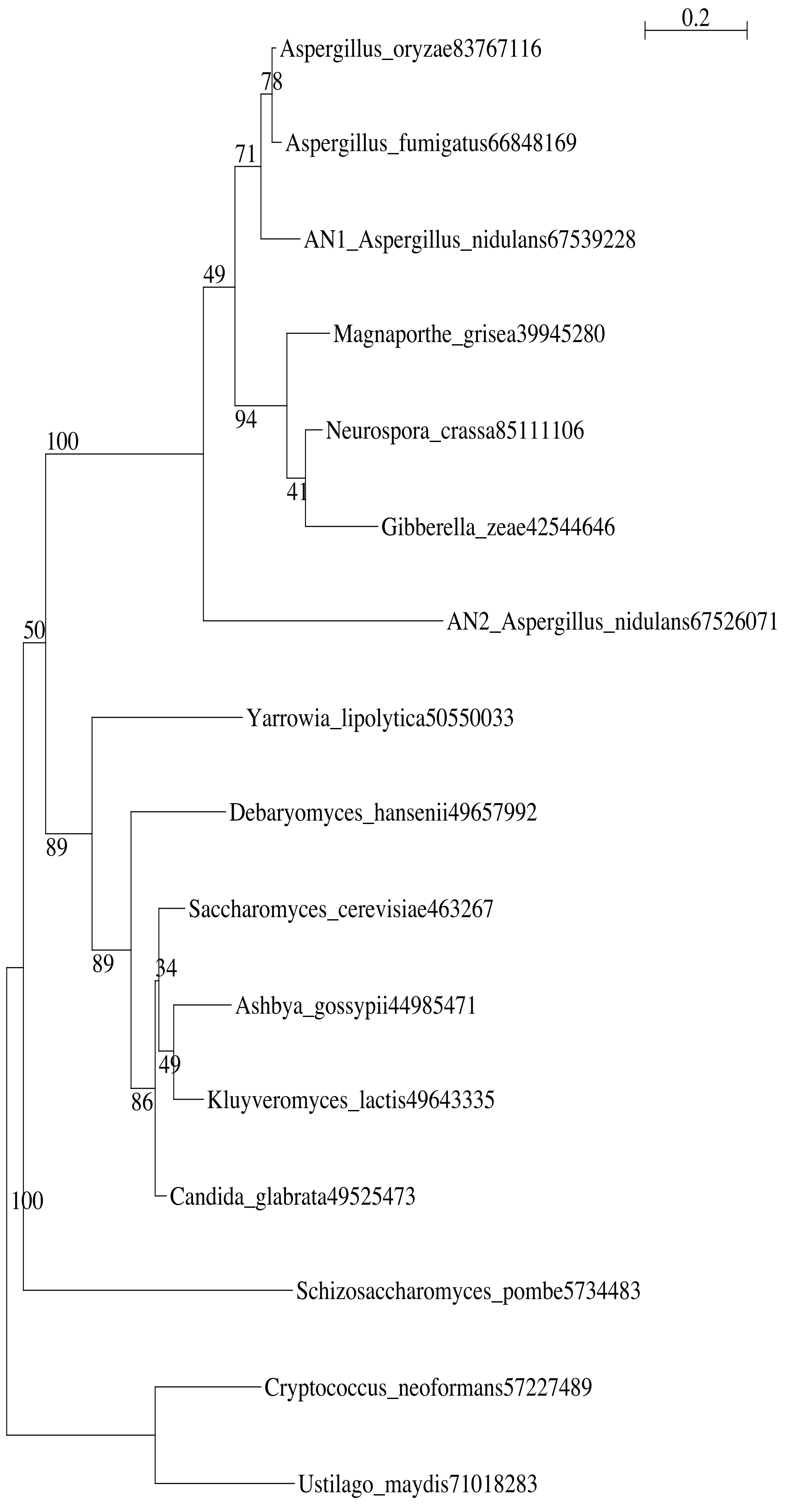
0.2
Aspergillus_oryzae83767116
78
Aspergillus_fumigatus66848169
71
AN1_Aspergillus_nidulans67539228
49
Magnaporthe_grisea39945280
94
Neurospora_crassa85111106
41
Gibberella_zeae42544646
100
AN2_Aspergillus_nidulans67526071
50
Yarrowia_lipolytica50550033
89
Debaryomyces_hansenii49657992
Saccharomyces_cerevisiae463267
89
34
Ashbya_gossypii44985471
49
Kluyveromyces_lactis49643335
86
Candida_glabrata49525473
100
Schizosaccharomyces_pombe5734483
Cryptococcus_neoformans57227489
Ustilago_maydis71018283

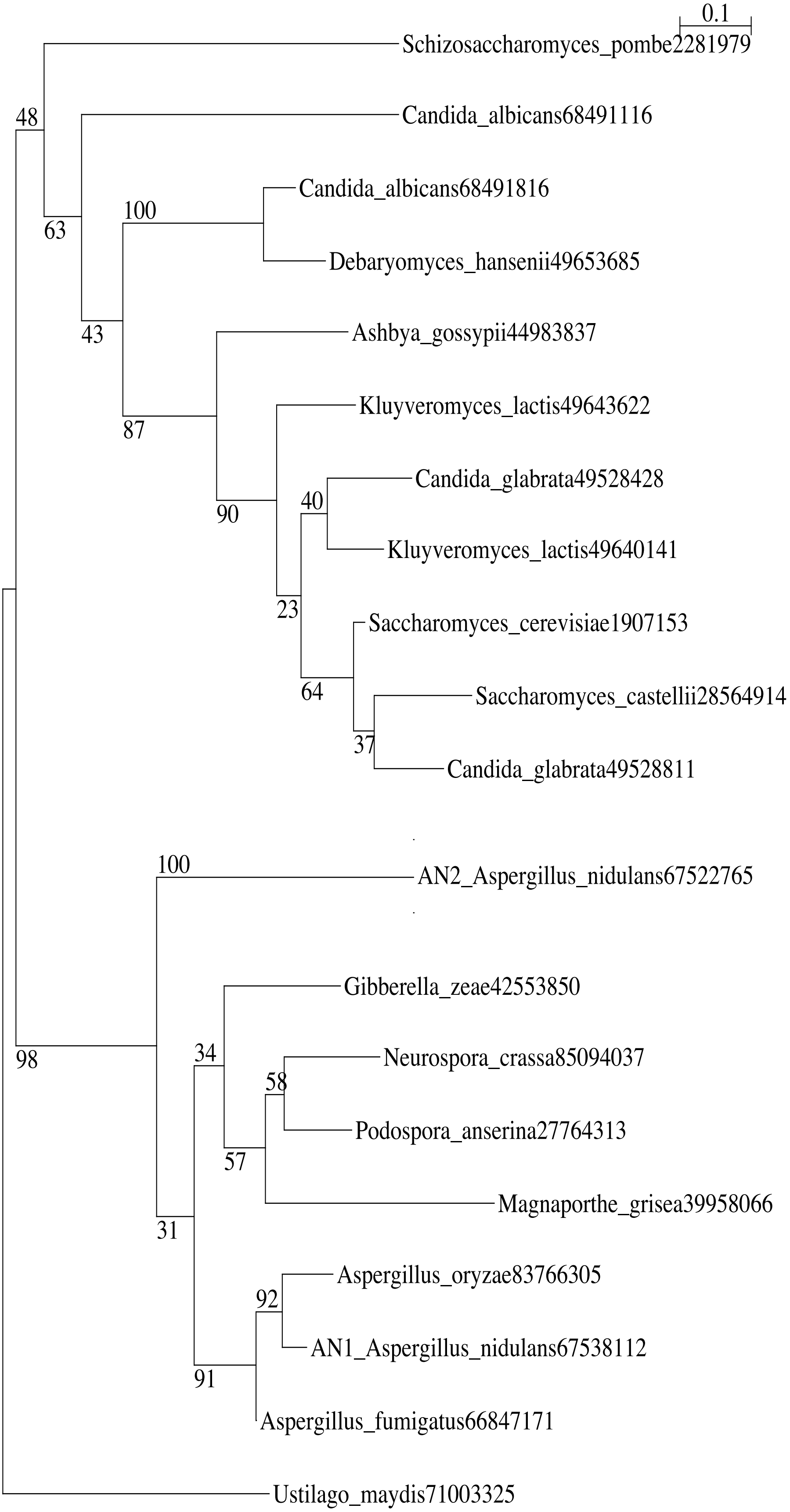
0.1
Schizosaccharomyces_pombe2281979
Candida_albicans68491116
Candida_albicans68491816
Debaryomyces_hansenii49653685
Ashbya_gossypii44983837
Kluyveromyces_lactis49643622
Candida_glabrata49528428
Kluyveromyces_lactis49640141
Saccharomyces_cerevisiae1907153
Saccharomyces_castellii28564914
Candida_glabrata49528811
AN2_Aspergillus_nidulans67522765
Gibberella_zeae42553850
Neurospora_crassa85094037
Podospora_anserina27764313
Magnaporthe_grisea39958066
Aspergillus_oryzae83766305
AN1_Aspergillus_nidulans67538112
Aspergillus_fumigatus66847171
Ustilago_maydis71003325
48
63
100
43
87
90
40
23
64
37
100
98
34
58
57
31
92
91

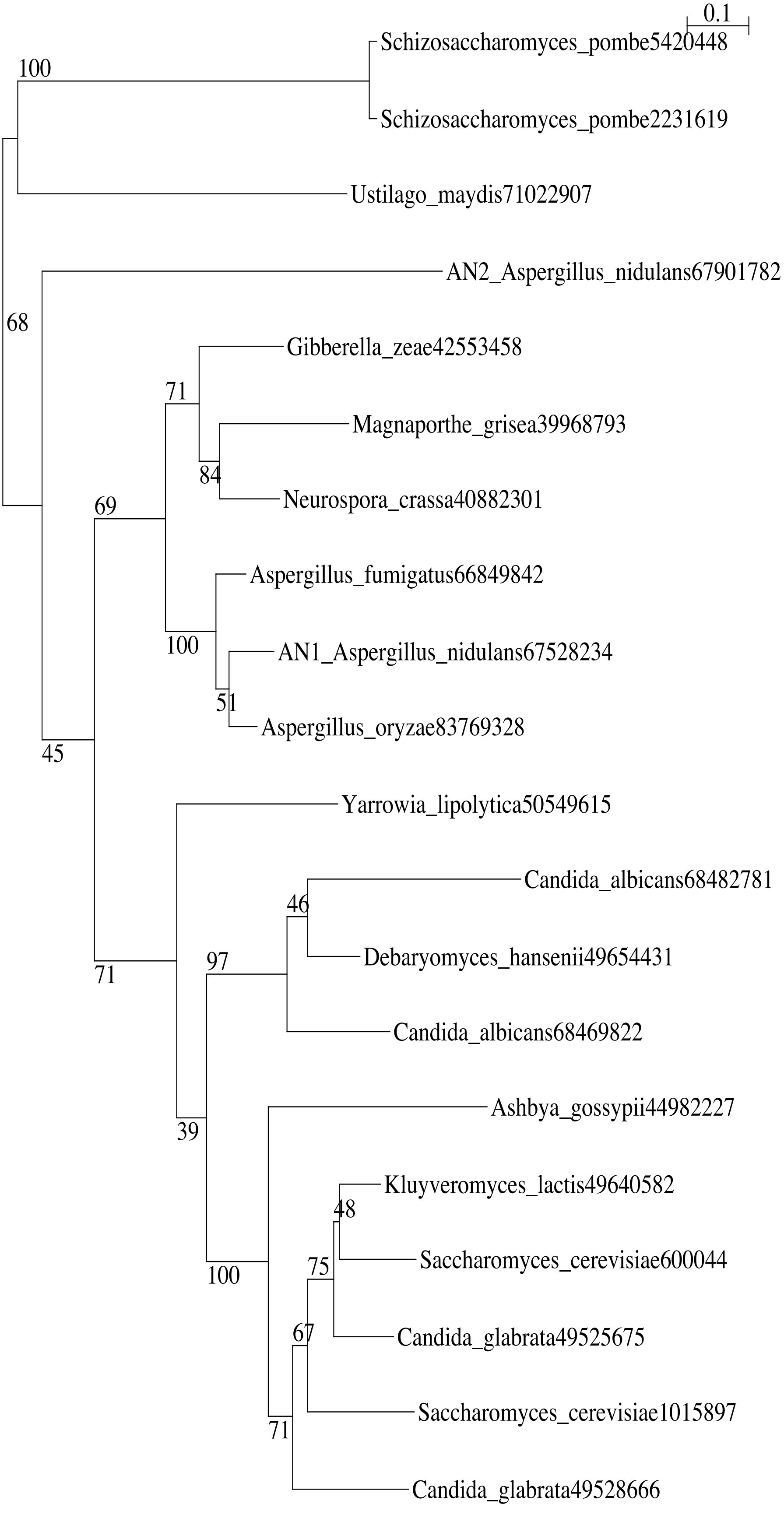
0.1
Schizosaccharomyces_pombe5420448
Schizosaccharomyces_pombe2231619
Ustilago_maydis71022907
AN2_Aspergillus_nidulans67901782
Gibberella_zeae42553458
Magnaporthe_grisea39968793
Neurospora_crassa40882301
Aspergillus_fumigatus66849842
AN1_Aspergillus_nidulans67528234
Aspergillus_oryzae83769328
Yarrowia_lipolytica50549615
Candida_albicans68482781
Debaryomyces_hansenii49654431
Candida_albicans68469822
Ashbya_gossypii44982227
Kluyveromyces_lactis49640582
Saccharomyces_cerevisiae600044
Candida_glabrata49525675
Saccharomyces_cerevisiae1015897
Candida_glabrata49528666
100
68
71
84
69
100
51
45
46
97
71
39
48
75
100
67
71

Trees classified as Topology C in *Aspergillus nidulans* (2).

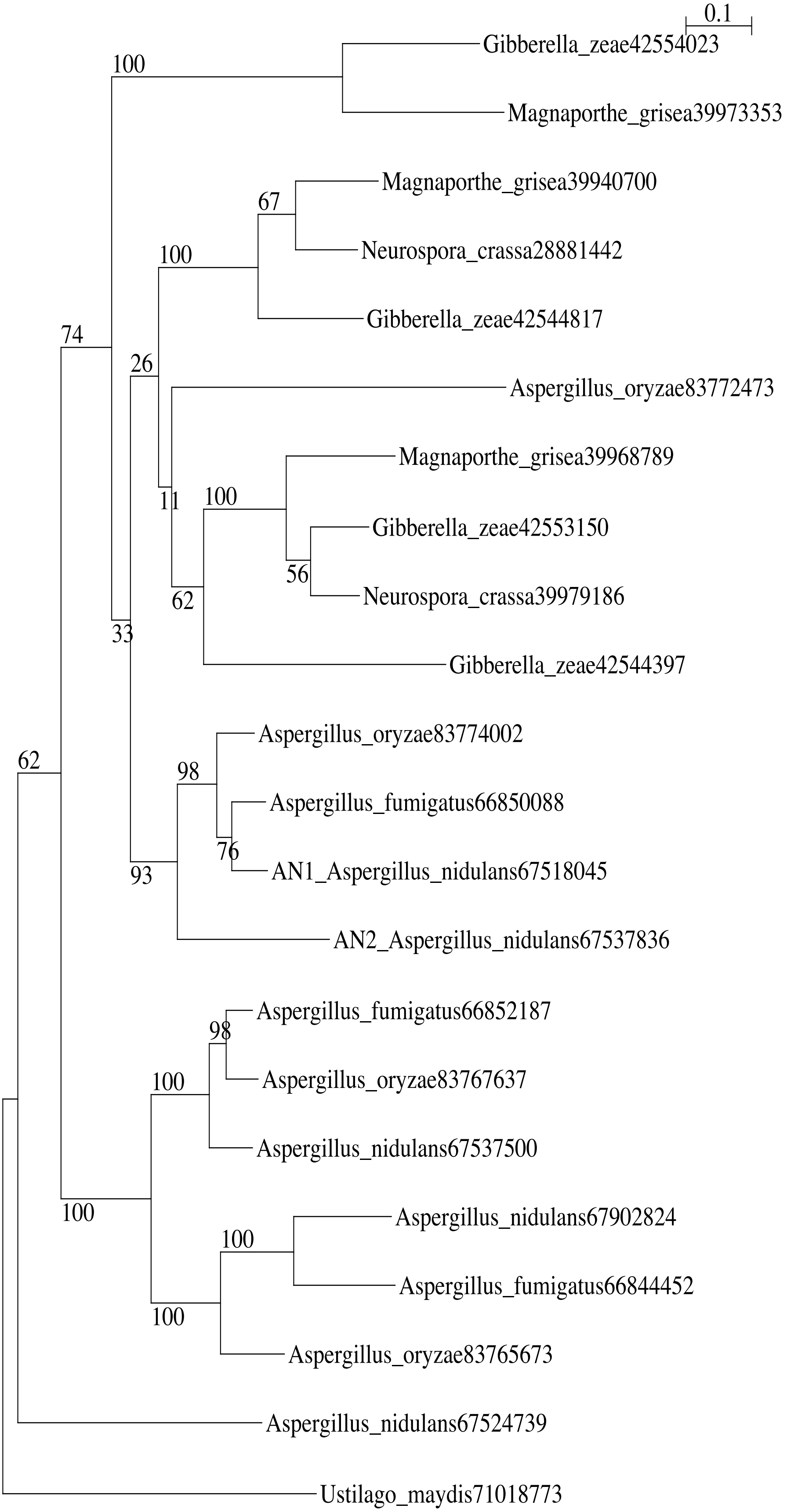
0.1
100
Gibberella_zeae42554023
Magnaporthe_grisea39973353
67
Magnaporthe_grisea39940700
100
Neurospora_crassa28881442
Gibberella_zeae42544817
74
26
Aspergillus_oryzae83772473
Magnaporthe_grisea39968789
11
100
Gibberella_zeae42553150
56
Neurospora_crassa39979186
62
33
Gibberella_zeae42544397
Aspergillus_oryzae83774002
98
Aspergillus_fumigatus66850088
62
76
AN1_Aspergillus_nidulans67518045
93
AN2_Aspergillus_nidulans67537836
Aspergillus_fumigatus66852187
98
Aspergillus_oryzae83767637
100
Aspergillus_nidulans67537500
100
Aspergillus_nidulans67902824
100
Aspergillus_fumigatus66844452
100
Aspergillus_oryzae83765673
Aspergillus_nidulans67524739
Ustilago_maydis71018773

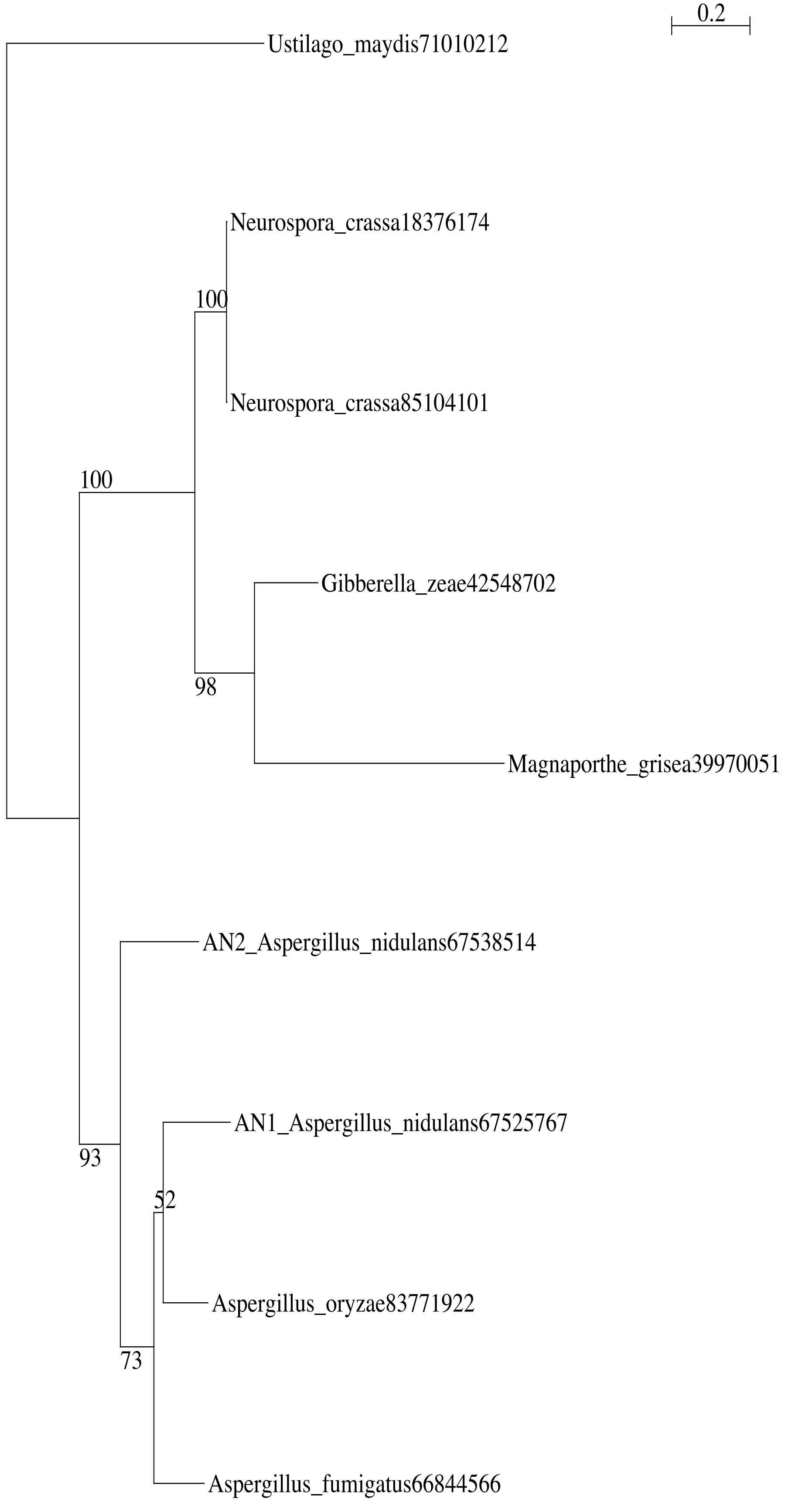
0.2
Ustilago_maydis71010212
Neurospora_crassa18376174
100
Neurospora_crassa85104101
100
Gibberella_zeae42548702
98
Magnaporthe_grisea39970051
AN2_Aspergillus_nidulans67538514
AN1_Aspergillus_nidulans67525767
93
52
Aspergillus_oryzae83771922
73
Aspergillus_fumigatus66844566

Trees classified as Topology B in *Aspergillus fumigatus* (2).

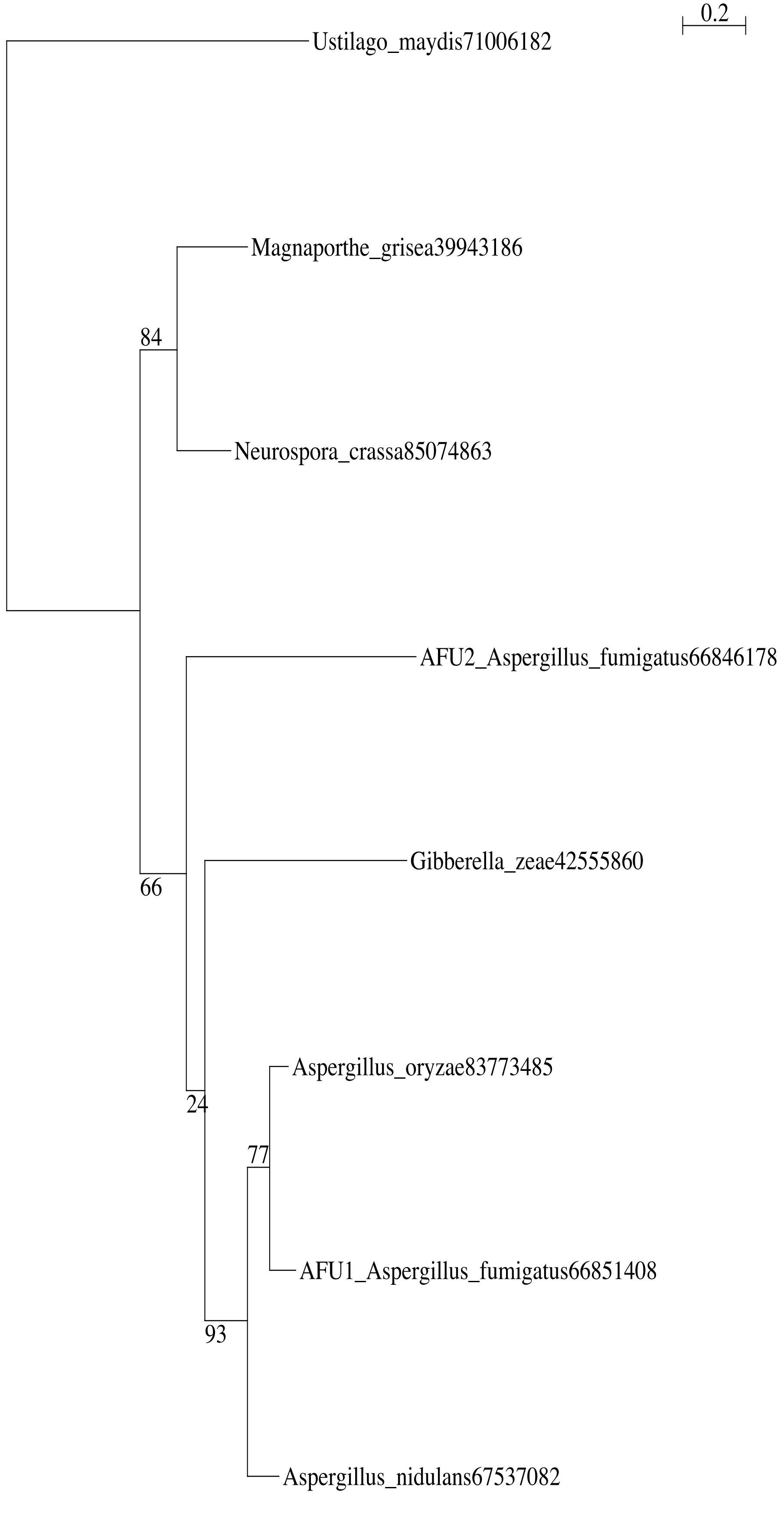

0.2
Ustilago_maydis71006182
Magnaporthe_grisea39943186
84
Neurospora_crassa85074863
AFU2_Aspergillus_fumigatus66846178
Gibberella_zeae42555860
66
Aspergillus_oryzae83773485
24
77
AFU1_Aspergillus_fumigatus66851408
93
Aspergillus_nidulans67537082

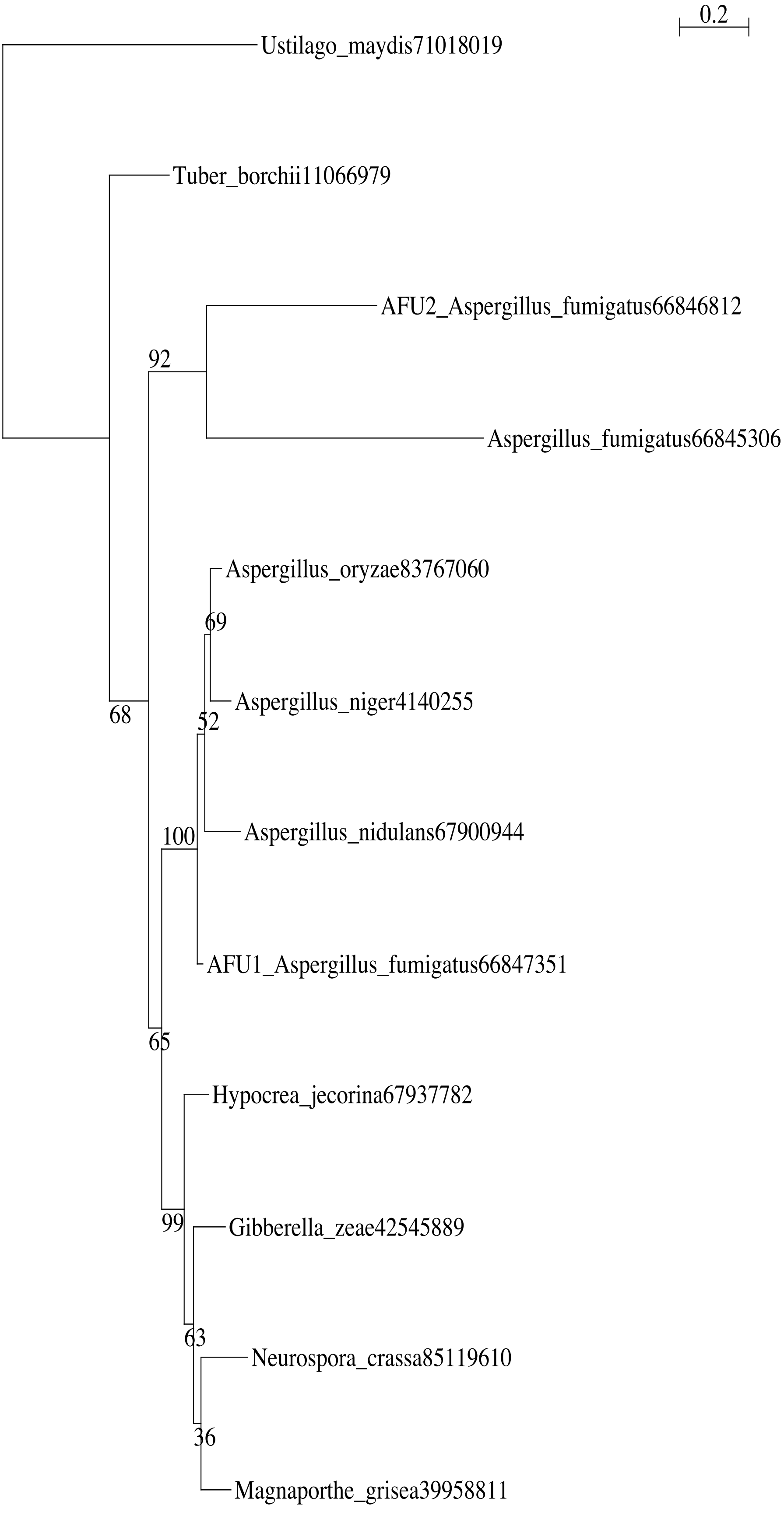
0.2
Ustilago_maydis71018019
Tuber_borchii11066979
AFU2_Aspergillus_fumigatus66846812
92
Aspergillus_fumigatus66845306
Aspergillus_oryzae83767060
69
Aspergillus_niger4140255
68
52
Aspergillus_nidulans67900944
100
AFU1_Aspergillus_fumigatus66847351
65
Hypocrea_jecorina67937782
99
Gibberella_zeae42545889
63
Neurospora_crassa85119610
36
Magnaporthe_grisea39958811

Trees classified as Topology C in *Aspergillus fumigatus* (4).

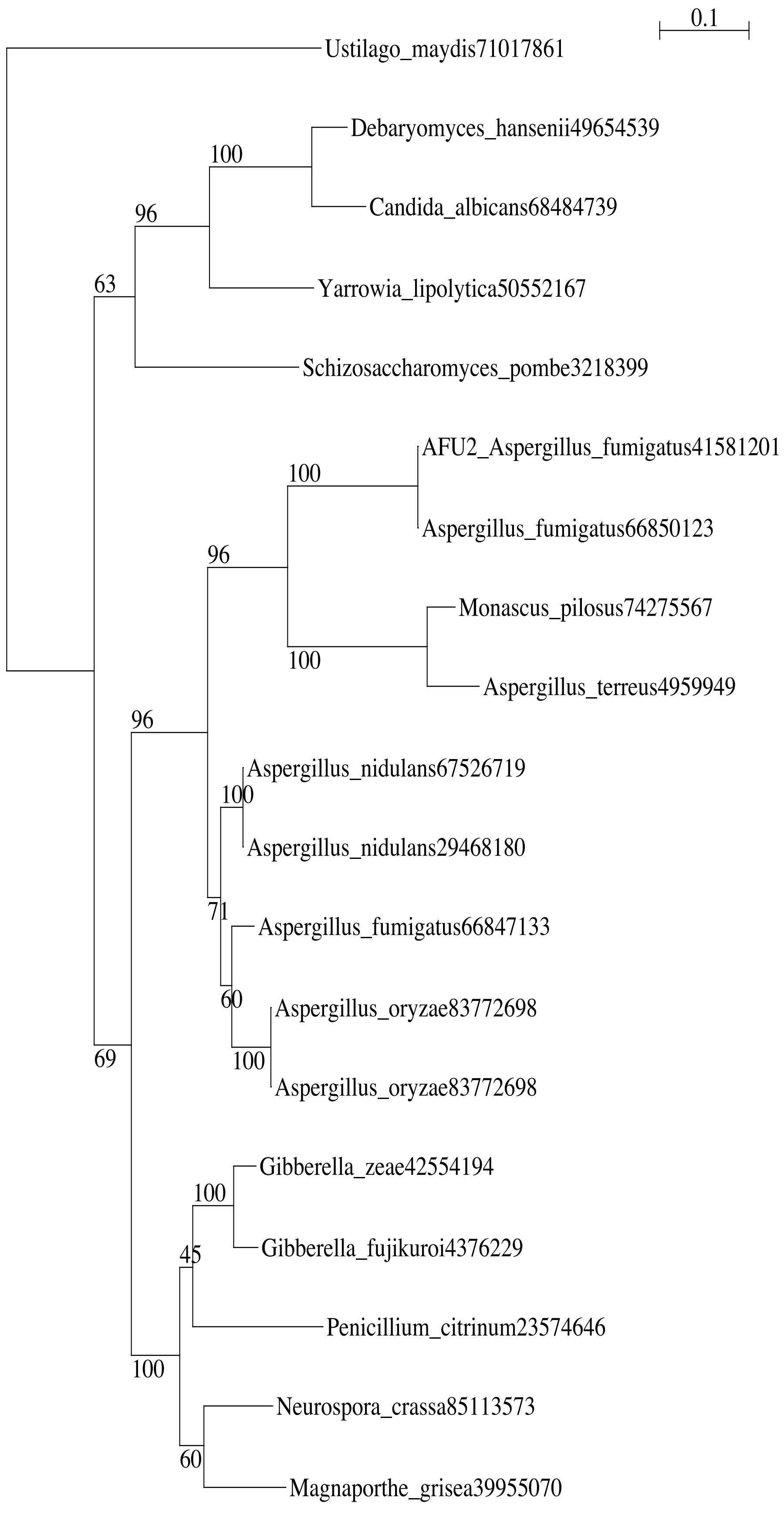

0.1
Ustilago_maydis71017861
Debaryomyces_hansenii49654539
Candida_albicans68484739
Yarrowia_lipolytica50552167
Schizosaccharomyces_pombe3218399
AFU2_Aspergillus_fumigatus41581201
Aspergillus_fumigatus66850123
Monascus_pilosus74275567
Aspergillus_terreus4959949
Aspergillus_nidulans67526719
Aspergillus_nidulans29468180
Aspergillus_fumigatus66847133
Aspergillus_oryzae83772698
Aspergillus_oryzae83772698
Gibberella_zeae42554194
Gibberella_fujikuroi4376229
Penicillium_citrinum23574646
Neurospora_crassa85113573
Magnaporthe_grisea39955070
100
96
63
100
96
100
96
100
71
60
100
69
100
45
100
60

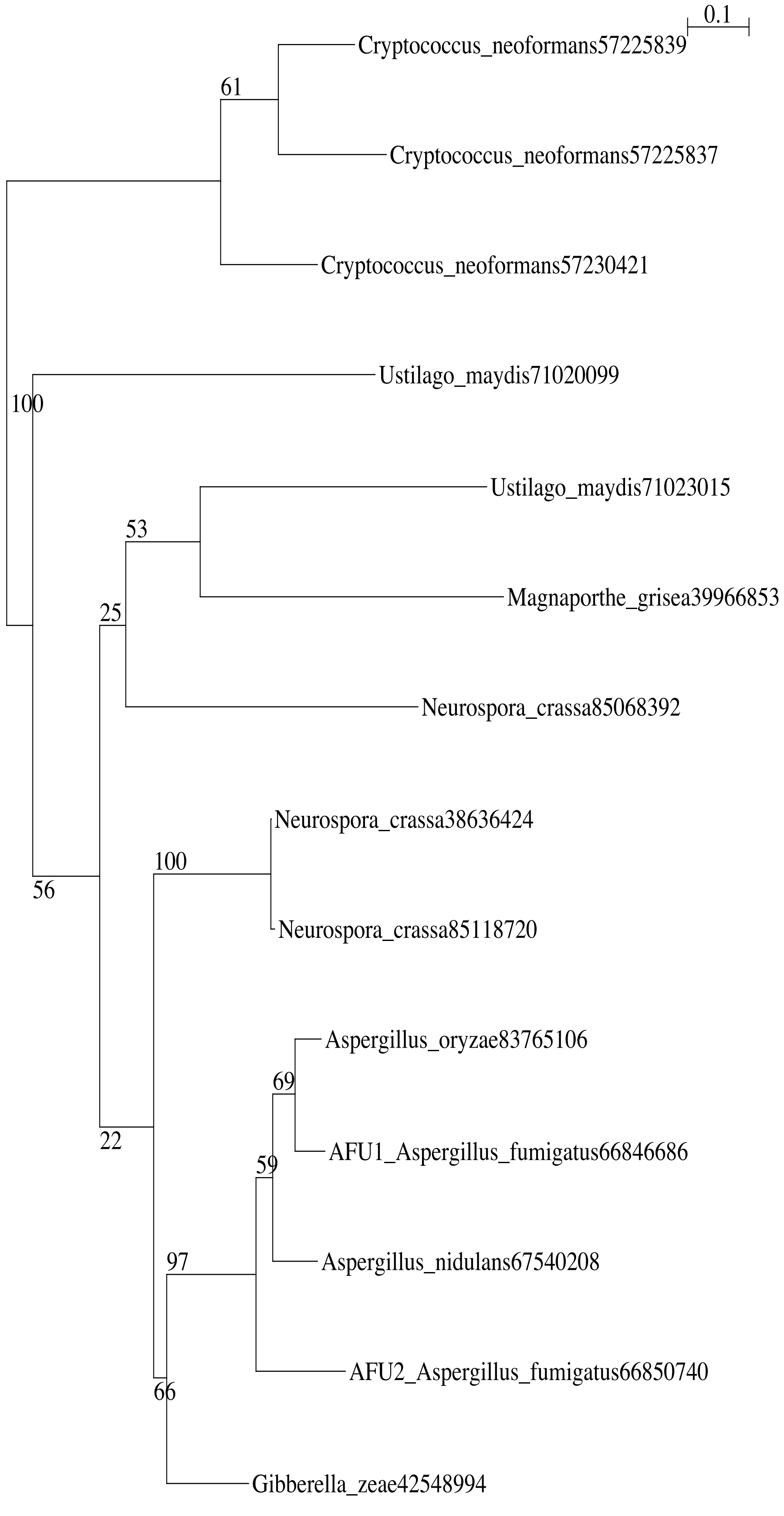
0.1
Cryptococcus_neoformans57225839
61
Cryptococcus_neoformans57225837
Cryptococcus_neoformans57230421
Ustilago_maydis71020099
100
Ustilago_maydis71023015
53
Magnaporthe_grisea39966853
25
Neurospora_crassa85068392
Neurospora_crassa38636424
100
56
Neurospora_crassa85118720
Aspergillus_oryzae83765106
69
22
AFU1_Aspergillus_fumigatus66846686
59
Aspergillus_nidulans67540208
97
AFU2_Aspergillus_fumigatus66850740
66
Gibberella_zeae42548994

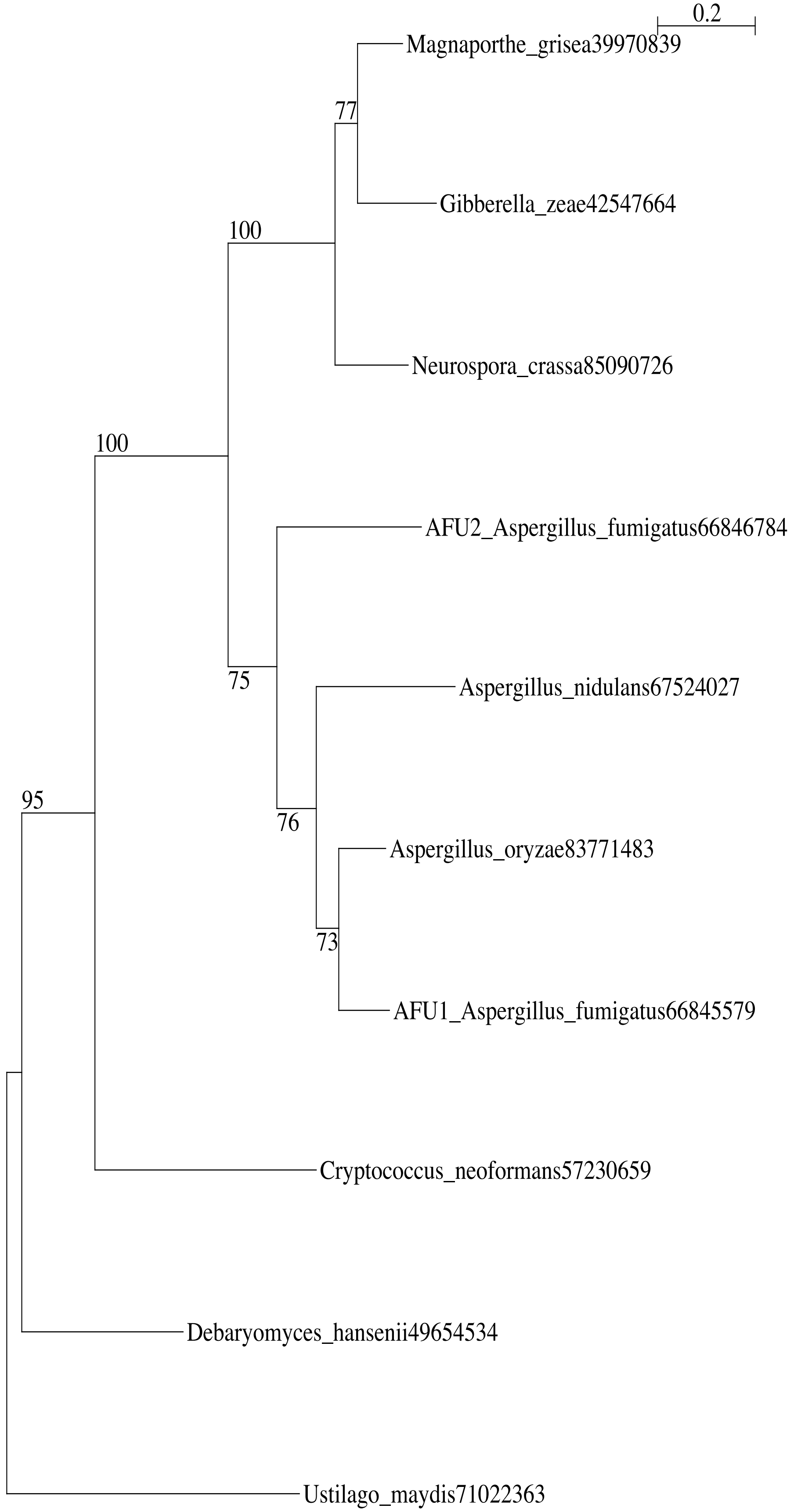
0.2
Magnaporthe_grisea39970839
77
Gibberella_zeae42547664
100
Neurospora_crassa85090726
100
AFU2_Aspergillus_fumigatus66846784
75
Aspergillus_nidulans67524027
95
76
Aspergillus_oryzae83771483
73
AFU1_Aspergillus_fumigatus66845579
Cryptococcus_neoformans57230659
Debaryomyces_hansenii49654534
Ustilago_maydis71022363

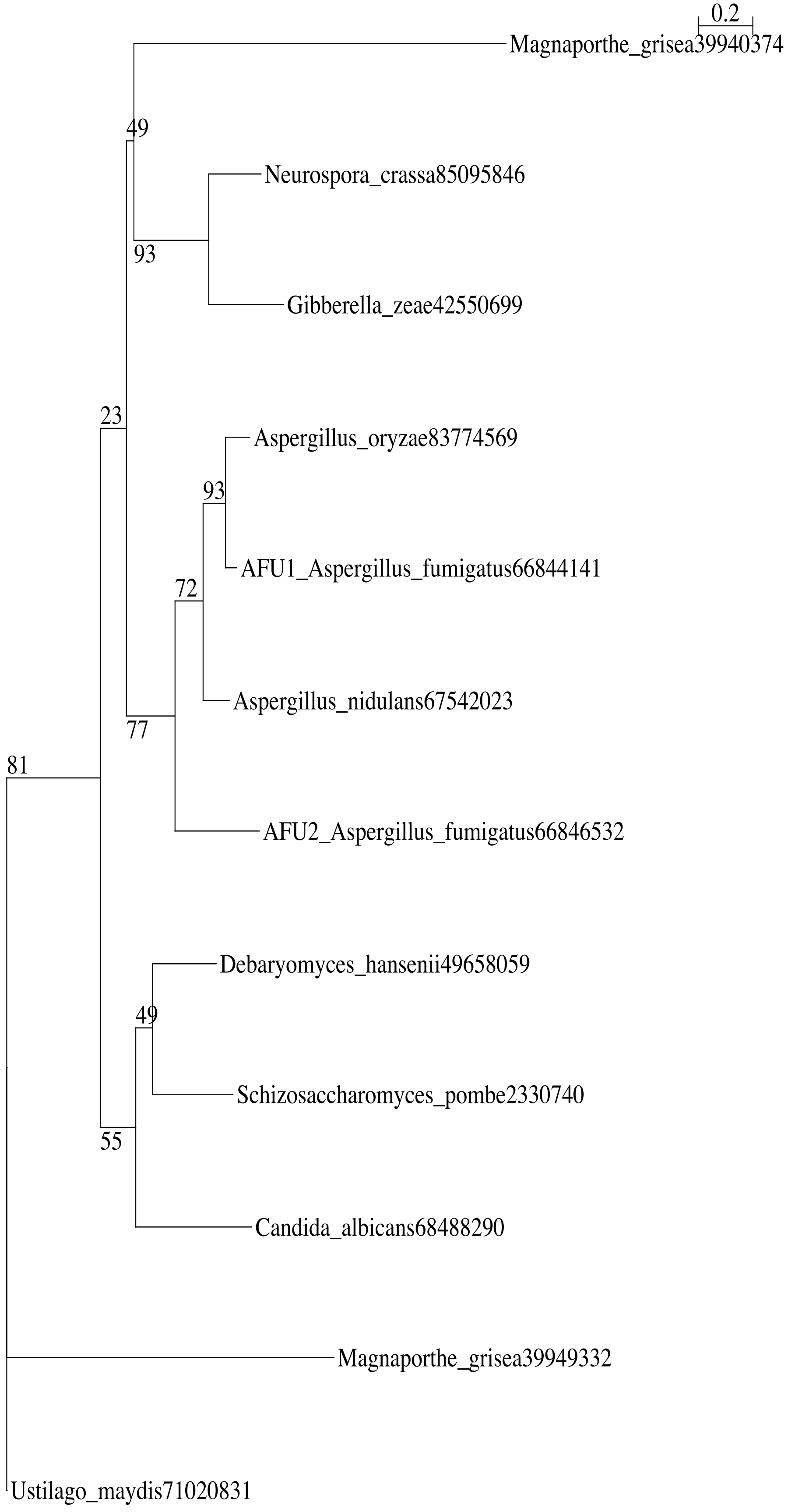
0.2
Magnaporthe_grisea39940374
49
Neurospora_crassa85095846
93
Gibberella_zeae42550699
23
Aspergillus_oryzae83774569
93
AFU1_Aspergillus_fumigatus66844141
72
Aspergillus_nidulans67542023
77
81
AFU2_Aspergillus_fumigatus66846532
Debaryomyces_hansenii49658059
49
Schizosaccharomyces_pombe2330740
55
Candida_albicans68488290
Magnaporthe_grisea39949332
Ustilago_maydis71020831
